# Care Around Birth Approach: A Training, Mentoring, and Quality Improvement Model to Optimize Intrapartum and Immediate Postpartum Quality of Care in India

**DOI:** 10.9745/GHSP-D-20-00368

**Published:** 2021-09-30

**Authors:** Gunjan Taneja, Enisha Sarin, Devina Bajpayee, Saumyadripta Chaudhuri, Geeta Verma, Rakesh Parashar, Nidhi Chaudhry, Jaya Swarup Mohanty, Nitin Bisht, Anil Gupta, Shailendra Singh Tomar, Rachana Patel, V.S. Sridhar, Anurag Joshi, Chitra Rathi, Dinesh Baswal, Sachin Gupta, Rajeev Gera

**Affiliations:** a United States Agency for International Development-Vriddhi (Scaling up RMNCH+A Interventions) Project, New Delhi, India.; b IPE Global, New Delhi, India.; c Independent consultant, New Delhi, India.; d Ministry of Health and Family Welfare, Government of India, New Delhi, India.; e Maternal and Child Health, United States Agency for International Development–India, New Delhi, India.

## Abstract

**Background:**

With the highest risk of maternal and newborn mortality occurring during the period around birth, quality of care during the intrapartum and immediate postpartum periods is critical for maternal and neonatal survival.

**Methods:**

The United States Agency for International Development’s Scaling Up Reproductive, Maternal, Newborn, Child, and Adolescent Health Interventions project, also known as the Vriddhi project, collaborated with the national and 6 state governments to design and implement the Care Around Birth approach in 141 high caseload facilities across 26 high-priority districts of India from January 2016 to December 2017. The approach aimed to synergize evidence-based technical interventions with quality improvement (QI) processes, respectful maternity care, and health system strengthening efforts. The approach was designed using experiential training, mentoring, and a QI model. A baseline assessment measured the care ecosystem, staff competencies, and labor room practices. At endline, the approach was externally evaluated.

**Results:**

Availability of logistics, recording and reporting formats, and display of protocols improved across the intervention facilities. At endline (October–December 2017), delivery and newborn trays were available in 98% of facilities compared to 66% and 55% during baseline (October–December 2015), respectively. Competency scores (> 80%) for essential newborn care and newborn resuscitation improved from 7% to 70% and from 5% to 82% among health care providers, respectively. The use of partograph in monitoring labor improved from 29% at the baseline to 61%; administration of oxytocin within 1 minute of delivery from 35% to 93%; newborns successfully resuscitated from 71% to 96%; and postnatal monitoring of mothers from 52% to 94%.

**Conclusion:**

The approach successfully demonstrated an operational design to improve the provision and experience of care during the intrapartum and immediate postpartum periods, thereby augmenting efforts aimed at ending preventable child and maternal deaths.

## Abstract (precis)

The Care Around Birth approach provides an integrated implementation framework to improve the quality, equity, and dignity of care during the intrapartum and immediate postpartum periods, thereby addressing key drivers of maternal and newborn mortality.

## Teaser

**Key Findings:**

- Adopting an integrated implementation framework that combined training, mentoring, and quality improvement processes to improve the quality, equity, and dignity of care during the intrapartum and immediate postpartum periods, the Care Around Birth approach addressed key drivers of maternal and newborn mortality.
- The approach refocused energy on facility-based quality processes that are currently central to efforts in reducing mortality and achieving Sustainable Development Goal targets.

**Key Implications:**

- Integrated implementation frameworks need to be aligned to the resources available and tailored to the ecosystem in which health programs operate.
- Facility-level ownership and accountability by optimizing the engagement of health staff remain the fulcrum for any improvement effort. This however needs to be adequately supported by district and state-level health systems.

## INTRODUCTION

Three recent global reports prioritize the importance of quality health services, the impact of not providing quality care to clients, and necessary measures to improve it.^1^^–^^3^ Although poor quality of care has an impact across all dimensions of the health systems, possibly nowhere is it more acute than during the intrapartum and immediate postpartum periods. The World Health Organization (WHO) estimates that 303,000 mothers and 2.7 million newborn infants die annually around the time of childbirth and that many more are affected by preventable illnesses. Further, some 2.6 million babies are stillborn each year.^4^^–^^5^ Hence, the time of childbirth and the period immediately after birth are particularly critical for maternal, fetal, and neonatal survival and well-being. In addition, a United Nations report estimated that providing high-quality care during childbirth could prevent approximately 113,000 maternal deaths, 531,000 stillbirths, and 1.3 million neonatal deaths every year at an estimated cost of $4.5 billion per year ($0.9 per person).^6^

In line with global trends, maternal and newborn mortality remains a major challenge in India. Though the maternal mortality ratio and the neonatal mortality rate have seen an appreciable decline over the past 2 decades,^7^^–^^8^ 32,000 maternal and 640,000 newborn deaths still occur every year in the country.^9^^–^^10^ Most of these deaths occur during the period around birth, but efforts to improve access to care, such as Janani Suraksha Yojna (a Safe Motherhood scheme), have not resulted in a concomitant decline in mortality.^11^^–^^12^ Thus, it is imperative that India focuses on quality of care at and around the time of birth for the mother and baby dyad. Although the Government of India has undertaken multiple efforts toward improving the quality of care through various national policy initiatives, the real challenge is translating operational guidelines and plans to on-the-ground implementation.^13^^–^^16^

The Government of India has made efforts to improve the quality of care through various national policy initiatives, but the challenge of translating operational guidelines and plans to on-the-ground implementation remains.

## VRIDDHI PROJECT

Prioritizing the need to improve access, utilization, and quality of care, in 2013, the Government of India launched the Reproductive, Maternal, Newborn, Child, and Adolescent Health (RMNCH+A) Strategy.^17^ To facilitate focused planning and implementation, the strategy identified 184 underperforming districts as high-priority districts. Overall, within each state, 25% of the poorest-performing districts were identified. The tribal predominant districts and those affected by insurgent and rebel groups were also selected. Thus, the strategy primarily aimed to address geographical inequity by prioritizing districts that had traditionally performed suboptimally. Further, the strategy obtained technical assistance from partner agencies to support its rollout and implementation. Thus USAID, through the Vriddhi Scaling up RMNCH+A Interventions) project (hereafter referred to as the project), implemented by IPE Global in collaboration with the national Ministry of Health and Family Welfare (MOHFW), supported 26 high-priority districts in the states of Delhi, Haryana, Himachal Pradesh, Jharkhand, Punjab, and Uttarakhand.

## CARE AROUND BIRTH APPROACH INTERVENTION

Aligning with global priorities, WHO’s Quality of Care framework for maternal and newborn health, and national guidelines,^18^ the project designed and implemented the Care Around Birth approach, a strategic intervention to improve clinical practices. This approach combines essential evidence-based technical interventions and health system strengthening efforts through quality improvement techniques to improve the quality of care at and around the time of birth. The intervention was implemented from January 2016 to December 2017 in 141 high caseload public health facilities across these 26 high priority districts in the 6 states and reached 463,713 mothers and 458,152 newborns.

**FIGURE 1:**
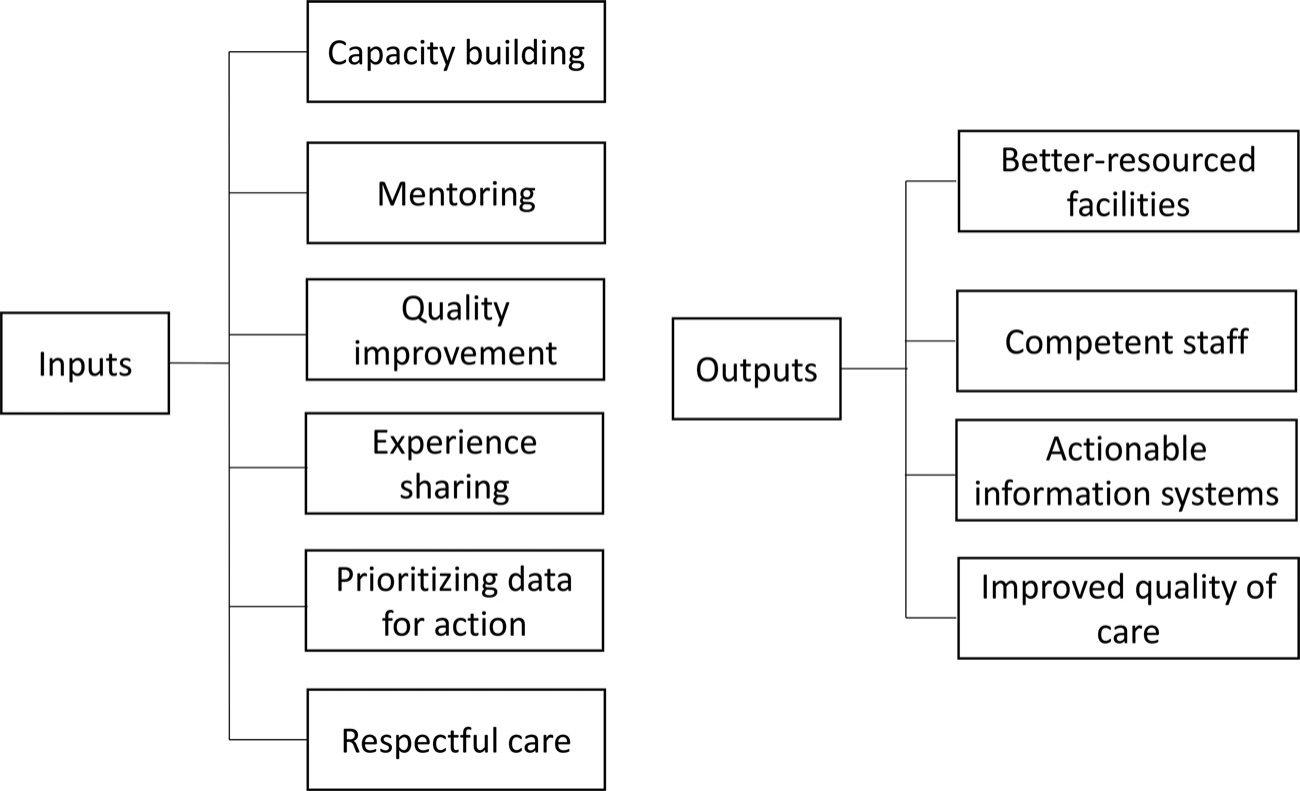
Care Around Birth Approach Framework to Improve Quality of Care in 6 States in India

The intervention, which was externally evaluated, included building a robust and scalable model and catalyzing and measuring the change (Figure 1).^19^

### Building a Robust, Scalable Implementation Model

Because implementation was planned across 6 states that varied considerably in sociodemographic and health parameters, adequate time was spent on designing and conceptualizing the approach (Table 1).

**TABLE 1.** Health and Sociodemographic Indicators Across Intervention States Included in Care Around Birth Approach Intervention

**Indicator**	**States**						**India**
	**Delhi**	**Haryana**	**Himachal Pradesh**	**Jharkhand**	**Punjab**	**Uttarakhand**	
MMR (SRS MMR bulletin 2014–16)	NA	101	NA	165	122	201	130
NMR (SRS statistical report 2015)	14	24	19	23	13	28	25
Infant Mortality Rate (SRS statistical report 2015)	18	36	28	32	23	34	37
Total Fertility Rate (SRS statistical report 2015)	1.7	2.2	1.7	2.7	1.7	2	2.3
Sex ratio at birth (SRS statistical report 2015)	869	831	924	902	889	844	900
**The below indicators are cited from NFHS 4 (2015–2016)**							
Women age 20–24 years married before age 18 years, %	14.3	19.4	8.6	37.9	7.6	13.8	26.8
Current use of a modern method of family planning, %	48.6	59.4	52.1	37.5	66.3	49.3	47.8
Unmet need for family planning, %	15	9.3	15.7	18.4	6.2	15.5	12.9
Mothers who had antenatal check-up in the first trimester, %	63	63.2	70.5	52	75.6	53.5	58.6
Mothers who had at least 4 antenatal care visits, %	67.9	45.1	69.1	30.3	68.5	30.9	51.2
Mothers who had full antenatal care, %	39	19.5	36.8	8	30.7	11.5	21
Institutional births, %	84.4	80.4	76.4	61.9	90.5	68.6	78.9
Institutional births in public facility, %	55.5	52	61.6	41.8	51.7	43.8	52.1
Births delivered by cesarean delivery, %	26.7	11.7	16.7	9.9	24.6	13.1	17.2
Births in a public health facility delivered by cesarean delivery, %	26.5	8.6	16.4	4.6	17.8	9.3	11.9
Children aged 12–23 months fully immunized (BCG, measles, and 3 doses each of polio and DPT), %	68.8	62.2	69.5	61.9	89.1	57.6	62
Children under age 3 years breastfed within 1 hour of birth, %	28	42.4	41.1	33.1	30.7	27.8	41.6
Children under age 6 months exclusively breastfed, %	49.6	50.3	67.2	64.8	53	51.2	54.9
Children aged younger than 5 years who are stunted (height-for-age), %	31.9	34	26.3	45.3	25.7	33.5	38.4
Children aged younger than 5 years who are wasted (weight-for-height), %	15.9	21.2	13.7	29	15.6	19.5	21
Pregnant women aged 15–49 years who are anemic (<11.0 g/dl), %	46.1	55	50.4	62.6	42	46.5	50.4
Women having a bank or savings account that they use, %	64.5	45.6	68.8	45.1	58.8	58.5	53
Women having a mobile phone that they use, %	66.6	50.5	73.9	35.2	57.2	55.4	45.9
Women aged 15–24 years who use hygienic methods of protection during their menstrual period, %	90.7	78.3	84.3	49.6	84.4	69.9	57.6

Abbreviations: DPT, diphtheria, pertussis, tetanus vaccine; MMR, maternal mortality rate; NFHS, national family health survey; NMR, neonatal mortality rate; SRS, Sample Registration Survey.

Definitions: Maternal Mortality Ratio: The number of maternal deaths during a given period per 100,000 live births during the same period. Neonatal Mortality Rate: The number of neonatal deaths (deaths during the first 28 days of life) per 1000 live births. Infant Mortality Rate: The number of infant deaths (deaths during the first year of life) per 1,000 live births.

Total Fertility Rate: The number of children who would be born per woman (or per 1,000 women) if she/they were to pass through the childbearing years bearing children according to a current schedule of age-specific fertility rates. Sex Ratio at Birth: The number of girls born for every 1,000 boys born. Unmet need for family planning: The percentage of women of reproductive age, either married or in a union, who have an unmet need for family planning. Women with unmet need are those who want to stop or delay childbearing but are not using any method of contraception.

The project team engaged in multiple rounds of discussions to comprehensively define the scope and spectrum of the intervention including the geographical reach, the identification of facilities, the care practices to be supported, and the measurement indicators to be tracked. The team developed a robust and comprehensive assessment framework for establishing baselines across the facilities. Then, we finalized the care practices to be strengthened, tailored the technical intervention package to be delivered using adult learning principles, adapted the quality improvement (QI) processes for implementation at scale, and envisioned an actionable management information system. The preparatory phase also included training of the project team on all the components. The design phase culminated in advocacy workshops across the 6 states led by the respective state governments to disseminate the baseline findings, develop facility improvement plans, and initiate the implementation phase of the approach.

### Catalyzing Change

The Care Around Birth approach integrated training, mentoring, and QI aspects to potentiate change. Identifying staff competencies as a major barrier to implementation of quality, high-impact interventions at scale, the approach included capacity building to ensure that health workers were not only well-trained on clinical, programmatic, and managerial aspects but also competent to provide high-quality services.

The Care Around Birth approach included capacity building to ensure that health workers were not only well-trained on clinical, programmatic, and managerial aspects but also competent to provide high-quality services.

#### Experiential Trainings

A participant-centric, experiential training package based on adult learning principles was designed to address the knowledge and practice gap and help facility staff overcome barriers in translating their knowledge into action.^20^ The training content was adapted from relevant national guidelines and focused on delivering high-impact technical interventions to mothers and newborns during childbirth to manage complications, giving clear guidance on processes and protocols. Overall, the trainings improved competency on 14 technical interventions, including monitoring the progress of labor, active management of the third stage of labor; essential newborn care, newborn resuscitation, postnatal monitoring of mothers and newborns, management of postpartum hemorrhage and pre-eclampsia/eclampsia, kangaroo mother care, assisted feeding of low birth weight babies, use of antenatal corticosteroids for preterm labor, management of maternal and newborn sepsis, and the prevention of mother-to-child transmission of HIV. The trainings followed the sequence of birth to promote seamless care for mothers and newborns. Simulation-based drills were used to build competencies for managing complications with cross-cutting issues of health systems strengthening, documentation, labor room organization, and respectful maternity care (RMC) integrated into the package.

The training methods included simulations, demonstrations, case studies, role playing, and other participatory methods. The choice of methods was guided by factors enabling spontaneous participation of all participants; building on participants’ experiences, views, and beliefs; encouraging peer learning; and allowing for self-assessment of knowledge and skills. The training session flow was adapted to fulfill these criteria and included the following:

- Establishing a learning need: Sessions always started with establishing a learning need by allowing participants to realize the gap in knowledge or practice. This was done through structured questions and answers, writing cards, and demonstrating existing practices.
- Building on existing knowledge: Participants’ responses were valued, and facilitators provided additional new information only after summing up the group’s inputs. This helped to build upon existing knowledge and further highlighted the learning need.
- Learning by observation: The correct practice was demonstrated by the facilitator; the participants observed the demonstration and were encouraged to note differences from their current practice.
- Doing, observing, and critiquing: All participants were given an opportunity to practice individually under supervision in small groups. In addition to the group and facilitator, all co-participants observed the practice. This further added to the learning experience.
- Sharing problems, challenges, and solutions: This step allowed participants to reflect on what they had learned and visualize challenges and issues in implementing the new practice, the process facilitated by peer-to-peer learning. At this stage, problems related to larger systemic issues such as supplies, infrastructure, etc., which were beyond the scope of the training, were also listed to be discussed subsequently with district and state authorities.
- Performing the new skill: At the end of each session, participants demonstrated their newly learned skills and made commitments to continue the practice.

The trainings were centralized, held at the district level over 2 days, and were followed by planned, structured, and low-density, high-frequency on-site training sessions at health facilities to ensure saturation of all health care providers at the intervention facilities. The trainings were facilitated by the project team, which included professionals with relevant experience in maternal and child health.

#### On-Site Mentoring

The project team conducted post-training follow-up facility visits to mentor and support health care providers, resolve doubts, and facilitate practice to boost confidence and enable local problem solving.^21^ Drills and checklists were introduced to maintain a high level of preparedness among staff to handle maternal and newborn complications. Job aids and notes were provided to labor room staff to remind them of actions and interventions, and peer-to-peer learning opportunities were created by highlighting good practices. The consistent emphasis on data for action during the mentoring visits helped tailor the visits to the specific needs of the health workers and facilities, enabling improved record maintenance and inculcating the practice of evidence-based care.

#### QI Processes

The momentum for change was sustained by introducing QI processes at the facility level.^22^ QI efforts were built into the model and were optimized as the fulcrum for maintaining and sustaining changes over the intervention period. The project facilitated the process of institutionalizing QI in health facilities by forming QI teams and using a data-driven approach to problem solving. Core members of the QI teams included facility managers, gynecologists, pediatricians, and head staff nurses. Auxiliary members, such as pharmacists, storekeepers, and accountants were included according to the improvement aim identified. QI processes enabled the teams to start addressing challenges as a group, and these teams instituted at facilities took up facility-level unresolved issues, identified bottlenecks, brainstormed to find viable solutions, fixed responsibilities and timelines, and eventually accelerated change. The teams usually met monthly to identify gaps based on service statistics, suggest ideas for change, and track progress. Initial handholding support and coaching were provided by project staff. QI teams developed and tested ideas using the plan-do-study-act approach for continual improvements to service quality.^23^

QI efforts were built into the model and were optimized as the fulcrum for maintaining and sustaining changes over the intervention period.

#### Experience-Sharing Platforms

The facility-level QI efforts were complemented by district- and state-level experience-sharing platforms, which brought together facility staff at the district or state levels to share learnings, challenges, and solutions. This further facilitated peer-to-peer engagement and also served as a way to acknowledge good performers.

#### Imbibing RMC

**FIGURE 2:**
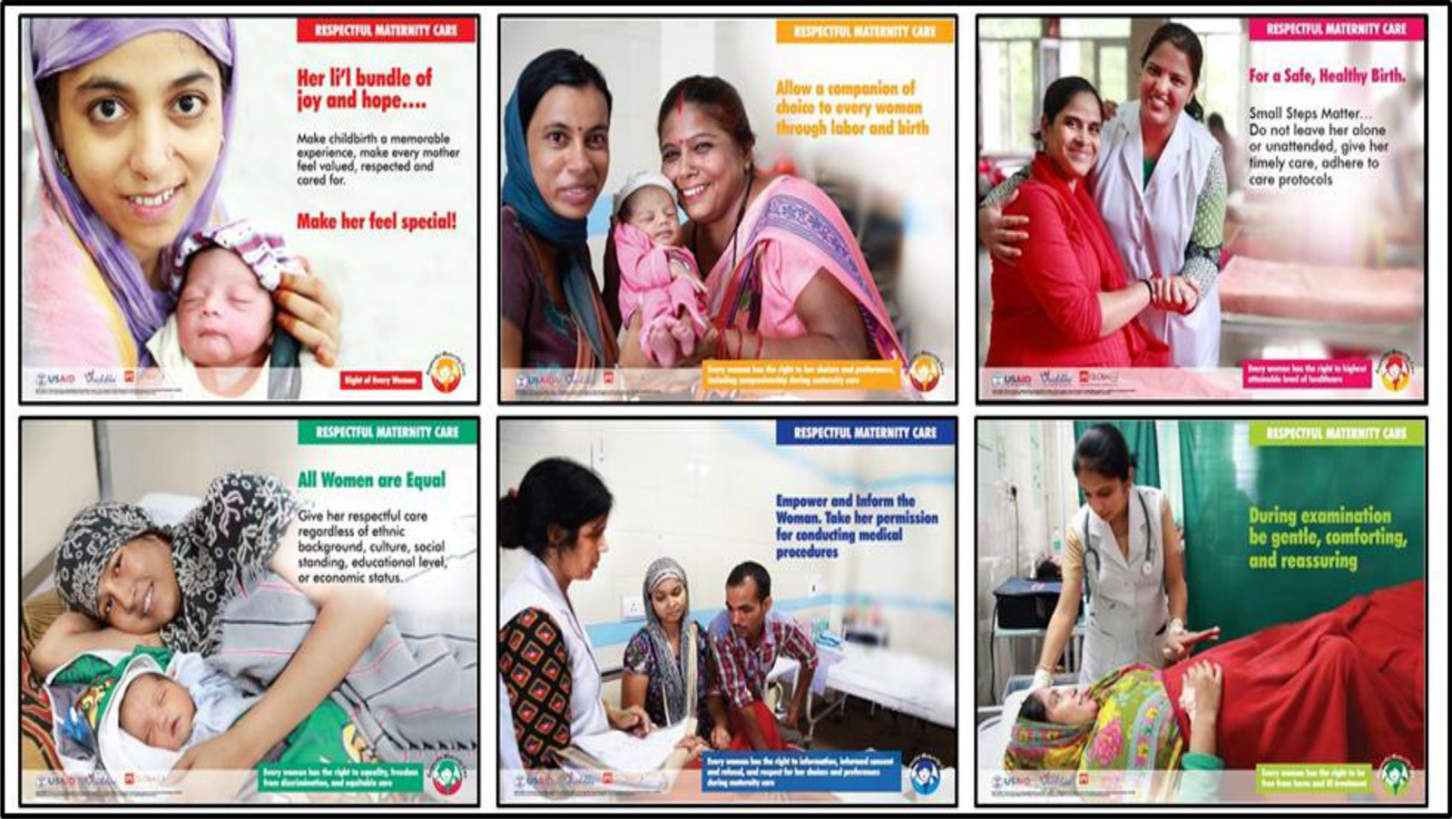
Job Aids to Encourage Providers to Practice Respectful Maternity Care at Intervention Facilities in 6 States in India

To prioritize RMC, the project team ensured a provider-centric RMC framework that included institutionalizing RMC practices during various components of care. The project team identified RMC as integral to its outcomes, both in project design and implementation, and undertook a situational analysis in select public health facilities to understand their readiness to provide RMC. The project team aimed to improve the experience of care at the intervention facilities by including RMC practices within the training curriculum, engaging with health care providers on it during mentoring visits and QI meetings, and developing job aids for display at the intervention facilities (Figure 2).

### Measuring Change

Data management and usage were built on USAID’s Collaborating Learning and Adapting framework, which ensured that the intervention was coordinated with other parallel efforts, grounded in a strong evidence base, and iteratively adapted.^24^ Data systems were set up during the conceptualization and design phase and evolved during the implementation phase to ensure data-based decision making. The systems were built upon existing government systems, and relevant and appropriate process level indicators were incorporated. The overall effort included standardizing labor room registers and case sheets, regular review of records with emphasis on complete and accurate recording, internal data validation processes including data review, on-site data validation, and triangulation of data from multiple sources to meet requirements at the facility, district, and state levels. The government staff working at the facility and district levels were involved in this process, and data management processes were strengthened at the intervention facilities during the mentoring visits by the project staff.

## METHODS

### Selection of Intervention Facilities

Identification of intervention facilities was based on the line-listing of public health facilities that had reported at least 1 delivery through the health management information system for the year 2013–2014. More than 1,400 health facilities in the 26 districts had reported deliveries during the year. These facilities were then enlisted by the district. An average of 5–7 high caseload facilities—accounting for close to 70% of the institutional delivery load in public health facilities in the district—were identified for the intervention. Except for 2 primary health centers in Himachal Pradesh, all of the intervention facilities were either district hospitals, first referral unit community health centers, non-first referral unit community health centers, referral hospitals, or subdivisional hospitals. Operationally, 69 intervention facilities were L2 and 72 L3 level facilities^25^ (Table 2).

**TABLE 2.** Categorization of Health Facilities in India Included in Care Around Birth Approach Intervention Implemented in 6 States

**Type and Level of Facility**	**Population Served**	**Beds**	**Human Resource (Also Calculated Based on Caseload)**	**Maternal Health Services**	**Newborn Care**	**Family Planning Services**
Subcenters, Level 1 Primary health care center, not operational 24x7, Level 1	5,000 people or 3,000 in remote areas 30,000 or more people (20,000 in remote areas)	1–2 inpatient/observation beds 6 inpatient/observation beds	2 auxiliary nurse-midwives 1 part-time female sweeper	• Identification and referral for danger signs • Pregnancy testing and counseling• Antenatal care• Intranatal care• Normal deliveries by skilled birth attendant (Partograph, active management of the third stage of labor, etc.)• Pre-referral management for obstetric emergencies (Eclampsia, postpartum hemorrhage, shock)• Postnatal care– 24–48 hours stay post-delivery• Immediate newborn care – drying, warming, skin-to-skin contact• Initiation of breastfeeding• Postpartum contraceptive counseling	**Newborn Care Corner** • Essential newborn care including resuscitation • Zero day immunization (OPV, BCG, Hepatitis B; as per Government of India schedule), Inj. Vit. K **Care of normal newborn** • Breastfeeding/ feeding support, thermo-regulation, and asepsis **Care of sick newborn** • Identification, stabilization, and initial management of complications (sepsis, low birth weight/premature babies, etc.) before referral and prompt referral of “sick” newborn • Referral services	Counseling and provision of spacing methods including interval intrauterine device
Primary health care center operational 24x7, Level 2Non-first referral unit - community health care center, Level 2	30,000 or more people (20,000 in remote areas) 120,000 people in urban areas or 80,000 people in remote areas	6 inpatient/observation beds 30 bedded	1–2 Medical officers (on-call after outpatient department hours) Minimum 4 staff nurses/auxiliary nurse-midwives each for labor room and maternity ward 2 Lab technicians (for round-the-clock service delivery) Sweeper–3 for labor room (preferably female) and maternity ward HR for NBSU	**All services listed in facilities above in Level 1, plus the following:** Assisted vaginal deliveries Management of complications other than those requiring referral to L3 including blood transfusion or surgery Episiotomy and suturing Stabilization of obstetric emergencies and referral to L3 wherever required Antenatal steroids for preterm labor HIV screening 48 hours stay postdelivery Comprehensive abortion care Case management of sexually transmitted infection Antibiotics for preterm or premature rupture of membranes for prevention of sepsis of newborns	**NBSU (Newborn Stabilization Unit)** ***All newborn care services those in Level 1, plus the following:*** **Care of sick newborn** • Identification and Management of LBW infants >/= 1800 gm with no other complications • Phototherapy for newborns with hyperbilirubinemia • Management of newborn sepsis • Stabilization and referral of sick newborns and those with very low birth weight	**All Services provided in Level 1, plus the following:** Female sterilization including post-partum sterilization, male sterilization (conventional and no-scalpel vasectomy)
First referral unit community health center, Level 3 Subdistrict hospital / referral hospital, Level 3District hospital, Level 3	120,000 people in urban areas or 80,000 people in remote areas 100,000 - 500,00 population All population in the district (35,000 to 3,000,000)	30 bedded 31–50 bedded for small population (100,000) – 51–100 beds for large population (500,000) 300 beds for 1,000,000 people population; As the population increase the recommended beds increase to a maximum of 500 beds	Specialists including gynecologist/ emergency obstetric care, anesthetist/LSAS, pediatrician Medical officers Staff nurse, cleaning staff, counselor, lab technician 1 certified sonologist (on call after routine hours) HR for SNCU	**All services listed in facilities above in Level 1 and 2, plus the following:** Comprehensive management of all obstetric emergencies, e.g., PIH/eclampsia, sepsis, PPH, retained placenta, shock, obstructed labor, severe anemia Cesarean delivery and other surgical interventions Blood bank/storage center Blood grouping and cross-matching Link ART/ART at district hospital	**Sick newborn care unit *All newborn care services those in Level 1 and 2, plus the following:*** **Care of sick newborn** Identification and management of LBW infants >/= 1800 gm Management of all sick newborns (except those requiring mechanical ventilation and major surgical interventions) Follow-up of all babies discharged from the unit and including of high-risk newborns	**All services provided in Level 1 and 2, plus the following:** Laparoscopic sterilization Postpartum intrauterine device insertion

Abbreviations: ART, antiretroviral therapy; LSAS, lifesaving anesthetic skills; HR, human resources; SNCU, sick newborn care unit; LBW, low birth weight; OPV, oral polio vaccine.

### Baseline Assessment

From January to February 2016, a baseline assessment was conducted across all intervention facilities. The assessment included 748 data parameters on the labor room environment, staff competencies, and practices. While the labor room environment was assessed through a structured checklist based on MOHFW’s maternal and newborn health toolkit, competency assessment was conducted using a mix of vignette, knowledge, and simulation-based tools. Of the 1,410 staff nurses and auxiliary nurse-midwives (ANMs) posted at the intervention facilities during the assessment, 427 providers, identified based on 8-hourly duty shifts, were included in the assessment. Practices for a set of predetermined indicators at the intervention facilities were reviewed through facility-level labor room registers and case sheets with October–December 2015 serving as the reference period. While the labor room records were reviewed fully, case sheet-based records were assessed through a random sample of case sheets with 10 sheets for each of the 3 months at district hospitals and 5 at community health centers and other facilities (Supplement 1). The results of the baseline assessment were subsequently collated and analyzed using an Excel-based tool.

### Development of Intervention Package and Management Information System

Using the baseline assessment findings, the project team developed technical packages for the intervention. The 14 interventions were split into 2 groups: 1 set of interventions was required for all deliveries, and another set of interventions was used to build competencies for managing maternal and newborn complications. In 2016, the trainings aimed at improving competencies for monitoring the progress of labor, active management of the third stage of labor, essential newborn care, newborn resuscitation, and postnatal monitoring of mothers and newborns. The remainder of the interventions were rolled out in 2017. Similarly, establishing a management information system (MIS) involved identifying a set of standardized indicators to track the progress of the intervention through an Excel-based data entry tool.

### Implementation

The implementation was staggered with the first set of interventions rolled out through a series of district and on-site low-density, high-frequency training sessions between March and May 2016. Post-training monthly mentoring visits by district-level project officers were initiated from June 2016 to provide on-job handholding and supportive supervision. Facility QI platforms were established beginning October 2016. Similarly, trainings for the second set of interventions were conducted between February and April 2017.

The monthly MIS capturing key indicators was introduced at the facilities with efforts initially focused on streamlining data capture at facilities followed by ensuring regularity and completeness in data maintenance (Supplement 2).

### Endline Assessment

An independent external evaluation of the first set of interventions in the “Care Around Birth” approach was conducted by the Center for Operations Research and Training between November 2017 and February 2018. The assessment was conducted across a sample of 51 intervention facilities (the sample size was calculated using WHO’s Service Availability and Readiness Assessment’ technique) and included facility assessment; competency assessment of staff nurses/ANMs; in-depth interviews with staff nurses/ANMs, medical officers, district and state officials, and beneficiaries; and direct observation, of deliveries. Overall competency assessments and in-depth interviews were conducted with 195 staff nurses/ANMs, 77 medical officers, and 33 district and 8 state officials. Direct observation of 399 deliveries was conducted to determine essential practices, and 392 beneficiaries were interviewed to assess their experience with care and provision of services. Weights were then applied to the sample to generalize the findings to the intervention universe of 139 facilities, as 2 facilities (the primary health centers) had closed during the intervention period. Quantitative data were collated on CSPro and analyzed on SPSS, while the qualitative data were audio-recorded, transcribed, and coded thematically (Supplement 3).

### Data Management and Analysis

Data for the current paper have been analyzed at several levels: (1) labor room amenities and competency scores during the baseline assessment and external evaluation; (2) trends in essential maternal and newborn practices during the intervention period cumulatively and with facility and state-level differentials; and (3) provision and experience of care during the external evaluation. While aggregate level alterations over time were undertaken from the cumulative data of the 6 states, monthly records at each facility level were used to distinguish the variation in improvement over time. To compare the difference in the performance of the indicators at baseline and different phases of the intervention, Chi-square tests were performed. Each phase of the intervention was compared with its previous phase to examine whether the change in percent observed was significant or not. To compare the performance among levels of the facility and states scores were calculated for the pre and during intervention periods for each indicator. As the distribution of observations was skewed during both periods, median values were calculated to provide a better representation of the shift in improvement. Furthermore, provision and experience of care were estimated in percentage improvement.

### Ethical Clearance

As the model included facilitating the on-the-ground implementation of interventions included in the national service delivery package for maternal and newborn health, ethical clearance was not sought before implementation. However, Institutional Review Board clearance was obtained for conducting the endline assessment (No.EC –CORT/1730), and each respondent involved in the in-depth interviews provided their oral or written consent.

## RESULTS

We present the results from the first set of interventions.

### Labor Room Environment

The labor room environment was assessed across a set of indicators during the baseline and endline assessments, which included the availability of amenities, recording formats and protocols, infection control practices, and biomedical waste management. Improvement was observed across almost all of the indicators during the intervention (Table 3).

**TABLE 3.** Labor Room Environment in Health Facilities Included in Care Around Birth Approach Intervention in 6 States in India

**Indicator**	**Baseline (n=141)**	**External Assessment** **(n=139)**
**Amenities available in labor room**		
Attached functional toilet facility in labor room	74.5	79.9
LR has 24x7 running water facility	90.8	100.0
LR has 24x7 Electricity supply with functional power backup that includes an inverter or generator	80.1	96.3
A functional refrigerator	60.3	90.9
**Availability of equipment/furnishing**		
Mackintosh with each labor table	70.2	93.9
Functional Kelly’s pad on each labor table	41.1	78.9
Modular light for conducting deliveries	48.2	57.1
Functional wall clock with second hand/digital clock in labor room	90.1	97.9
Wall-mounted thermometer for measuring room temperature	61.0	85.9
Functional hemoglobinometer with reagents and lancet	34.8	70.5
Functional suction apparatus in labor room	35.5	83.8
Functional oxygen cylinder in labor room or central oxygen supply	75.2	92.4
Functional pulse oximeter in labor room	16.3	63.7
Newborn resuscitation bag	81.6	97.8
Pediatric stethoscope	22.7	67.8
Baby weighing scale	95.0	100.0
Radiant warmer	89.4	100.0
Radiant warmers have dedicated stabilizers	9.9	62.9
**Type of trays available**		
Delivery tray	66.0	98.0
Episiotomy tray	39.7	91.1
Baby tray or newborn tray	55.3	98.0
Medicine tray	51.1	97.1
Emergency drug tray	61.7	100.0
Postpartum intrauterine device tray	56.0	94.4
**Display of protocols**		
Active management of third stage of labor	64.5	94.8
Antepartum hemorrhage before 20 weeks	42.6	89.7
Antepartum hemorrhage after 20 weeks	41.8	93.8
Management of postpartum hemorrhage	67.4	98.6
Eclampsia	56.0	98.6
Breastfeeding	39.7	78.6
Kangaroo mother care	33.3	72.7
Newborn resuscitation	63.8	98.6
Handwashing	63.8	94.6
Preparation of 1 liter bleaching solution	40.4	94.8
Infection prevention	46.1	88.1
**Availability of recording formats**		
Referral register (out)	85.8	96.5
Maternal death record register	31.9	74.6
LR sterilization register	19.1	62.6
Handing over/taking over register	37.6	86.2
Discharge register	42.6	85.3
Postpartum intrauterine device/family planning register	72.3	96.4
Postnatal care register	16.3	76.5
**Hand hygiene and antisepsis**		
Handwashing facility at point-of-use (sink with running water)	94.3	98.0
Elbow operated taps	46.1	87.8
**Material for personal protection**		
Masks	75.9	98.0
Sterile gloves	95.7	100.0
Gowns/aprons	84.4	97.9
Shoe covers	33.3	70.4
Caps	59.6	86.1
Utility gloves and gum boots for housekeeping staff	23.4	84.8
Personal protective kit for delivering HIV-positive patients	35.5	57.2
**Environment control of patient care areas**		
Staff is trained to prepare cleaning solution (0.5% chlorine solution) as per standard procedure	73.8	100.0
External footwear is restricted	42.6	87.1
**Biomedical waste management**		
Color-coded bins at point of waste generation	88.7	98.0
Functional needle cutters	94.3	100.0
Puncture-proof box	57.4	92.3
Disinfection of sharp objects before disposal	56.0	100.0
Instruments dipped in 0.5% chlorine solution immediately after use	76.6	100.0

### Service Provider Competency

While most health care providers scored less than 50% for all the components during the baseline, after the intervention, competency improved across all of the components with maximum improvement observed for the use of the partograph, active management of the third stage of labor, essential newborn care, resuscitation, and newborn vaccination (Table 4).

**TABLE 4.** Service Provider Competency in Health Facilities Included in Care Around Birth Approach Intervention in 6 States in India

**Indicator**	**Baseline, % (n=424)**			**External assessment (n=1,176)**		
	**<50%**	**51%–80%**	**80%+**	**<50%**	**51%–80%**	**80%+**
Use of partograph	65	22	13	7	59	34
Active management of third stage of labor	15	52	33	8	31	62
Essential newborn care	66	26	7	2	28	70
Newborn resuscitation	68	27	5	1	17	82
Newborn vaccination	23	11	66	6	1	93
Postnatal monitoring	41	51	8	30	38	32
Infection prevention	40	54	6	18	39	43

Health care provider competency improved across all of the components.

*The good aspect of this project was skill enhancement. Skills have been enhanced which will stay with us. Staff who have been trained and posted at facilities will remain there and will train the new staff*. —Former Chief Medical Health Officer, Mandi, Himachal Pradesh

### Labor Room Practices

Practices in the labor rooms were monitored through on-site mentoring visits and collection of monthly maternal and newborn health-related datasets. These include the following:

- Maternal care during delivery: oxytocin administered within 1 minute of delivery, filled partograph, postpartum monitoring within 6 hours and at discharge, and average number of times mothers monitored during their stay at the facility.
- Essential newborn care and resuscitation: temperature recorded at birth, breastfeeding initiated within 1 hour of birth, delayed cord clamp, administration of vitamin K1, monitoring within 1 hour and within 6 hours, average number of times monitored after birth, and rates for birth dose vaccination.

Changes across these indicators were analyzed across 5 time periods: Period 1: October–December 2015 (baseline), Period 2: January–May 2016 (the baseline data collection and training period), Period 3: June–September 2016 (post-training mentorship period), Period 4: October–December 2016 (post-initiation of QI efforts by establishing QI teams and continued mentorship), Period 5: January–September 2017 (implementation with on-site mentoring and concurrent QI efforts) and Period 6: October–December 2017 (implementation and endline assessment).

**FIGURE 3:**
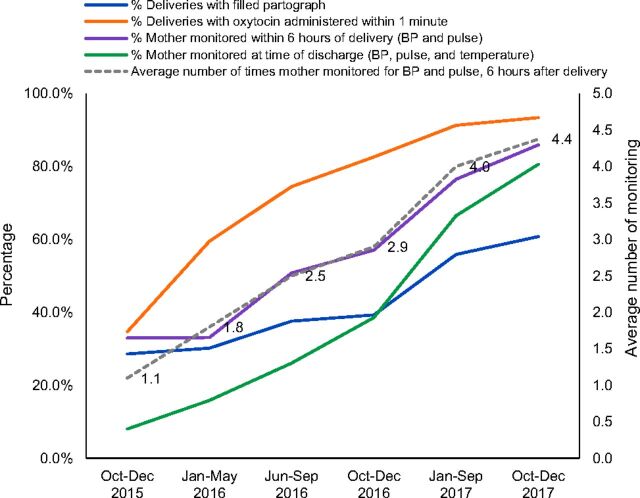
Improvement in Maternal Care During Delivery During Intervention to Improve Quality of Care in 6 States in India^a^ Abbreviation: BP, blood pressure. ^a^Dotted lines are represented on right side Y-axis due to different unit scale.

**FIGURE 4:**
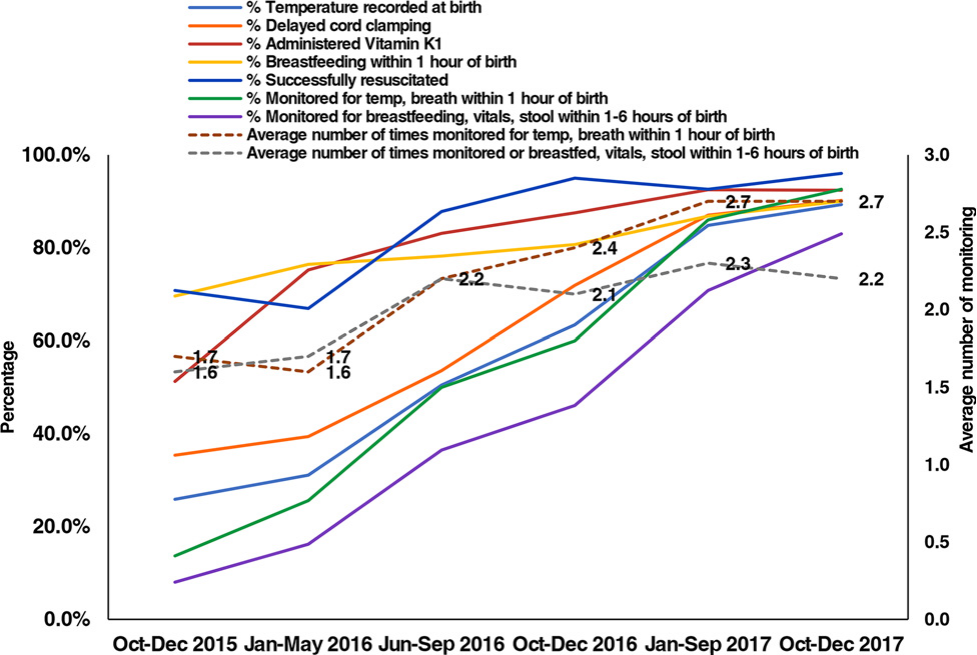
Improvement in Essential Newborn Care and Resuscitation During Intervention to Improve Quality of Care in 6 States in India

**FIGURE 5:**
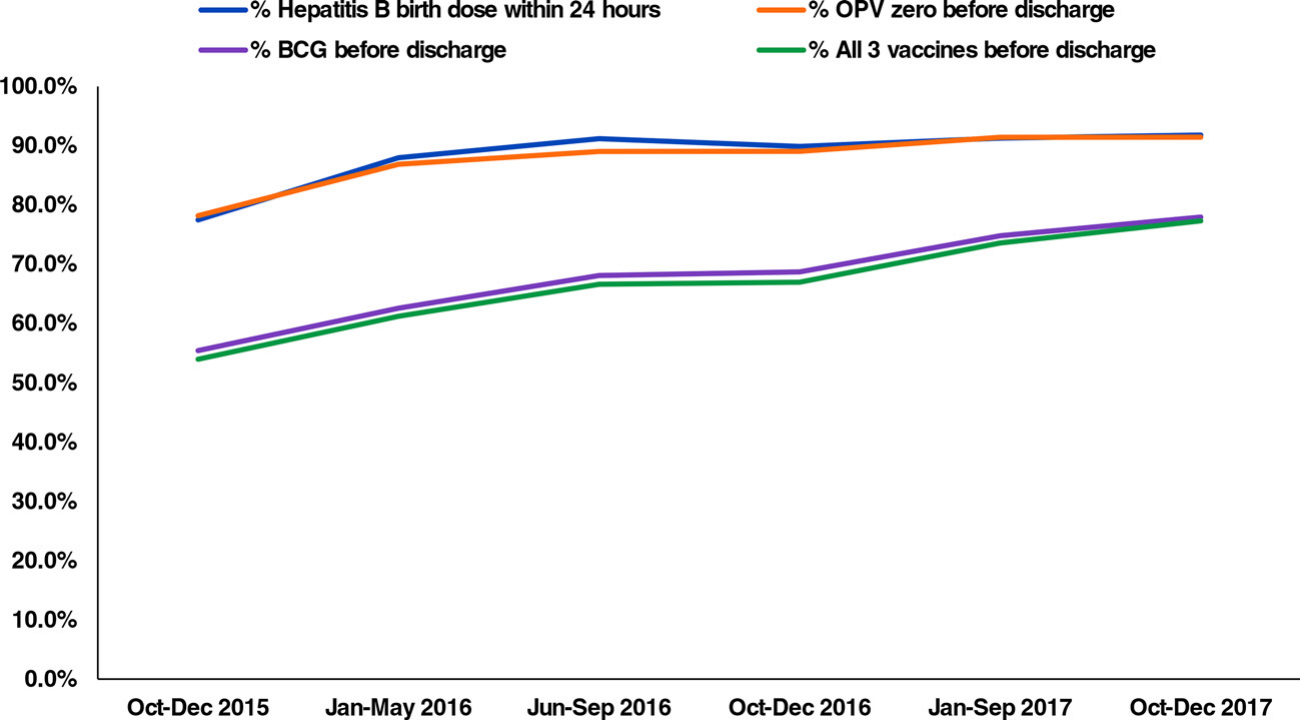
Improvement in Newborn Vaccination During Intervention to Improve Quality of Care in 6 States in India Abbreviation: OPV, oral polio vaccine.

An upward trend was seen across all maternal and newborn indicators over the intervention period indicating improvement in care practices (Figures 3, 4, 5 and Table 5 and Table 6). Statistically significant differences were seen across the intervention phases. For deliveries with filled partograph, it is seen that while period 2 did not differ much from period 1 which is the base period, period 3 when post-training mentorship was introduced marked a significant increase in partograph use (Table 5). Again, it was during period 5 with QI and on-site mentoring that the indicator saw a significant increase after which it did not change much for the subsequent period 6. Likewise, taking delayed cord clamping as another example, a significant difference in the practice was observed during periods 3, 4, and 5 after which the increase was not significant at period 6 (Table 6). It is evident that while performance for most of the indicators improved after training, the maximum impact was observed after mentorship, establishment of QI teams, and organization of QI meetings.

**TABLE 5.** Maternal Care During Delivery in Health Facilities Included in Care Around Birth Approach Intervention in 6 States in India

**Indicators** ^a^	**October-December 2015**	**January-May 2016**	**June-September 2016**	**October-December 2016**	**January-September 2017**	**October-December 2017**
Deliveries with filled partograph, %	28.6	30.2	37.6^c^	39.3	55.9^c^	60.8
Deliveries with oxytocin administered within 1 minute, %	34.7	59.5^c^	74.5^c^	82.6^c^	91.3^c^	93.4
Mothers monitored within 6 hours of delivery (BP and pulse), %	33.0	33.1	50.8^c^	57.1^b^	76.5^c^	85.9^b^
Mothers monitored at the time of discharge (BP, pulse, and temperature), %	8.0	15.9^b^	26.1^c^	38.6^c^	66.5^c^	80.6^c^
Average number of times mother monitored for BP and pulse; 6 hours after delivery	1.1	1.8^b^	2.5^c^	2.9^b^	4.0^c^	4.4

Abbreviation: BP, blood pressure.

a t-test for difference in percentage between subsequent phases.

^b^ P<.01.

^c^ P<.001.

**TABLE 6.** Essential Newborn Care, Resuscitation and Newborn Vaccination in Health Facilities Included in Care Around Birth Approach Intervention in 6 States in India

**Indicators** ^a^	**October-December 2015**	**January-May 2016**	**June-September 2016**	**October-December 2016**	**January-September 2017**	**October-December 2017**
Temperature recorded at birth, %	25.9	31.1	50.5^c^	63.4^c^	84.8^c^	89.3
Delayed cord clamping, %	35.4	39.4	53.6^c^	71.9^c^	87.0^c^	90.2
Administered Vitamin K1, %	51.3	75.2^b^	83.1^b^	87.5	92.5^b^	92.4
Breastfeeding within 1 hour of birth, %	69.6	76.4^b^	78.2	80.7	86.8^b^	90.0
Successfully resuscitated, %	70.8	66.9	87.8^c^	95.0^c^	92.6	96.0
Monitored for temperature, breathing within 1 hour of birth, %	13.7	25.6^b^	50.0^c^	60.0^c^	86.0^c^	92.6^b^
Monitored for breastfeeding, vitals, stool within 1–6 hours of birth, %	8.0	16.2^b^	36.5^c^	46.1^c^	70.8^c^	83.0^c^
Average number of times monitored for temp, breath within 1 hour of birth	1.7	1.6	2.2^c^	2.4^c^	2.7^c^	2.7
Average number of times monitored for breastfeeding, vitals, stool within 1–6 hours of birth	1.6	1.7^b^	2.2^c^	2.1	2.3^b^	2.2
Administered hepatitis B vaccine birth dose within 24 hours, %	77.5	88.0^b^	91.2	89.9	91.3	91.8
Administered OPV 0 before discharge, %	78.2	86.8^b^	89.0	89.0	91.4	91.4
Administered BCG before discharge, %	55.4	62.5^b^	68.1	68.7	74.8^b^	77.9
Administered all the three vaccines before discharge, %	53.9	61.2^b^	66.6	66.9	73.6^b^	77.3

Abbreviations: BP, blood pressure; OPV, oral polio vaccine.

^a^ t-test for difference in percentage between subsequent phases.

^b^ *P*<.01.

^c^ *P*<.001.

Moreover, apart from a few indicators like resuscitation and newborn vitals monitoring that had fluctuations during the intervention period, other indicators had an improved trajectory all through the intervention period.

Comparison of care practices of facility levels reveals that while district hospitals had satisfactory performance of certain indicators like oxytocin administration, vitals monitoring, vitamin K1 administration, breastfeeding and resuscitation at base period, first referral unit community health centers, non-first referral unit community health centers, 24x7 primary health centers had negligible practice of oxytocin administration or resuscitation and therefore, improvements seen in the indicators among these facilities were comparatively higher. There was hardly any practice of temperature recorded at birth or delayed cord clamping in most facilities but after the intervention, these practices rose above 90% (Table 7). Delhi, Jharkhand, and Uttarakhand states, which had very low levels of practice for most maternal and newborn indicators at baseline, made substantial improvement during the intervention period (Table 8). Comparatively, Haryana had started with fairly higher levels of practice of partograph usage, vitals monitoring, delayed cord clamping, temperature recorded at birth, and successful resuscitation. Hence, the difference in improvement for these indicators is marginal.

**TABLE 7.** Facility-Level Comparative Assessment of Maternal and Newborn Indicators Before and During Care Around Birth Approach Intervention in 6 States in India

**Indicators** ^a^	**Pre-intervention Phase (October 2015-May 2016)**					**Intervention Phase (June 2016-December 2017)**				
	**District Hospital** **(n=27)**	**Subdistrict Hospital/RH** **(n=25)**	**CHC-FRU** **(n=25)**	**CHC-non FRU** **(n=48)**	**24×7 PHC** **(n=14)**	**District Hospital (n=27)**	**Subdistrict Hospital/RH (n=25)**	**CHC-FRU** **(n=25)**	**CHC-non FRU** **(n=48)**	**24×7 PHC** **(n=14)**
**Maternal Care**										
Deliveries with filled partograph, %	13.0	27.0	2.0	26.0	21.0	43.5	68.5	67.0	81.0	88.5
Deliveries with oxytocin administered within one minute, %	63.5	0.0	0.0	62.5	0.0	97.0	98.0	99.0	98.0	100.0
Mother monitored within 6 hours of delivery (BP and pulse) , %	70.0	65.0	68.0	40.0	68.0	95.0	93.0	95.5	100.0	94.0
Mother monitored at the time of discharge (BP, pulse, and temperature), %	0.0	0.0	0.0	0.0	0.0	40.0	46.5	50.0	82.0	87.0
Average number of times mother monitored for BP and pulse, 6 hours post-delivery	0.55	0.40	0.45	0.40	0.35	2.25	3.35	2.65	3.50	4.20
**Essential Newborn Care and Resuscitation**										
Temperature recorded at birth, %	0.0	0.0	0.0	0.0	0.0	87.5	98.0	96.0	98.5	100.0
Delayed cord clamping, %	0.0	0.0	0.0	7.0	0.0	92.0	96.0	94.5	96.0	99.0
Administered Vitamin K1, %	95.0	99.0	85.0	96.5	82.5	98.5	99.0	99.0	99.0	98.0
Breastfeeding within 1 hour of birth, %	93.5	99.0	97.5	99.0	95.5	90.5	96.0	97.0	98.0	98.0
Successfully resuscitated, %	76.0	42.5	0.0	61.5	0.0	97.0	100.0	100.0	100.0	100.0
Monitored for temp, breath within 1 hour of birth, %	0.0	0.0	0.0	0.0	0.0	82.0	84.0	93.0	98.0	93.0
Monitored for breastfeeding, vitals, stool within 1–6 hours of birth, %	0.0	0.0	0.0	0.0	0.0	58.0	70.0	67.0	87.0	66.0
Monitored for temperature, breathing, and feeding at discharge, %	0.0	0.0	0.0	0.0	0.0	30.0	41.0	47.0	73.0	60.0
Average number of times monitored for temperature, breathing within 1 hour of birth	0	0	0	0	0	1.2	2	1.9	2.4	2.25
Average number of times monitored for breastfeeding, vitals, stool within 1–6 hours of birth	0	0	0	0	0	1	1.3	1.4	1.1	1
**Newborn Vaccination**										
Administered hepatitis B vaccine birth dose within 24 hours, %	93.0	98.0	97.0	96.5	97.5	95.0	97.0	97.0	98.0	98.0
Administered OPV zero before discharge, %	94.0	99.0	96.0	96.0	98.0	95.0	97.5	96.5	97.0	98.0
Administered BCG before discharge, %	87.5	72.0	45.0	85.5	22.5	94.0	81.0	79.0	91.0	80.0
Administered all the 3 vaccines before discharge, %	86.0	68.0	45.0	84.0	22.5	92.0	79.0	77.5	88.0	78.0

Abbreviations: 24×7 PHC, primary health center operating 24 hours a day/7 days a week; BP, blood pressure; CHC-FRU, first referral unit community health care center; CHC-non-FRU, non-first referral unit-community health care center; OPV, oral polio vaccine; RH, referral hospital.

^a^ Data for 2 HSCs has not been included in the analysis.

**TABLE 8.** Interstate Comparative Assessment of Maternal and Newborn Indicators Before and During Care Around Birth Approach Intervention in 6 States in India

**Indicator** ^a^	**Pre-Intervention Phase (October 2015-May 2016)**						**Intervention Phase (June 2016-December 2017)**					
	**Delhi (n=8)**	**Haryana (n=32)**	**Himachal Pradesh (n=16)**	**Jharkhand (n=42)**	**Punjab (n=18)**	**Uttarakhand (n=25)**	**Delhi (n=8)**	**Haryana (n-32)**	**Himachal Pradesh (n=14)**	**Jharkhand (n-42)**	**Punjab (n=18)**	**Uttarakhand (n=25)**
**Maternal Care**												
Deliveries with filled partograph, %	0.0	90.0	64.5	15.5	0.0	0.0	71.0	96.0	86.5	56.5	44.0	59.0
Deliveries with oxytocin administered within 1 minute, %	9.0	91.0	0.0	44.0	0.0	17.5	100.0	99.0	100.0	97.0	99.0	99.0
Mothers monitored within 6 hours of delivery (BP and pulse), %	100.0	91.0	60.0	0.0	80.0	0.0	100.0	100.0	95.0	100.0	90.0	83.0
Mothers monitored at the time of discharge (BP, pulse, and temperature), %	0.0	0.0	0.0	0.0	0.0	0.0	100.0	69.5	58.0	80.0	23.0	41.5
Average number of times mother monitored for BP and pulse; 6 hours after delivery	1.85	1.65	0.50	0.00	0.45	0.00	3.50	5.35	3.10	2.30	2.70	2.40
**Essential Newborn Care and Resuscitation**												
Temperature recorded at birth, %	0.0	100.0	100.0	0.0	0.0	0.0	88.5	100.0	100.0	92.5	87.0	82.5
Delayed cord clamping, %	0.0	100.0	100.0	0.0	0.0	0.0	90.5	99.0	100.0	92.0	88.0	93.0
Administered Vitamin K1, %	67.5	92.5	100.0	96.5	62.0	73.0	96.0	95.5	100.0	99.0	99.0	99.0
Breastfeeding within 1 hour of birth, %	6.5	98.5	100.0	98.0	98.0	0.0	83.5	97.5	100.0	97.5	91.5	91.0
Successfully resuscitated, %	0.0	85.5	0.0	96.0	0.0	0.0	100.0	100.0	88.0	98.5	92.0	88.0
Monitored for temp, breath within 1 hour of birth, %	100.0	9.0	0.0	0.0	0.0	0.0	100.0	93.0	91.0	93.0	76.0	93.0
Monitored for breastfeeding, vitals, stool within 1–6 hours of birth, %	100.0	0.0	0.0	0.0	0.0	0.0	100.0	71.0	73.0	76.0	51.5	48.0
Monitored for temperature, breathing and feeding at discharge, %	0.0	0.0	0.0	0.0	0.0	0.0	93.0	50.0	57.5	74.0	21.0	39.5
Average number of times monitored for temp, breath within 1 hour of birth	0.3	0.2	0	0	0	0	1.3	2.2	2.3	2.1	1.45	2.45
Average number of times monitored or breastfeeding, vitals, stool within 1-6 hours of birth	0.3	0	0	0	0	0	1.5	1	1	1	1.45	1.3
**Newborn Vaccination**												
Administered hepatitis B vaccine birth dose within 24 hours, %	95.5	97.0	98.0	94.0	97.0	75.0	95.0	97.0	99.0	97.0	98.0	94.0
Administered OPV 0 before discharge, %	99.0	97.0	100.0	94.0	96.0	76.5	95.0	97.0	99.0	96.0	98.0	95.5
Administered BCG before discharge, %	34.0	91.0	95.5	84.0	20.0	0.0	93.0	95.0	97.0	86.0	77.0	19.5
Administered all the 3 vaccines before discharge, %	34.0	90.0	89.0	81.0	10.0	0.0	92.0	93.0	96.0	83.5	77.0	21.5

Abbreviations: BP, blood pressure; OPV, oral polio vaccine.

^a^ Data for 2 HSCs in Himachal Pradesh has not been included in the analysis for the intervention phase.

### Endline Assessment Results

#### Provision of Care

While monitoring the progress of labor using a partograph was done in 75% of deliveries observed during the endline assessment, 100% of women were administered oxytocin after birth with 99% receiving it within 1 minute of delivery. After delivery, newborns were kept in skin-to-skin contact with mothers in 86% of observations; breastfeeding was initiated within 1 hour of birth in 96% of cases; injection of vitamin K1 was administered to 97% of newborns; 99% of newborns were weighed at birth; and temperature was recorded for 88% of newborns observed. Health care providers used sterile disposable gloves during all the deliveries that were observed, 85% of health care providers practiced handwashing before examining the newborns, and protocols for safe disposal of placenta were followed in 99% of observations. Privacy was maintained in 84% of observations, and birth companions were allowed inside labor rooms in 78% of cases.

#### Experience of Care

Almost all the respondents reported the delivery room to be clean, and 84% reported the toilets to be clean. A majority of respondents (93%) reported availability of running water in the facility, 81% reported drinking water to be available; and 72% received free meals at the facilities. Regarding breastfeeding, 86% had initiated breastfeeding within the first hour after birth and 81% reported receiving necessary support for initiating breastfeeding. A majority (87%) reported that the nurse and/or doctor had checked them and/or the newborn during the postnatal period. Clients also reported that they had received information/counseling from providers, which included information on postpartum family planning (51%), hygiene and handwashing (44%), and the continuation of exclusive breastfeeding (41%) for the first 6 months. Clients were also provided with counseling information that applied to mothers, such as eating regular meals (23%) and drinking fluids regularly (18%), and to newborns, such as not applying anything to the cord stump (17%) and monitoring for danger signs. Nearly half (49%) of clients stated that they were able to ask health care providers questions about their own and their newborn’s health when needed. Regarding services provided at the facilities, 67% of clients were “satisfied” and 31% were “very satisfied,” with 97% of clients stating that the health care providers were supportive.

*I am very happy with the services here and the nurses talk very nicely to me. They also explain everything and make me feel comfortable.* —Client, district hospital, Muktsar, Punjab

Although a preliminary analysis of facility data indicated a reduction in perinatal mortality, the duration of the intervention limited any definitive statistical analysis.

## DISCUSSION

With the global transition to the Sustainable Development Goals framework, multiple efforts have been undertaken for improving and optimizing the quality of care across the health care delivery system.^1^^,^^22^^,^^26^^–^^29^

The Care Around Birth approach adds to the existing body of evidence by potentiating efforts to improve the quality of health care delivery systems using a training, mentoring, and QI model in resource-constrained and poor-performing districts in India.

The Care Around Birth approach adds to the existing body of evidence by potentiating efforts to improve the quality of health care delivery systems using a training, mentoring, and QI model in resource-constrained and poor-performing districts in India.

In a relatively short period of 2 years, this approach has been able to demonstrate improvements. Possible factors include addressing and streamlining implementation challenges through an inclusive problem-solving approach with attention to detail, comprehensive planning for end-to-end solutions, clear messaging, and role clarity. The approach ensured a high level of engagement from the government counterparts at all levels that generated ownership, which is in line with available literature.^29^ Implementing the approach demonstrated that optimizing the use of existing resources for driving and sustaining change with continued advocacy for long-term goals, including improvement in infrastructure and human resources, should be the way forward for any health intervention. Further, the fact that the approach was implemented in 26 districts across 6 states with variable resources and levels of service delivery enabled the project team to learn and gain insights into the nuances of implementation, understand the need for improvisation for improvement, and ensure that mechanisms were built-in to sustain and scale complex and integrated interventions.^30^^–^^31^

By designing the training package according to the sequence of events in the labor room and postnatal wards, the approach integrated maternal and newborn care and helped health care providers recognize the importance of managing both the mother and the newborn together as 1 unit and ensured competencies for both maternal and neonatal care. The integration of interventions and operational strategies in the approach is in line with recent evidence that suggests while single interventions are unlikely to achieve reductions in maternal, newborn, and child mortality, combining interventions is more effective in improving health care practices.^29^^,^^32^

By designing the training package according to the sequence of events in the labor room and postnatal wards, the approach helped health care providers recognize the importance of managing both the mother and the newborn together as 1 unit.

The approach adopted a staggered implementation framework where different capacity-building measures were initiated and implemented over 2 years. While the baseline assessment helped provide a thorough understanding of the intervention facilities, the initial round of short, centralized, and facility-based low-density, high-frequency trainings not only refreshed provider competency but also helped saturate facilities with trained health care providers thereby negating the potential impact of variable service provider competencies. The mentorship visits immediately after the trainings helped sustain the gains, improved the availability of equipment and logistics at the facilities, and engendered trust and mutual respect between the project staff and health care providers at the intervention facilities. The subsequent introduction of QI approaches through facility teams then transferred ownership to health care providers, which ensured accountability and local problem solving. It was also evident that this staggered implementation not only built the capacity of the health system to absorb and inculcate change but also ensured facility readiness to adopt QI approaches, which usually tend to work better in areas with increased levels of resources than in low-resource settings.^30^

Data-driven decision making and facility-based QI teams formed the fulcrum of the approach. While robust measurement, collation, and triangulation of data enabled the intervention facilities to adopt a comprehensive approach to improvement, the QI efforts utilized this data to address issues at the facility level. The dual approach of combining quantitative data through the MIS and qualitative data through the QI meetings demystified various implementation challenges and issues. The approach also differed from the traditional plan-do-study-act approach by using predefined data elements across a set of indicators to foster improvements rather than addressing 1 improvement aim at a time. This possibly helped faster improvement across the facilities and countered the narrative of QI approaches failing due to lack of leadership, financial, organizational, and system support.^33^ However, the success of the QI approaches was differential, with some interventions demonstrating preferable results compared to others.

The approach differed from the traditional plan-do-study-act approach by using predefined data elements across a set of indicators to foster improvements rather than addressing 1 improvement aim at a time.

Though overall improvements were noted inter-state and inter-facility, variations in results were observed during the implementation. These variations are attributable to the overall performance of health systems in the states and point toward the need for comprehensive improvements at the systems level for sustained gains.

The approach prioritized high caseload facilities, however, engagement of the L1 facilities through a pared-down version of basic obstetric and newborn care could have led to improvements in quality of care across all levels, as well as possibly a more optimal distribution of caseload. Similarly, the interventions targeting maternal and newborn complication management including management of postpartum hemorrhage and pre-eclampsia/eclampsia, kangaroo mother care, assisted feeding of low birth weight babies, use of antenatal corticosteroids for preterm labor, management of maternal and newborn sepsis, and the prevention of parent to child transmission were implemented for less than a year and did not provide enough time for measurement and evaluation, moreover the relatively short implementation period might have limited sustainability of efforts. The approach also did not strengthen referral pathways.

Although the approach did not measure motivation among staff, it can be presumed that improving the experience of care for beneficiaries is directly proportional to not only the competency but also the motivation level of health care providers. As there was hardly any increase in staff strength at the intervention facilities during the implementation period, improved results are a marker of both better competency and accountability of the facility staff. Hence, all interventions should identify health care providers as important stakeholders and ensure sustained capacity building through a multipronged approach to build competencies and enhance motivation and self-confidence among them. Also, while most of the training packages focus on improving clinical competencies, health care providers should acquire skills in time management, team building, and interpersonal communication. These skills are essential to health care providers, especially those in high-caseload facilities with low human resource capacity. Though the approach had these aspects incorporated in the clinical training modules, soft skills trainings should be designed and implemented for health care providers.^34^

It is increasingly recognized that all women need and deserve respectful care and that RMC should be promoted as a critical element of strategies to improve the utilization and quality of maternity care.^35^^–^^36^ The approach aimed to improve RMC by integrating it into overall health system strengthening efforts. Contrary to multiple studies, which point to care being compromised due to mistreatment and disrespectful care during childbirth,^37^^–^^38^ the current intervention reported good levels of satisfaction among clients. While this may be due to low levels of expectation, integrating RMC into the overall efforts to improve the labor room environment, competencies, and service delivery mechanisms and targeting health care providers to incorporate respectful care practices into their scope of work might well be responsible for the better experience of care by beneficiaries. This is in line with recent evidence that shows that improving the quality of care by promoting RMC needs to not only address interactions between the woman and the provider but also make improvements at the health-systems level. Thus, RMC should not be considered as an isolated intervention but rather as a critical component for providing good-quality care for mothers and newborns within health systems.^39^

Sustained advocacy at the state level helped to mobilize financial resources to address larger systemic issues through annual program implementation plans with states providing funds for labor room strengthening, setting up skill labs, and training health care providers. In addition, efforts were made to build ownership by ensuring that government staff and program officers were identified to serve as focal points for reviewing progress and addressing challenges at the state, district, and facility levels and developing a pool of state-level trainers to ensure continued scale up to other facilities and districts across the states. Moreover, the partnership with the state governments helped to sustain and ensure a contextualized scale up of the approach. Some states like Punjab and Delhi scaled up the initiative statewide, but others undertook both intra- and inter-district scale-ups. Learnings from the approach, specifically those related to facility-level QI initiatives and RMC, were then integrated into the national labor room and maternity operation theater QI initiative, LaQshya, with the project supporting a national program management unit based out of the MOHFW for program rollout and implementation across the country and focused support to LaQshya implementation across 7 states through dedicated state units. A substantial number of the Care Around Birth facilities were also designated as LaQshya facilities and received state and national certifications in due course.

### Limitations

While the “Care Around Birth” approach demonstrated impact at scale, it did have certain limitations. The difference in the methodology for the baseline assessment and external evaluation might have a bearing on the overall impact, moreover, the absence of control districts did not allow for comparative assessment. Both the provision and experience of care were measured only during the endline and hence results need to be interpreted cautiously as there might have been a Hawthorne effect.^40^ In addition, though the approach presumably demonstrated a decline in mortality rates these aspects need to be further studied.

## CONCLUSION

Overall, the Care Around Birth approach has demonstrated impact at scale. It is envisaged that subsequent efforts in this field will gain insights from the approach to comprehensively address key drivers of maternal and newborn mortality.

## Supplementary Material

20-00368-Sarin-Supplement3.pdf

20-00368-Sarin-Supplement1.pdf

20-00368-Sarin-Supplement2.pdf

## Notes

**Peer Reviewed**

**First published online:** August 13, 2021.

## References

[1] Kruk ME Gage AD Arsenault C. High-quality health systems in the Sustainable Development Goals era: time for a revolution. Lancet Glob Health. 2018. 6(11):e1196–e1252. 10.1016/s2214-109x(18)30386-3. 30196093 PMC7734391

[2] Kieny M-P Evans TG Scarpetta S. *Delivering Quality Health Services: A Global Imperative for Universal Health Coverage*. World Bank; 2018. Accessed July 7, 2021. https://documents.worldbank.org/en/publication/documents-reports/documentdetail/482771530290792652/delivering-quality-health-services-a-global-imperative-for-universal-health-coverage

[3] National Academies of Sciences, Engineering, and Medicine. *Crossing the Global Quality Chasm: Improving Health Care Worldwide*. The National Academies Press; 2018. Accessed July 7, 2021. 10.17226/25152 30605296

[4] World Health Organization (WHO). *World Health Statistics 2016: Monitoring Health for the Sustainable Development Goals*. WHO; 2016. Accessed July 7, 2021. https://apps.who.int/iris/handle/10665/206498

[5] Lawn JE Blencowe H Waiswa P. Stillbirths: rates, risk factors, and acceleration towards 2030. Lancet. 2016;387(10018):587–603. 10.1016/S0140-6736(15)00837-5. 26794078

[6] *The Global Strategy for Women’s Children’s and Adolescent’s Health (2016–2030)*. United Nations; 2015. Accessed July 7, 2021. https://www.who.int/life-course/partners/global-strategy/globalstrategyreport2016-2030-lowres.pdf?ua=1

[7] India has achieved groundbreaking success in reducing maternal mortality. World Health Organization website. June 10, 2018. Accessed July 7, 2021. https://www.who.int/southeastasia/news/detail/10-06-2018-india-has-achieved-groundbreaking-success-in-reducing-maternal-mortality

[8] Fadel SA Rasaily R Awasthi S; Million Death Study Collaborators. Changes in cause-specific neonatal and 1–59-month child mortality in India from 2000 to 2015: a nationally representative survey. Lancet. 2017;390(10106):1972–1980. 10.1016/S0140-6736(17)32162-1. 28939096 PMC5677556

[9] Nearly one thousand fewer women now die of pregnancy related complications each month in India. The Partnership for Maternal, Newborn & Child Health website. June 6, 2018. https://www.who.int/pmnch/media/news/2018/pregnancy-related-complications-india/en/

[10] India 12th worst among 52 lower middle-income nations for newborns, says Unicef report. Scroll. February 20, 2018. Accessed July 7, 2021. https://scroll.in/latest/869315/india-12th-worst-among-52-lower-middle-income-nations-for-newborns-says-unicef-report

[11] Powell-Jackson T Mazumdar S Mills A. Financial incentives in health: new evidence from India’s Janani Suraksha Yojana. J Health Econ. 2015;43:154–169. 10.1016/j.jhealeco.2015.07.001. 26302940

[12] Ng M Misra A Diwan V Agnani M Levin-Rector A De Costa A. An assessment of the impact of the JSY cash transfer program on maternal mortality reduction in Madhya Pradesh, India. Glob Health Action. 2014;7(1):24939. 10.3402/gha.v7.24939. 25476929 PMC4256523

[13] Government of India. Ministry of Health and Family Welfare (MOHFW). *Operational Guidelines for Quality Assurance in Public Health Facilities*. MOHFW; 2013. Accessed July 7, 2021. http://www.rrcnes.gov.in/quality%20Assurance/Operational%20Guidelines%20on%20Quality%20Assurance%20%28Print%29.pdf

[14] Government of India. Ministry of Health and Family Welfare (MOHFW). *Dakshata Empowering Providers for Improved MNH Care During Institutional Deliveries. A Strategic Initiative to Strengthen Quality of Intra- and Immediate Postpartum Care*. MOHFW; 2015. Accessed July 7, 2021. https://docplayer.net/45580840-A-strategic-initiative-to-strengthen-quality-of-intra-and-immediate-postpartum-care.html

[15] Government of India. Ministry of Health and Family Welfare (MOHFW). *Guidelines for Standardization of Labour Rooms at Delivery Points*. MOHFW; 2016. Accessed July 7, 2021. http://nhm.gov.in/images/pdf/programmes/maternal-health/guidelines/Labor_Room%20Guideline.pdf

[16] Government of India. Ministry of Health and Family Welfare (MOHFW). *LaQshya: Labour Room Quality Improvement Initiative*. MOHFW; 2017. Accessed July 7, 2021. http://nhm.gov.in/New_Updates_2018/NHM_Components/RMNCH_MH_Guidelines/LaQshya-Guidelines.pdf

[17] Taneja G Sridhar VSR Mohanty JS. India’s RMNCH+A Strategy: approach, learnings and limitations. BMJ Glob Health. 2019;4(3):e001162. 10.1136/bmjgh-2018-001162. 31139464 PMC6509590

[18] Tunçalp Ö Were WM MacLennan C. Quality of care for pregnant women and newborns—the WHO vision. BJOG. 2015;122(8):1045–1049. 10.1111/1471-0528.13451. 25929823 PMC5029576

[19] Center for Operations Research & Training. External Evaluation of Care Around Birth Approach Implemented by IPE Global in High Priority Districts of Six Focus States. August 2018.

[20] Leal-Rodríguez AL Albort-Morant G. Promoting innovative experiential learning practices to improve academic performance: empirical evidence from a Spanish Business School. J Innovation & Knowledge. 2019;4(2):97–103. 10.1016/j.jik.2017.12.001

[21] McLaughlin C. Mentoring: what is it? How do we do it and how do we get more of it? Health Serv Res. 2010;45(3):871–884. 10.1111/j.1475-6773.2010.01090.x. 20337731 PMC2875765

[22] Deorari A Livesley N. Delivering quality healthcare in India: beginning of improvement journey. Indian Pediatr. 2018;55(9):735–737. 10.1007/s13312-018-1370-9. 30345974

[23] Taylor MJ McNicholas C Nicolay C Darzi A Bell D Reed JE. Systematic review of the application of the plan–do–study–act method to improve quality in healthcare. BMJ Qual Saf. 2014;23(4):290–298. 10.1136/bmjqs-2013-001862. 24025320 PMC3963536

[24] United States Agency for International Development (USAID). CLA Toolkit. Accessed July 7, 2021. https://usaidlearninglab.org/qrg/understanding-cla-0

[25] Government of India. Ministry of Health and Family Welfare (MOHFW). *Maternal and Newborn Health Toolkit*. MOHFW; 2013. Accessed July 7, 2021. http://statehealthsocietybihar.org/rch/MNH%20Toolkit_23_11_2013.pdf

[26] Berwick D Snair M Nishtar S. Crossing the global health care quality chasm: a key component of universal health coverage. JAMA. 2018;320(13):1317–1318. 10.1001/jama.2018.13696. 30177995

[27] Syed SB Leatherman S Mensah-Abrampah N Neilson M Kelley E. Improving the quality of health care across the health system. Bull World Health Organ. 2018;96(12):799. 10.2471/BLT.18.226266. 30505024 PMC6249706

[28] Board on Global Health, Institute of Medicine, The National Academies of Sciences, Engineering, and Medicine. *Improving Quality of Care in Low- and Middle-Income Countries: Workshop Summary*. National Academies Press; 2015. Accessed July 7, 2021. 10.17226/21736 26677493

[29] Goyet S Broch-Alvarez V Becker C. Quality improvement in maternal and newborn healthcare: lessons from programmes supported by the German development organisation in Africa and Asia. BMJ Glob Health. 2019;4(5):e001562. 10.1136/bmjgh-2019-001562. 31565404 PMC6747907

[30] Rowe AK Rowe SY Peters DH Holloway KA Chalker J Ross-Degnan D. Effectiveness of strategies to improve health-care provider practices in low-income and middle-income countries: a systematic review. Lancet Glob Health. 2018;6(11):e1163–e1175. 10.1016/S2214-109X(18)30398-X. 30309799 PMC6185992

[31] Rowe AK Labadie G Jackson D Vivas-Torrealba C Simon J. Improving health worker performance: an ongoing challenge for meeting the sustainable development goals. BMJ. 2018;362:k2813. 10.1136/bmj.k2813. 30061366 PMC6283359

[32] Goldenberg RL McClure EM Saleem S. Improving pregnancy outcomes in low- and middle-income countries. Reprod Health. 2018;15(S1)(Suppl 1):88. 10.1186/s12978-018-0524-5. 29945628 PMC6019988

[33] Reed JE Card AJ. The problem with Plan-Do-Study-Act cycles. BMJ Qual Saf. 2016;25(3):147–152. 10.1136/bmjqs-2015-005076. 26700542 PMC4789701

[34] Bhutta ZA Salam RA Lassi ZS Austin A Langer A. Approaches to improve Quality of Care (QoC) for women and newborns: conclusions, evidence gaps and research priorities. Reprod Health. 2014;11(S2)(Suppl 2):S5. 10.1186/1742-4755-11-S2-S5. 25208572 PMC4160923

[35] Hill K Stanton ME. Promoting evidence and action for respectful care at birth, a presentation at the USAID mini-University at Georgetown University. 2010.

[36] Vogel JP Bohren MA Tunçalp Ö Oladapo OT Gülmezoglu AM. Promoting respect and preventing mistreatment during childbirth. BJOG. 2016;123(5):671–674. 10.1111/1471-0528.13750. 26628382 PMC5063112

[37] Bohren MA Hunter EC Munthe-Kaas HM Souza JP Vogel JP Gülmezoglu AM. Facilitators and barriers to facility-based delivery in low- and middle-income countries: a qualitative evidence synthesis. Reprod Health. 2014;11(1):71. 10.1186/1742-4755-11-71. 25238684 PMC4247708

[38] Bohren MA Vogel JP Hunter EC. The mistreatment of women during childbirth in health facilities globally: a mixed-methods systematic review. PLoS Med. 2015;12(6):e1001847, discussion e1001847. 10.1371/journal.pmed.1001847. 26126110 PMC4488322

[39] Shakibazadeh E Namadian M Bohren MA. Respectful care during childbirth in health facilities globally: a qualitative evidence synthesis. BJOG. 2018;125(8):932–942. 10.1111/1471-0528.15015. 29117644 PMC6033006

[40] McCambridge J Witton J Elbourne DR. Systematic review of the Hawthorne effect: New concepts are needed to study research participation effects. J Clin Epidemiol. 2014;67(3):267–277. 10.1016/j.jclinepi.2013.08.015. 24275499 PMC3969247

