# Supplementary material for: Care Around Birth Approach: A Training, Mentoring, and Quality Improvement Model to Optimize Intrapartum and Immediate Postpartum Quality of Care in India
Source: Glob Health Sci Pract. 2021 Sep 30;9(3):590–610. doi: 10.9745/GHSP-D-20-00368 (PMC8514027; doi:10.9745/GHSP-D-20-00368)
Supplement: 20-00368-Sarin-Supplement3.pdf [file 20-00368-Sarin-Supplement3.pdf]

External Evaluation of Care around Birth Approach Implemented by IPE Global in High Priority districts of Six Focus States

- A Report

Submitted to

IPE Global, New Delhi

Submitted by

CORT
CENTRE FOR OPERATIONS RESEARCH & TRAINING
research that makes a difference

Centre for Operations Research and Training

402, Woodland Apartments, Race Course, Vadodara, INDIA

August 2018

Content

Chapter 1: Introduction **1**
1.1 Background 1
1.2 Project VRIDDHI: Care Around Birth (Cab) Approach 1
1.3 External Assessment 2
1.3.1 Objective of the Assessment 2
1.3.2 Methodology 3
1.3.3 Study Universe 3
1.3.4 Target group 3
1.3.5 Study tools 3
1.3.6 Sampling Design 4
1.3.7 Sample Achievement 5
1.3.8 Interviews with stakeholders 5
1.3.9 Study Implementation 6
1.3.10 Challenges during Assessment 6
1.3.11 Data Management and Analysis 6

Chapter 2: Facility Level Assessment **8**
2.1 Facility Readiness 8
2.1.1 Infrastructure 8
2.1.2 Availability of Equipment 10
2.1.3 Availability of protocols and IEC display in labour room and postnatal ward 11
2.1.4 Documentation 12
2.1.5 Infection prevention Practices 13
2.1.6 Support/Ancillary services at Facility 14
2.2 Healthcare Providers in Labour Room 15
2.2.1 Profile of Labour Room staff 15
2.2.2 Capacity building under CaB approach 16
2.2.3 Competency of Labour Room staff 18
2.3 Quality Improvement (QI) teams 21
2.4 Stakeholders and Healthcare Provider's Perspective on Changes due to CaB Approach Implementation 21

Chapter 3: Quality of Services **24**
3.1 Service Delivery Observations 24
3.1.1 First stage of labour 24
3.1.2 Second stage of labour 24
3.1.3 Third stage of labour 25
3.1.4 Fourth stage of labour 25
3.2 Client Perspective 25
3.2.1 Profile of beneficiary 26
3.2.2 Service received 27

Chapter 4: Conclusion and Recommendation **30**
4.1 Readiness of Labour Room 30
4.2 Competency Level of Health Providers 30
4.3 Client Perspective on Quality of Service 31
4.4 Crosscutting Recommendations 31
Annexure 33

List of Tables

Table 1.1: Coverage of facilities and beneficiaries across the six states 5

Table 1.2: Number of service providers interviewed in selected facilities 5

Table 2.1: Amenities in labour room during baseline and External assessment 9

Table 2.2: Medical equipment in labour room during baseline and External assessment 9

Table 2.3: Status of designated trays in labour room during baseline and External assessment 10

Table 2.4: Status of delivery, medicine and PPIUCD trays during baseline and External assessment 10

Table 2.5: Display of protocols in labour room during baseline and External assessment 11

Table 2.6: Registers available and updated in facilities during baseline and External assessment 12

Table 2.7: Selected indicators from documented data of last five deliveries in register 13

Table 2.8: Material and management of Infection prevention during baseline and External assessment........ 13

Table 2.9: Support services at facility during baseline and External assessment 14

Table 2.10: Competancy score of healthcare providers in AMTSL during baseline and External assessment across level of facilities 18

Table 2.11: Competancy score of healthcare providers in AMTSL during baseline and External assessment across level of facilities 19

Table 2.12: Competancy score of healthcare providers in New Born Care during baseline and External assessment across level of facilities 19

Table 2. 13: Competancy score of healthcare providers in Postnatal Monitoring during baseline and External assessement across levels 20

Table 2.14: Competancy score of helath providers in infection prevention during baseline and External assessment across levels 20

Table 3.1: Profile of beneficiaries 26

Table 3.2: Services received on arrival 27

Table 3.3: Services received by the beneficiary........ 27

List of Figures

Figure 2.1: Percentage of healthcare providers participated in CaB training........ 16

Figure 3.1: Practice of Administring Oxytocin during baseline and External assessment 25

Figure 3.2: Person who accompanied beneficiary to facility 26

Chapter 1
Introduction

1.1 Background

The period during and immediately after childbirth remains critical for maternal and newborn survival and wellbeing. Majority of maternal deaths occur due to complications during pregnancy and childbirth like, haemorrhage, sepsis, and hypertensive disorders. Likewise some of the common reasons for neonatal deaths like preterm birth, birth asphyxia, intrapartum related neonatal death and neonatal infections, also occur during or around the period of pregnancy/childbirth. Most of these common causes of deaths are preventable in the presence of good healthcare facilities supplemented with quality clinical services.

Government of India (GoI) made a conscious endeavour to address the issues of maternal and child mortality and morbidity through the launch of National Rural Health Mission (NRHM) on April 2005. The NRHM was initially tasked with addressing the health needs of 18 states that had been identified as having weak public health indicators. Later the GoI made NRHM and National Urban Health Mission (NUHM) as sub-mission of the overarching National Health Mission (NHM) launched on May 2013. The GoI further launched the RMNCH+A (Reproductive, Maternal, Newborn, Child and Adolescent Health) strategy in 2013. The core principle of the RMNCH+A strategy was to implement a 'continuum of care' approach that would ensure sustained quality healthcare care services at various stages of reproductive lifecycle from pre-pregnancy to childbirth, followed by postnatal period and early childhood, also including the adolescent stage. Given the effort, there has been significant decrease in mortality and India's MMR stands at 130 per 100,000 live birth in 2016 from 212 in 2010, while NMR reduced from 44.4 in year 2000 to 24 per 1000 live births in 2016. Despite the progress made, to meet the current SDG goal of reaching 70 in MMR and 12 in NMR by 2030, further strengthening of the public health system and practices are required.

USAID's flagship project, Vriddhi, is a technical partner to the Government of India (GoI) and six state governments of Delhi, Haryana, Himachal Pradesh, Jharkhand, Punjab, and Uttarakhand. With the mandate of scaling up high-impact RMNCH+A interventions, Vriddhi, at the national level supports GoI to formulate evidence based policies and guidelines to implement RMNCH+A interventions. The project also facilitates concurrent evaluation of the programs across the High Priority Districts (HPDs) of the country through the National RMNCH+A Supportive Supervision system designed and implemented in collaboration with GoI and RMNCH+A partners. Across the six focus states, and 26 HPDs therein, Vriddhi provides support in planning, training, implementation, and monitoring for effective coverage and quality of all RMNCH+A interventions.

1.2 Project VRIDDHI: Care around Birth (CaB) Approach

Aligning with global priorities the "Care around Birth" approach, is central to the project Vriddhi - Scaling up RMNCH+A Interventions. Guided by WHO "Quality of Care (QoC)"framework for maternal and newborn health and the national RMNCH+A strategy, it is premised around the effective implementation of evidence-based Technical Interventions during intrapartum and immediate postpartum period – the most critical time for maternal and newborn survival. A holistic approach "Care around Birth", integrates technical interventions with Health Systems Strengthening (HSS) efforts, Quality Improvement (QI) techniques and Respectful Maternity Care (RMC) practices to optimize QoC at and around the time of birth.

Using the CaB approach, fourteen evidence based interventions were strengthened. These included: monitoring progress of labour, Active Management of Third Stage of Labour (AMTSL), Essential

Newborn Care (ENBC), newborn resuscitation, post-natal monitoring of mothers and newborn, initial management of post-partum Haemorrhage (PPH), initial management of Pre-Eclampsia / Eclampsia, Kangaroo Mother Care (KMC), feeding of Low Birth Weight (LBW) babies, management of maternal and newborn sepsis, use of Antenatal Corticosteroids for preterm labour and Prevention of Parent to Child Transmission (PPCT). The interventions were implemented in a phasic manner, where 5 interventions, namely monitoring progress of labour, AMTSL, ENBC, newborn resuscitation and postnatal monitoring were implemented in Phase 1. The current study assesses the implementation of first phase of interventions rolled out under CaB approach.

The implementation of the CaB approach was further steered by the findings from an initial baseline assessment done in 2015 by the Vriddhi Project team of the facilities in the intervention states to gauge key parameters on labour room services and infrastructure, staff clinical knowledge competency, and maternal and newborn care practices as per GoI Maternal and Newborn Health Toolkit and Standard Clinical Care guidelines. This was followed by the implementation of CaB approach in 141 high case load facilities from January 2016. The facilities identified had an annual delivery load of more than 200,000 births (≥68-70% of all institutional deliveries in Public health facilities within the district) and were distributed across 26 High Priority Districts (HPDs) in six states (Delhi, Haryana, Himachal Pradesh, Jharkhand, Punjab and Uttarakhand) of India.

States/Districts covered in CaB Approach

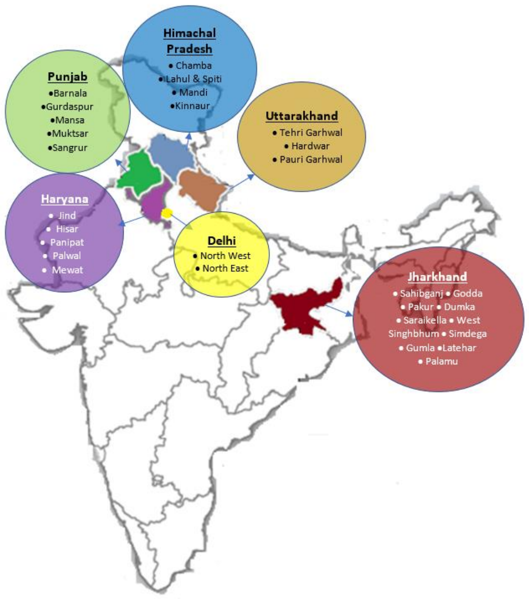

The CaB approach implementation involved enhancement of knowledge and skills of nursing staff through a set of experiential training strategy at both district and facility level which included both centralized and as well as onsite training. There was extensive use of simulation tools during the training sessions to better facilitate transferring and imbibing of learnings within the healthcare service providers. This was followed by systematically planned "low dose high frequency" on-site mentoring visits, which provided need based technical support to staff, and bridge the gaps in labour room management. Furthermore, for ensuring accountability and sustainability over time, facility level Quality Improvement (QI) teams were constituted, comprising of program managers and staff members. The intervention was further reinforced by the use of comprehensive MIS system. The MIS implemented by the Vriddhi project team to help capture clinical practices and quality service delivery indicators from the intervention facilities to measure change over time. This also helped in identifying challenges to improvement in service delivery, and helped guide the implementation process.

1.3 External Assessment

External assessment was conducted by Centre for Operations Research and Training (CORT) a social science research organization, from December 2017 to January 2018.

1.3.1 Objective of the Assessment

The overall objective of the study was to understand the modalities related to "Care around Birth" approach namely knowledge and current practices around maternal and newborn care, labour room environment and management of complications.

Specific objectives of the study were:

- To measure the improvement in labour room environment i.e. infrastructure, availability of drugs and consumables, recording and reporting etc. required for effective service delivery
- To measure the improvement in the competencies of service providers through skill based trainings for identifying critical interventions required at the time of birth for the mother and newborn
- To review, observe and assess the existing practices at the intervention facilities for essential care at the time of birth for the mother and newborn
- To undertake readiness assessment of the facilities for elements of Respectful Maternity Care (RMC)

1.3.2 Methodology

A mixed method approach consisting of quantitative and qualitative techniques were used to address the objective of the study. Quantitative component of the study included Labour room assessment checklist to measure improvement due to intervention, observations checklist to assess labour room services and practices, and knowledge assessment of healthcare providers. Qualitative component included structured interviews with beneficiaries at the facilities, in-depth interviews with Medical Officers in-Charge of the facilities, district officials and, state government officials. Quantitative data was collected on printed questionnaires whereas in-depth interviews were audio recorded after taking prior consent, which was later transcribed and manually coded thematically.

An IRB ethical clearance was attained for conducting the study (No. EC –CORT/1730). Oral or written consent were taken from each respondent before conducting an interview. The implementation partner IPE Global had pre-informed the concerned state authorities as well as the concerned district officials in the selected district about the External assessment being undertaken by CORT.

1.3.3 Study Universe

Care around Birth approach was implemented across 141 facilities in 26 high priority districts in six states. The distribution of facilities varied across the states of Delhi (8), Jharkhand (42), Haryana (32), Himachal Pradesh (16), Uttarakhand (18) and Punjab (25). Out of the 141 facilities 68 (48 percent) were L3 facilities, 70 (51 percent) were L2 facilities and 2 (1 percent) were L1 facilities. The two L1 facilities were the outliers among the facility pool and thus not considered during the External assessment.

1.3.4 Target group

The core objective of the assessment was to understand the programmatic impact of Care around Birth approach for improving intra and post-partum care in the intervention facilities. For the assessment the following target groups were reached out:

- Health facilities in which “Care around Birth” interventions were being implemented
- Health service providers (Doctor/ Nurse) working in labour rooms of the intervention facilities
- Women admitted in the intervention facilities for delivery
- State and District officials

1.3.5 Study tools

As this was a repeat assessment, to ensure the comparability of data with baseline findings, the study tools used for labour room assessment and health providers’ competency were adapted from the baseline tools. However, a separate set of structured questionnaire was prepared for woman admitted in the health facilities along with interview guidelines for medical officers in facilities and district and state level officials. Broadly, following set of tools were used for the study:

- ***Facility questionnaire (Labour room checklist):*** The facility assessment questionnaire was prepared to capture information on the infrastructure and layout of delivery room, human

resource, service provisions available in labour room, infection & waste management and data dissemination. Facility assessment questionnaire also had modules which captured information on display of IEC protocols, availability of equipment, medicines, drugs, vaccines and other consumables.

- ***Health provider questionnaire:*** A health provider questionnaire consisting of three modules was prepared to be administered to staff nurses and doctors posted in labour rooms. The first module consisted of questions related to the basic profile of the provider and the training they received on CaB approach. Knowledge of health providers regarding maternal health and essential newborn care practices in the labor room were assessed in the other two modules. The skills of the providers were also assessed by auditing sample of filled up partograph and checking their skills/knowledge on essential newborn care (ENBC) and newborn resuscitation using mannequins.

- ***Guidelines for interviewing Medical Officer, State and District officials:*** Guidelines were prepared for conducting in-depth interviews to gauge the impact CaB approach has made in maternal and child care services in the facility/district/state. Their views regarding the most significant changes observed, actions necessary to ensure its continuity, and suggestions to further improve the interventions was recorded.

- ***Observation checklist for evaluating management of labour and childbirth by healthcare providers:*** Observations of deliveries were carried out using structured checklist. It covered different stages of labour, care of mother and newborn till two hours post-delivery, and essential newborn care. Service delivery was observed to ascertain the quality of clinical practices healthcare provider trained under the CaB approach followed.

- ***Beneficiary questionnaire:*** A structured questionnaire was developed for women admitted in the postnatal ward of the facility after delivery to ascertain information regarding their perception on quality and availability of services, experience of care received during delivery and their satisfaction with services.

1.3.6 Sampling Design

Sampling of Facilities: Using WHO 'Service Availability and Readiness Assessment' (SARA) technique[1], a total of 49 facilities were sampled for the study, which was further increased to 51. Next using the probability proportional to size (PPS) methodology, from the distribution of L2 and L3 facilities in the study universe (139 facilities, excluding 2 L1 facilities), it was proposed to cover 26 L2 facilities and 25 L3 facilities. In the 2nd step, the number of facilities to be sampled were decided from the six states based on the proportion of these facilities in the states, and lastly the requisite number of facilities were selected randomly based on the level (L2 or L3) from the list of facilities within the state.

Selection of health providers: For the survey of health providers, it was proposed to cover the labour room staff nurses. The number of nurses posted in labour room depended on the case load of delivery in the facilities. According to the 'Guidelines for Standardization of labor rooms at delivery points' (Ministry of Health and family welfare's, NHM, GOI, March 2016) there should be a minimum of 3 ANMs in a labour room where case load was less than 50 deliveries and 5 ANMs for a case load of 50-100 deliveries per facility. Hence, the study planned to cover 3 health providers in L2 facility and 5 health providers in L3 facility making a total of 203 staff nurses.

For survey of Medical Officer, it was proposed to interview two Medical Officer at L3 facility and one

[1] http://apps.who.int/iris/bitstream/handle/10665/112798/WHO_HIS_HSI_RME_2013_2_eng.pdf;jsessionid=01BE04DA5A78AA3D17F9C67BFCD9DFDC?sequence=1

in L2 facility, thus reaching out to around 76 Medical Officers.

Sampling of beneficiaries: The required sample for beneficiaries was calculated using the standard formula for estimating the sample with a design effect of 1, level of significance (α) 0.05, and $z_{1-\alpha/2}$= 1.96. Based on the calculations the sample size was estimated to be 385, which was further rounded up to 400 (account for incomplete or non-response). In the next step, the 400 to be surveyed beneficiaries were distributed based on the proportion of case load (average one year case load) among the sampled 51 facilities.

In addition to the above, in each of the selected district and state, senior stakeholders had to be interviewed.

1.3.7 Sample Achievement

Coverage of Facilities and Beneficiaries

All 51 sample facilities were visited and data were collected as per the study protocol. Observation of delivery per facility however varied as it was dependent on the availability of delivery case in the facility during the team's presence there. To cover the shortfall of sample due to the absence of any cases or low delivery load, similar CaB intervention facilities (based on level of the facility and delivery load) were selected. Thus, two additional facilities each in Jharkhand and Haryana were visited to achieve the desired sample coverage. In these additional facilities only the beneficiaries were observed and interviewed. Table 1.1 provides the state-wise coverage of facilities and beneficiaries.

Table 1.1: Coverage of facilities and beneficiaries across the six states

State	Number of facilities covered			Number of beneficiaries covered		
	L2	L3	Total	L2	L3	Total
Delhi	1	2	3	0	72	72
Haryana	9	3	12	30	57	87
Himachal Pradesh	4	2	6	6	22	28
Jharkhand	9	6	15	56	84	140
Punjab	0	8	8	0	53	53
Uttarakhand	3	4	7	7	21	28
**All**	**26**	**25**	**51**	**99**	**309**	**408**

As per the study design, the beneficiary that is the woman visiting the facility for delivery service had to be observed for two hours post-delivery. Woman who were in early stages of labour were approached and inducted into the study. From the sample facilities a total of 408 beneficiaries were reached. Among these, 399 beneficiaries were observed and 392 were interviewed.

1.3.8 Interviews with stakeholders

Health providers: As per the previous section 203 nurses were planned to be interviewed, but due to lack of their time and availability of human resource, interaction was limited to 195 staff nurses. They were assessed for their basic profiles, trainings received, and knowledge and skills on maternal and essential newborn care services. In-depth interviews were conducted with 62 Medical Officers. The detailed break up is given in Table 1.2.

Table 1.2: Number of service providers interviewed in selected facilities

Type of facility	No. of facilities	Medical Officer	Nurse
L2	26	23	77
L3	25	39	118
**Total**	**51**	**62**	**195**

State/District officials: Two state level officials from each state were contacted for the interview. However, only one senior state officer could be interviewed in four states (Jharkhand, Uttarakhand, Haryana and Himachal Pradesh) due to the unavailability of the second official on account of their busy schedule and/or being on leave. Similarly, thirty three district level official in the selected

districts were interviewed for the study, while nine of them were not available.

1.3.9 Study Implementation

Training: The assessment team comprised of a combination of 8 GNM/ Nurse and 8 social scientists. A seven day training was organized for field team members in Jharkhand. This included both classroom training and actual field practice by visiting L2 (CHC) and L3 (district hospital) facilities in Ranchi district, Jharkhand. Training was imparted jointly by Vriddhi project team members and CORT team members.

Process of Data Collection: Data collection was initiated from the sample facilities in Jharkhand state. This was followed by data collection in Himachal Pradesh, Uttarakhand, Punjab, Haryana and New Delhi. In each study facility, a uniform study protocol was adopted for data collection with the team spending three days at each site. It included delivery observation, labour room assessment conducted by the field team nurse and post-delivery interview of beneficiary by the social scientist. The health provider's interview was either conducted by the field team nurse or the social scientist. Interview of medical officer was conducted by supervisors and /or field coordinator of CORT. All the district and state level officials' interviews were conducted by field coordinators of CORT and senior project in-charge from CORT.

1.3.10 Challenges during Assessment

Some major challenges encountered during data collection were:

- Conducting stakeholder interviews at the state and district level was difficult due to non-availability of officials or because of their busy schedule.
- Availability of service providers at facility for interview to a great extent depended on the case load, as they had to attend deliveries continuously.
- At L2 facilities due to low delivery case load, it was difficult to achieve the required sample of deliveries for observation as per the study design. To cover the shortage of sample, similar CaB L2 facilities had to be selected preferably from the same district. In many cases, the teams also stayed back in the facility for more than three days to cover the sample size. This increased the time required to complete data collection.
- In few cases, some of the observed beneficiaries could not be followed up for the interview when the team returned on the following day, as they were either discharged or referred to another health facility.

1.3.11 Data Management and Analysis

All open ended data were systematically coded and entered in a specially developed package in CSPro, which took care of range and consistency. Quantitative data thus entered in CSPro and then exported into SPSS for detailed and statistical analysis. The qualitative data which were mostly audio recorded, were subsequently transcribed and then coded thematically.

Weighting factor for data: Since the data was collected from a smaller sample, post-sampling weights were added to the data to obtain an estimate representing the universe of data, i.e. 139 facilities. The weights calculations were also done to enlarge the sample unit to estimate data at the facility of different levels (L2 and L3). The weights used for analysis of data are described below:

- **Weights according to level of facility**: The data collected for the study was from 51 sampled facilities (25 L3 and 26 L2) out of the study universe of 139 facilities (68 L3 and 71 L2). Thus a multiplier was developed on the basis of the total number of facilities of a level present in the state versus the sample taken to correctly amplify the results of the sampled facilities for the entire study universe.

State	Weights for Facilities	
	L2	L3
Delhi	2.0	4.0
Haryana	2.7	2.7
Himachal	2.5	2.5
Jharkhand	2.7	2.9
Punjab	3.0	0.0
Uttarakhand	2.8	2.3

- **Weights for service provider:** 195 service providers (staff nurses) were assessed/interviewed from the sampled 51 facilities, this was only a proportion of the total number of providers available in the study universe of 139 facilities. Thus to correctly represent the sampled data findings, appropriate weights were calculated on the basis of total number of service providers posted in the intervention facilities of different level for each state.

State	Weights for Service Providers	
	L2	L3
Delhi	8.5	14.5
Haryana	6.4	5.3
Himachal	7.6	4.8
Jharkhand	4.6	5.9
Punjab	5.9	0.0
Uttarakhand	8.1	5.3

- **Weights for beneficiary interviewed/ deliveries observed:** During the assessment a total of 408 delivery observations/beneficiary interviews were done in the 51 sampled facilities. To correctly represent the findings, weights were calculated on the basis of total number of reported normal deliveries conducted in the previous year (December 2016 to November 2017) in the respective implementation facility at different level.

State	Weights for Observations	
	L2	L3
Delhi	223.6	0.0
Haryana	447.7	741.4
Himachal	360.2	232.3
Jharkhand	421.6	446.8
Punjab	423.8	0.0
Uttarakhand	527.5	547.7

For each of the above components weights were calculated and applied, thus all analysis shown are calculated from the weighted figures.

Chapter 2
Facility Level Assessment

There are two major aspects that govern the quality of service available or delivered when a beneficiary visits a healthcare facility for services. The first aspect is the readiness or preparedness of the facility in terms of infrastructure, equipment, amenities, space, etc. to provide specific clinical care. The second crucial aspect is the cadre and competency of the healthcare providers available in the facility to deliver specific clinical care. Thus during the assessment the following aspects were assessed:

1. **Facility readiness:** to understand how well prepared facilities are to provide standard clinical care and how the CaB approach has made an impact in this aspect.
2. **Healthcare providers in labour room:** to understand the cadre of healthcare service providers present in the facilities, their level of competency, and if exposure to the CaB approach helped them improve their skills and eventually quality of services.
3. **Quality improvement teams:** The role QI teams played in improving facility readiness and competency, and improving the overall quality of service delivery.
4. **Stakeholders and healthcare provider perspective:** to highlight views of stakeholders at State, District and facility level over changes observed due to CaB approach implementation.

Findings related to the aspects described above have been discussed in this chapter.

2.1 Facility Readiness

A well-equipped healthcare facility is a key essential to ensure quality service delivery. Realizing its importance, the Ministry of Health and Family Welfare (MoHFW), Government of India (GoI) prepared Indian Public Health Standards (IPHS) for healthcare facilities at all levels, and Maternal & Newborn Health Toolkit (MNH Tool Kit). The CaB approach utilizes these existing guidelines and worked to impact the overall readiness of the facilities. For assessment the following aspects of facility readiness were investigated:

1. Infrastructure
2. Availability of Equipment
3. Protocols and IEC materials
4. Documentation
5. Infection prevention practices
6. Support/Ancillary services

2.1.1 Infrastructure

Labour room being one of the crucial space in the facility should have all necessary amenities available to ensure quality services to beneficiaries during delivery. All assessed facilities had a newborn care corner within the labour room. Further, 24x7 running water facility was available in labour room of all facilities, while 96 percent of facilities had electric supply with functional power back up (Annexure table A2.1). Presence of attached functional toilet, drinking water facility and air condition in labour room was relatively better in L3 facilities (functional toilet 82 percent, drinking water 80 percent, and air condition 72 percent) when compared to L2 facilities (functional toilet 78 percent, drinking water 49 percent, and air condition 30 percent). 55 percent of the facilities had windows with tinted glass in labour room, whereas 42 percent of facilities had windows with both tinted glass and see through glass with curtain. Functional refrigerator was available in 91 percent facilities that included all L3 facilities.

Comparing the amenities available during External assessment with baseline findings, data (Table 2.1) does indicate a positive improvement in the status. Change in percentage points varies from four

percent points for availability of drinking water facility in labour room to 31 percent points for functional refrigerator.

Table 2.1: Amenities in labour room during baseline and External assessment (in percentage)		
Particulars	Baseline	External Assessment
**Total number of health facilities assessed**	**141**	**139**
**% Facilities have**		
Newborn care Corner within Labour Room	96.5	100.0
Earmarked room for Sterilization/autoclaving	39.7	68.8
**Amenities available in Labour Room (LR)**		
Attached functional toilet facility in LR	74.5	79.9
LR have 24*7 running water facility	90.8	100.0
LR have drinking water facility	60.3	63.9
LR have 24*7 Electricity supply with functional Power Backup that includes invertor or generator	80.1	96.3
A functional Room Heater	75.9	84.1
LR air-conditioned	36.2	50.4
A functional Refrigerator	60.3	90.9

On an average two to three labour tables were available in labour rooms. Certain essential equipment like radiant warmer, baby weighing scale, fetoscope, adult stethoscope and blood pressure apparatus were available in all facilities, others like mackintosh on labour table, autoclaved delivery sets, functional wall clock and oxygen cylinder and ambu bag were available in over 92 percent of the facilities. Availability of adult oxygen hood (44 percent), modular light for conducting deliveries (57 percent), and functional pulse oximeter in labour room (64 percent) were comparatively low (Annexure Table A2.2).

Table 2.2: Medical equipment in labour room during baseline and External assessment (in Percentage)		
Particulars	Baseline	External Assessment
**Total number of health facilities**	**141**	**139**
**Type of equipment/furnishing**		
Mackintosh with each Labour Table (LT)	70.2	93.9
Functional Kellys pad on each of LT	41.1	78.9
Autoclaved delivery sets readily available	72.3	96.0
Modular Light for conducting deliveries	48.2	57.1
Functional wall clock with seconds hand / digital clock in LR	90.1	97.9
Wall mounted thermometer for measuring room temperature	61.0	85.9
Functional Haemoglobinometer with reagents and lancet	34.8	70.5
Functional suction apparatus in LR	35.5	83.8
Electric suction apparatus	63.8	78.0
Movable Delivery trolley in LR	66.7	84.0
Functional Oxygen cylinder in LR or central oxygen supply	75.2	92.4
Functional Pulse Oximeter in LR	16.3	63.7
BP apparatus	91.5	100.0
Adult Stethoscope	90.8	100.0
Fetoscope	77.3	100.0
Ambu bag	81.6	97.8
Oxygen hood (Adult)	41.1	44.2
Pediatric stethoscope	22.7	67.8
Baby weighing scale	95.0	100.0
Radiant warmer	89.4	100.0
Radiant warmer have a dedicated stabilizer	9.9	62.9

Equating the current findings with baseline data (Table 2.2) indicates an improvement in all the medical equipment/furnishing assessed in the Labour Room. However, the degree of change varies across the equipment's.

2.1.2 Availability of Equipment

Drugs and supplies are essential for both maternal and newborn care within the labour room before, during, and after delivery. All essential materials should be available in the labour room in the seven designated trays, namely, delivery tray, episiotomy tray, baby tray, medicine tray, emergency drug tray, Manual Vacuum Aspiration/Electric Vacuum Aspiration (MVA/ EVA) tray and PPIUCD tray (only at L-3 level). Presence of designated tray with designated materials (drugs and instrument) ensures smooth handling of the delivery process by the healthcare provider thereby ensuring quality services. During the assessment the minimum number of trays with all the essential items available for use were assessed.

While the delivery tray should have 12 elements, the medicine tray and PPIUCD tray should have seven elements each and eleven items constitute the newborn tray (Annexure Table A2.3, Annexure Table A2.4). The assessment analysed in terms of whether all components of the tray and the designated tray were available in labour room, as well as accessibility of all items in the tray. Data (Table 2.3) indicates that minimum one tray of each type was available in the facilities and there was improvement observed since the last baseline assessment.

Table 2.3: Status of designated trays in labour room during baseline and External assessment (in percentage)

Particulars	Baseline	External Assessment
**Total number of health facilities**	**141**	**139**
**Type of trays available**	**% Facilities**	**% Facilities**
Delivery tray	66.0	98.0
Episiotomy tray	39.7	91.1
Baby Tray or newborn tray	55.3	98.0
Medicine tray	51.1	97.1
Emergency drug tray	61.7	100.0
PPIUCD tray	56.0	94.4

Analysing all the elements of the trays regarding its availability in labour room and its presences in the designated tray, data (Table 2.4) indicates that although 86 to 91 percent of the facilities had all required items for delivery and PPIUCD tray respectively, but these items were available in the designated tray in only 27 percent (delivery tray) and 4 percent (PPIUCD tray), hence indicating that providers need to consciously ensure that all items are available within the trays at all time. Similar was the status with elements of medicine tray (Annexure Table A2.3).

Table 2.4: Status of delivery, medicine and PPIUCD trays during baseline and External assessment (in percentage)

Particulars	Baseline	External Assessment
**Total number of health facilities**	**141**	**139**
**Delivery tray**		
All items available	41.8	86.0
Tray available	66.0	98.0
All items in tray	5.7	26.8
**Medicine tray**		
All items available	11.3	45.6

Tray available	51.1	97.1
All items in tray	2.1	15.7
**PPIUCD tray**		
All items available	29.1	90.5
Tray available	56.0	94.4
All items in tray	3.5	3.9

2.1.3 Availability of protocols and IEC display in labour room and postnatal ward

For all healthcare facilities it is vital that essential protocols (for example, AMTSL, partograph, essential newborn care, hand washing, protocols etc.) on practices related to delivery, should be displayed in and around the labour room and clearly visible at appropriate places. This ensures that the staff nurses/ healthcare providers have quick access to essential protocols and guidelines at all times for reference.

Protocols which were observed in the labour room (Annexure Table A2.5) for maternal care included, simplified partograph (88 percent of the facilities), management of PPH (99 percent of the facilities), eclampsia (99 percent of the facilities), Active Management of Third Stage of Labour (AMTSL) (95 percent of the facilities), vaginal bleeding before 20 weeks (90 percent of the facilities), and vaginal bleeding after 20 weeks (94 percent of the facilities). Findings reveal that the protocols related to newborn care were also on display in labour room. For instance 99 percent of the facilities had newborn resuscitation protocol. However, two protocols Kangaroo Mother Care and breastfeeding protocol were present in 73 percent and 79 percent of the facilities respectively.

Level wise, the proportion of protocols present in L2 facility was better than in L3 facility. Presence of these protocols in external assessment indicates a significant change for the better from baseline findings (Table 2.5). During baseline the availability of protocols displayed in the facility ranged from 33-67 percent (with the exception of LR sterilization protocol 10 percent), while during External assessment, the same protocols availability ranged between 73 to 99 percent, with the exception of LR sterilization which was 59 percent.

Table 2.5: Display of protocols in labour room during baseline and External assessment (in percentage)

Particulars	Baseline	External Assessment
**Total number of health facilities**	**141**	**139**
Simplified Partograph	45.4	87.9
Active Management of Third Stage of Labour (AMTSL)	64.5	94.8
APH before 20 weeks	42.6	89.7
APH after 20 weeks	41.8	93.8
Management of PPH	67.4	98.6
Management of atonic PPH	37.6	94.4
Eclampsia	56.0	98.6
Breastfeeding	39.7	78.6
Kangaroo Mother Care	33.3	72.7
Newborn resuscitation	63.8	98.6
Hand washing	63.8	94.6
Preparation of 1 litre bleaching solution	40.4	94.8
Infection prevention	46.1	88.1
Processing of used items	33.3	90.1
LR Sterilization	9.9	58.8

IEC at Postnatal Ward: In addition to the labour room, the postnatal ward and nursing station were observed especially in context of display of IEC materials. Annexure Table A2.6 indicates that

breastfeeding protocol was found in 75 percent of the facilities, but other IEC materials were less displayed, like postnatal care within first two hours of birth (66 percent), KMC protocol (66 percent) etc. In comparison to the baseline findings, data for two protocols namely breastfeeding and KMC was available, which have almost doubled during the External assessment as compared to baseline.

2.1.4 Documentation

Availability of required registers and keeping it updated ensures appropriate documentation and follow up service (Annexure Table A2.7a). Among all the registers, referral (out) register and PPIUCD/ FP register was available in 97 and 96 percent facilities respectively, followed by discharge register (85 percent) and handing over register (86 percent). The availability of all registers (Table 2.6) reviewed was less during baseline as compared to their availability now during External assessment. Labour room sterilization register was available in 63 percent of the facilities where as it was only 19 percent during baseline. PNC registers were in 16 percent of the facilities during baseline, whereas External assessment findings indicates that it was 77 percent. During assessment, updated document were available in 65 to 82 percent of the facilities, while in baseline this was higher and ranged from 77 to 92 percent. So although more number of facilities started maintaining registers, there is still a requirement of efforts to ensure that these documents are kept updated.

Table 2.6: Registers available and updated in facilities during baseline and External assessment (in percentage)

Particulars	Baseline		External Assessment	
	Available	Updated	Available	Updated
Referral Register (Out)	85.8	91.7	96.5	75.8
Maternal death record Register	31.9	88.9	74.6	65.2
LR sterilization Register	19.1	77.8	62.6	78.4
Handing over-taking over Register	37.6	77.4	86.2	77.7
Discharge Register	42.6	91.7	85.3	76.9
PPIUCD / FP register	72.3	96.1	96.4	81.6
PNC Register	16.3	82.6	76.5	74.3

Stakeholders also highlighted the change in record keeping by the providers following the CaB approach implementation. According to them improvement in keeping records and documentation was one of the key intervention that had been strengthened on account of CaB approach. This enhanced the knowledge of the staff nurses in providing quality services following the CaB training. As a healthcare provider suggested

> *"Proper documentation and record should be maintained, timely equipment should be provided, regular training should be conducted by providing new guidelines, and regular meeting should be conducted for ensuring its continuity"*.

As a Medical Officer mentioned,

> *"After CaB approach in our facility our ANM, GNM, duty nurses do record keeping and documentation as per the scientific guidelines provided and explained during CaB training."*

Beside the availability of registers, the assessment had also reviewed case sheets for the last five deliveries that had occurred in the facility from the previous day. Analysis indicate (Table 2.7) that among the 697 deliveries documented, partograph was used in 93 percent of deliveries, and in almost all the deliveries oxytocin injection was administered within a minute of delivery. Newborn children had received the appropriate birth dose vaccine as well as Vitamin K1 prior to discharge. Data further reveals that 95 percent of mothers were counselled for postpartum family planning use and 32 percent had accepted a family planning method. Among the 19 percent births that had Asphyxia at

birth, 90 percent of them were resuscitated successfully. Availability of such data in the register would facilitate the program managers to monitor and support the services provided in the facility.

Table 2.7: Selected indicators from documented data of last five deliveries in register (in Percentage)	
**Particulars**	**External Assessment**
**Total number of deliveries documented from register**	**697**
Partograph used for the delivery	93.2
Injection Oxytocin administered within 1 minute of delivery	98.8
Delivery outcome as "Live birth"	99.3
Newborn given birth dose of vaccines (OPV+BCG+ Hep B)	97.7
Newborn given vitamin K1 before discharge	100.0
Newborn had Asphyxia at the time of birth	19.2
Newborn successfully resuscitated if had Asphyxia at the time of birth	89.7
Mother was counseled for postpartum family planning (PPFP) methods	94.9
Mother accepted any postpartum family planning (PPFP) method	32.4

2.1.5 Infection prevention Practices

Ensuring infection control within the health facility is imperative. In the current assessment, infection prevention practise/standards were assessed on:

- Availability of essential consumables and amenities, and
- Availability of personal protection items

Amenities and Consumable for Infection Prevention: Almost all (98 percent) facilities had hand washing facility at the point of use (Annexure Table A 2.8). However, 88 percent (facilities) had elbow operated taps. All facilities had staff trained in preparing cleaning solution (0.5% chlorine) as per the standard protocol. Access to labour room with external footwear was restricted in 87 percent of the facilities. Amenities required for standard bio medical waste management was largely available in facilities (needle cutters, disinfection of sharp disposal and decontamination of instruments after use 100 percent each; colour coded bins 98 percent; puncture proof box 92 percent; colour coded plastic bags 86 percent and sterilization/autoclaving 69 percent) (Table 2.5).

Availability of Items for Personal Protection: Availability of items for personal protection from infection is imperative. Findings from the study indicate that certain personal protection items like sterile gloves (100 percent), masks (98 percent), and gown/apron (98 percent) were largely available in the facilities during the External assessment as compared to baseline (Table 2.8). Similarly, caps and heavy duty gloves were available in 86 and 85 percent facilities respectively. Shoe cover/gum boots was available in 70 percent of the facilities. Whereas, the availability of personal protective kit for delivering HIV positive patients was 57 percent overall, with 76 percent in L-3 level facilities and 39 percent in L-2 level facilities.

Table 2.8: Material and management of Infection prevention during baseline and External assessment (in percentage)		
**Particulars**	**Baseline**	**External Assessment**
**Total number of health facilities**	**141**	**139**
**Hand hygiene & antisepsis**		
Hand washing facility at Point of Use (Sink with running water)	94.3	98.0
Elbow operated taps	46.1	87.8
**Material for personal protection**		
Availability of Masks	75.9	98.0
Sterile gloves are available at labour room	95.7	100.0
Gown/ Apron	84.4	97.9

Shoe cover/gum boots	33.3	70.4
Caps	59.6	86.1
Heavy duty gloves and gum boots for housekeeping staff	23.4	84.8
Personal protective kit for delivering HIV positive patients	35.5	57.2
**Environment control of patient care areas**		
Staff is trained for preparing cleaning solution (0.5% chlorine solution) as per the standard procedure	73.8	100.0
External footwear is restricted	42.6	87.1
**BMW management**		
Colour coded bins at point of waste generation	88.7	98.0
Plastic colour coded plastic bags	68.1	86.2
Segregation of different category of waste as per guidelines	79.4	98.0
There is no mixing of infectious and general waste	79.4	98.0
Functional needle cutters	94.3	100.0
Puncture proof box	57.4	92.3
Disinfection of sharp before disposal	56.0	100.0
Transportation of bio medical waste is done in close container/trolley	68.1	90.4
Instruments dipped in 0.5% chlorine solution immediately after use (decontamination)	76.6	100.0
**Method of waste disposal**		
Onsite burying	44.0	41.0
Onsite incineration	7.1	19.6
Agency collection	53.9	64.6
Others	-	7.7
**Separate pit(s) available for Placenta**	57.4	94.5

2.1.6 Support/Ancillary services at Facility

Data (Annexure Table A2.9) regarding other support services which the health facilities require indicates that 63 percent facility had help desk /enquiry, the status varied in L3 (80 percent) and L2 (46 percent) levels. Even though security service should be there at all hours in the health facility, but this was available in 53 percent of the facilities. Overall 94 percent facilities had dietary services for indoor patients (L3 -100 percent; L2 -89 percent). Display of citizen charter at prominent place in the facility was present in 82 percent. Availability of an enquiry/help desk improved to 63 percent during External assessment as compared to baseline (27 percent). In all these indicators a change from its situation during baseline to External assessment is observed (Table 2.9), however, the extent varies across the indicators.

Table 2.9: Support services at facility during baseline and External assessment (in percentage)

Particulars	Baseline	External assessment
**Total number of health facilities**	**141**	**139**
Facilities have an enquiry/Help desk	27.0	62.9
Facilities have Security services at all hours	28.4	52.5
Facilities has dietary facility/service for indoor patients	85.8	94.3
Citizen charter displayed at prominent place in facilities	70.2	81.5
**Facilities having ambulance/referral transport facility**		94.3
108 available		60.4
102 available		32.2
Ambulance of health facility available		49.5
Others (Mamta Vahan)		20.1

Health facility should have functional transport service available. Availability of ambulance at the facility is crucial for timely transporting/referring of the patient. Data indicates that 94 percent of facility had ambulance service available. Nature of ambulance service varied from 108 ambulance (60 percent), 102 ambulance (32 percent) and health facility own ambulance (50 percent) service. There are sometimes overlapping services available too where the facility avails 108/102 along with personal ambulance. In Jharkhand, a separate service called Mamta Vahan was available in 67 percent facilities for transporting mothers.

Display of IEC material either in the form of poster or banner or semi-permanent fixtures in the admission area of the facility and/ or in the OPD area are means of sharing information with the beneficiaries. For maternal health service information on JSY and JSSK entitlements, referral transport, complaint box and signage at the entrance are means of guiding and informing the beneficiaries during their facility visit. Findings (Annexure Table A2. 10) do indicate that 85 percent of the facilities had display of JSSK entitlements, while JSY was displayed in 78 percent facilities, all displays were either present in the admission/registration area or at the entrance of the facility. In around 38 to 36 percent these displays were in local language. Almost similar was the situation for referral transport (84 percent display and 38 percent in local language). Over 80 percent of the facility had complaint box and signage displayed, but in local language it was only 12 and 16 percent respectively. Availability of the display in local language possibly would be more useful to the beneficiaries.

2.2 Healthcare providers in Labour Room

As discussed earlier in parallel to improve the readiness of the facility the CaB approach also focused on increasing the competency of staff nurses/ ANM working in labour room. The approach had concentrated on enhancement of knowledge as well as the skills of the healthcare providers. To enable this change specialized training package had been innovatively designed under the CaB approach which had sessions and contents designed to contain a combination of demonstration and actual practice on mannequins along with theoretical inputs. The key competencies covered during capacity building included:

- Using partograph for monitoring delivery,
- Active management of third stage of labour (AMTSL),
- Essential newborn care after birth including resuscitation,
- Postnatal monitoring of mother and newborn till discharge, and
- Infection prevention

Combination of techniques were used to strengthen the capacity of service providers. This included two days centralized training at the district level, followed by facility based hand holding support extended by IPE Global District Training Officer who made regular visits to the facility. Subsequently with the formation of Quality Improvement teams within the facility, in house support (at the facility level) was also rendered as per the need. The current External assessment was carried out to understand the following:

1. **Profile of the Labour room staff**
2. **Capacity building strategy used under the CaB approach:** this includes opinion of the different stakeholders on the strategy used
3. **Competency of the labour room staff**: the results of the CaB approach in making a change in the competency level of healthcare providers

2.2.1 Profile of Labour Room staff

During the survey, a total of 195 healthcare providers with 118 in L-3 level and 77 in L-2 level who were posted in labour room were interviewed. Among these providers, 85 percent were staff nurse and 15 percent were ANM (Annexure Table A2.11). Variation in healthcare provider's designation was observed across the level of facility. Qualification wise, 74 percent had done their 'diploma in nursing'

and 15 percent had done 'B.Sc. in nursing'. These healthcare providers were working as staff nurse/ANM on an average for a little more than 9 years. Apart from the duties in labour room, they also had posting in other departments of the facility (Annexure Table A2.12).

In the previous two years, nurses had received trainings on Post-partum IUCD (46 percent), Navjat Shishu Suraksha Karyakram (NSSK) (40 percent), Skilled Birth Attendant (SBA) (35 percent) and Daksh skill labs (23 percent) (Annexure Table A2.13).

2.2.2 Capacity building under CaB approach

External assessment data indicates that 46 percent of the nurses had participated in the district level training (Annexure Table A2.14). Subsequently, most of the healthcare providers from the labour room were reached out during mentoring and hand held support of the district coordinator, and/or, QI teams in the facility. In the process, almost all the healthcare providers of labour room in the study facilities of the six states had been reached under CaB approach (Figure 2.1).

Figure 2.1: Percentage of healthcare providers participated in CaB training

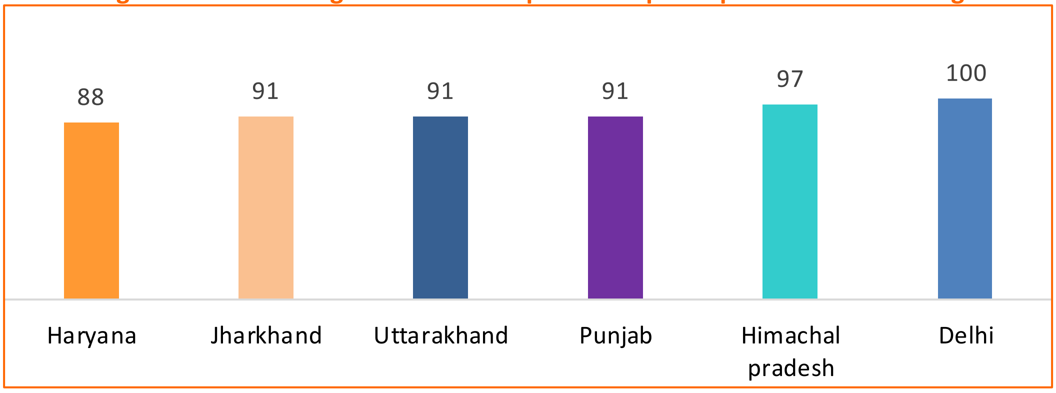

Topics covered during the training as mentioned by nurses included Essential newborn Care and Resuscitation (96 percent), Active Management of Third Stage of Labour (95 percent), Essential Childbirth Care and Labour monitoring (91 percent) and postnatal monitoring and counselling at discharge (87 percent). The topic of newborn vaccination was reported by 58 percent of nurses.

Irrespective of the process of orientation under the CaB approach, overall, 92 percent of the healthcare providers had received CaB training. Apparently the pattern of receiving the training was almost similar across L3 (91 percent) and L2 (93 percent) level facilities.

Methods used during training were explored from the healthcare providers' perspective. Most commonly reported method included practice on mannequins (97 percent), demonstration (96 percent), and role plays (83 percent) (Annexure Table A2.15). To a lesser extent use of other techniques like working in small groups (68 percent), case studies (50 percent) and visualization in participatory planning writing (47 percent) was recalled and reported. Exploring as to which method actually helped the provider to enhance the providers' knowledge and skills, 56 percent of the providers mentioned the technique of demonstration, followed by role plays (34 percent) and practice on mannequins (27 percent) were preferential over other methods.

State level stakeholders also had emphatically mentioned that capacity building technique used in CaB approach was different from the routine training programme, as a lot of individual practice, support and guidance was provided. The modality used in training was novel and had contributed in raising the knowledge and skills of the healthcare providers.

Analysing the opinions of the healthcare providers regarding the advantage of participating in the care around birth training, wide spectrum of responses cutting across the CaB themes emerged (Annexure Table A2.16). The areas mentioned wherein the training helped in improving competency were provision of better overall care for mother and newborn, and ability to monitor mothers in better manner and thereby save their lives. With the training skill acquired, the healthcare providers could assess the condition of the mother better, improve their decisions regarding whether the delivery would be normal or caesarean, conduct deliveries well and referral had become easier. Adhering to the steps of delivery, procedure of delivery had become easier, and labour room was prepared beforehand which saved time and improved quality. It was also mentioned that oxytocin was given within one minute and they were able to check and control/manage PPH well. Similarly for care of the newborn, the healthcare providers were of the belief that they could take good care of the newborn baby in safe way using correct methods. Resuscitation had improved and was initiated in the labour room itself. They could also decide on referrals in timely manner. Initiation of skin to skin contact by putting child on mother's abdomen after birth was done in labour room itself. Mothers were provided information about benefits of immediate breast feeding and they were motivated to start breastfeeding on labour table itself with proper positioning.

In the words of a healthcare provider,

> *"In CaB training we were taught how to fill up partograph, this guided us to take decision when to refer the patient, and it also became easy for us to manage patient ourselves. Earlier we did not do resuscitation and we used to call out to doctor, but now after CaB training by doing resuscitation we can save the life of child. We learnt the procedure of drying the baby in proper sequence and also about KMC. On account of participation in the training, it is easy to provide service as we follow the steps beginning from delivery being conducted till the discharge of baby."*

Further it was also added by one of the healthcare provider

> *"We came to know many new things like providing oxytocin to the mother within one minute after delivery. It was also helpful to be a member of QI team. We learnt about preparedness of the labour room before delivery, filling up of partograph, keeping baby on abdomen after delivery, providing skin to skin contact, delayed cord clamping"*
>
> *Due to CaB approach, we got the job aids & protocol posters with the support of Senior Medical Officers (SMOs) in the facilities. In our facility the posters were displayed in the labour room so that during delivery in case there were any difficulties the staff would read the poster and solve their problems."*

According to the healthcare providers, apart from training, the other activities undertaken as a part of the approach in the facility were found to be useful including maintenance of registers /records (79 percent) and formation of Quality improvement teams (75 percent) (Annexure Table A2. 17). Since keeping the different types of registers/ records was done properly, it had become easier to check on details and provide follow up services. They also learnt on how to utilize the documented data. In the process work was done properly and easily. Supportive monitoring extended by QI teams was seen to be very helpful, as problems were discussed and solved during monthly meetings. Regular monitoring did help in improving the services provided.
One of the healthcare provider said

> *"I am receiving support from the day I have joined from January 2017. It has been helpful to take a decision regarding patient through filling up of partograph of mother and foetal heart rate of child during their progress of labour. Post-partum haemorrhage has been managed by*

providing oxytocin within one minute of the delivery, due to which referral has decreased. Earlier baby who did not cry was shifted to newborn care unit but after training we provide resuscitation and try to stabilize the child in labour room only. Earlier soon after delivery mother was shifted to postnatal ward but now we keep mother in labour room only for at least one hour and monitor her bleeding in every 15 minutes. We also counsel mother for initiation of breastfeeding within one hour of birth with proper positioning and attachment".

Even, medical officers spoke positively about the training imparted to healthcare providers. They did comment that the process of capacity building was very interesting and staff nurses were able to handle the various stages of labour properly and postnatal care had also improved. As one medical officer said,

"During training, demonstration was done very nicely about the process of delivery, how birth takes place, how to use towels for drying baby, how to resuscitate the baby. Through cards also it was explained what were the things need to be done in providing services. There is a positive impact of training on staff nurses. Following the training and mentoring in the facility, changes took place in the labour room, all staff nurses became alert on how they need to keep their labour room prepared."

Overall the responses from the district officials also cut across different components of CaB approach, ranging from preparedness of labour room before delivery, management of various stages of labour, maintaining cleanliness of labour room and maintenance of records.

2.2.3 Competency of Labour Room staff

Competency of labour room staff nurses is the next crucial aspect that governs the quality of service provided to beneficiary. The competency of the staff was assessed for partograph, AMTSL, essential newborn care, post-natal monitoring and infection prevention, the six thematic areas covered in CaB approach. Competency was assessed by using the same set of questions used during the baseline. Competency in each of the thematic area is discussed separately in the following paragraphs.

Partograph: To assess the competency of healthcare providers in filling up the partograph, a case study of a woman who was pregnant for the first time and admitted in the hospital with labour pains was shared with them. The case study encapsulates details following woman's admission, foetal heart rate at various hours, dilatation of cervix, status of amniotic fluid, blood pressure measurement, time, temperature recorded and pulse were presented, that had to be filled in partograph. A comprehensive analysis of all the components of the questionnaire together as well as independently was done. Overall 34 percent of providers achieved a score over 80 percent, while 59 percent scored between 51-80 percent (Annexure Table A2.18). Positive shift has been observed in competency level from baseline to External assessment in all components of partograph (Table 2.10)

Table 2.10: Competancy score of healthcare providers in AMTSL during baseline and External assessment across level of facilities (in percentage)

	Baseline				External assessment			
Particulars	<50 %	51-80%	80%+	Total	<50 %	51-80%	80%+	Total
L3 level	66	23	12	224	5	63	31	738
L2 level	64	22	15	200	9	52	39	438
Total	65	22	13	424	7	59	34	1176

One of the key challenge in implementation of partograph for monitoring delivery was the inadequacy in the number of staff of the facility, the same was highlighted by a medical officer who mentioned the constraint which he found among his staff nurse in preparing partograph. In his words,

"There is only one challenge which we face and that is inadequacy of staff. When there is so much of rush, details are missed out like filling of partograph and frequent post-natal check-ups of both mother and baby."

Active Management of Third stage of labour: Active management of the third stage of labour (AMTSL) is a critical component for reducing maternal mortality rate as it helps in managing postpartum haemorrhage among the women during pregnancy. AMTSL is composed of two components or steps:

1) Administration of an uterotonic, preferably oxytocin, immediately after birth of the baby (recommended within 1 min post birth); and,
2) Controlled cord traction (CCT) to deliver the placenta

Ten questions addressing various components of AMTSL were addressed to healthcare providers in the form of MCQs. Broadly it included the stages of labour, critical components, administration of uterotonic, choice and storage of this drug, practice of AMTSL, causes of postpartum haemorrhage etc.

Overall 62 percent of healthcare providers scored above 80 percent in AMTSL competency which is an increase from the baseline assessment (33 percent) (Table 2.11). However 8 percent of the providers during External assessment still scored less than 50 percent. Competency of L3 level providers who had scored above 80 percent was relatively better (68 percent) than L2 level (51 percent).

Table 2.11: Competancy score of healthcare providers in AMTSL during baseline and External assessment across level of facilities (in percentage)

	Baseline				External assessment			
Particulars	<50 %	51-80%	80%+	Total	<50 %	51-80%	80%+	Total
L3 level	17	49	34	224	6	26	68	738
L2 level	13	56	32	200	11	37	51	438
Total	15	52	33	424	8	31	62	1176

Essential newborn Care: To test the competency a series of questions related to the care of newborn were assessed from the healthcare provider (Annexure table 2.19 to 2.24). Scores in each of the broad components have been separately analysed (Table 2.12). It was encouraging to note that over 80 percent of healthcare providers scored over 80 percent on the components of preparation in labour room, resuscitation, and newborn vaccination. In case of Routine baby care after birth 70 percent of providers scored over 80 percent. These percentages are a marked difference when compared to baseline data especially for preparation in the labour room, routine baby care after birth and resuscitation, wherein over 50 percent of the healthcare providers had scored less than 50 percent. Capacity building of providers on these components in CaB approach has contributed to this change.

Table 2.12: Competancy score of healthcare providers in New Born Care during baseline and External assessment across level of facilities (in percentage)

	Baseline				External assessment			
Particulars	<50 %	50-80%	80%+	Total	<50 %	50-80%	80%+	Total
**Preparations in the Labour Room (22)**								
L3 level	52	41	8	224	2	17	81	738
L2 level	52	37	11	200	2	10	87	438
Total	52	39	9	424	2	15	83	1176
**Routine baby care after birth (57)**								

L3 level	73	20	7	224	2	26	72	738
L2 level	59	34	8	200	4	30	66	438
Total	66	26	7	424	2	28	70	1176
**Resuscitation (36)**								
L3 level	71	24	6	224	0	17	83	738
L2 level	65	31	4	200	3	18	80	438
Total	68	27	5	424	1	17	82	1176
**newborn vaccination**								
L3 level	24	17	59	224	5	1	94	738
L2 level	22	5	74	200	8	1	91	438
Total	23	11	66	424	6	1	93	1176

Healthcare provider competency score was assessed on counselling that needs to be provided at the discharge of the baby and when the women should return (Annexure Table A2.25). During the baseline assessment 88 percent of the assessed healthcare providers had scored less than 50%, by the time of External assessment the number reduced to 16 percent. Similarly changes were seen for counselling to be given to mother on care of newborn at the time of discharge.

Postnatal Monitoring: The postnatal period of mother and newborn is the phase where systematic monitoring is the most important step. This is the period where the mother and newborn can be assessed to know if there are any underlying complications/danger signs that can lead to potential mortality or morbidity. This period is also best for the healthcare provider to connect with the beneficiary (mother) and her family to provide necessary counselling on care of newborn and mother.

A comprehensive analysis taking all the components of postnatal monitoring together (Table 2.13) indicated 32 percent of the providers above 80% which was an overall improvement since baseline. A positive shift in the competency of healthcare providers was observed since baseline in the External assessment.

Table 2. 13: Competancy score of healthcare providers in Postnatal Monitoring during baseline and External assessement across levels (in percentage)

Particulars	Baseline				External assessment			
	<50 %	51-80%	80%+	Total	<50 %	51-80%	80%+	Total
L3 level	44	49	7	224	35	36	29	738
L2 level	38	54	8	200	22	41	37	438
Total	41	51	8	424	30	38	32	1176

Infection prevention: Regarding knowledge on prevention and management of infection, healthcare providers were asked various elements related to decontamination of instruments, handling and disposal of bio waste, hygiene practice, preparation of chlorine solution etc. (Annexure Table A2.26). Overall competency assessment of all the ten elements taken together indicates that 43 percent of providers got over 80 percent score in External assessment which was only 6 percent at baseline assessment (Table 2.14).

Table 2.14: Competancy score of helath providers in infection prevention during baseline and External assessment across levels (in percentage)

Particulars	Baseline				External assessment			
	<50 %	51-80%	80%+	Total	<50 %	51-80%	80%+	Total
L3 level	39	55	6	224	18	38	44	738
L2 level	42	53	6	200	17	41	42	438
Total	40	54	6	424	18	39	43	1176

2.3 Quality Improvement (QI) teams

Quality improvement teams had been established in the facilities as part of CaB approach to facilitate in supporting and improving the services in labour room. Ninety four percent of providers stated that Quality improvement teams were established in their facility as part of "Care around Birth" approach. Sixty three percent of them were members of these teams.

Contribution of QI teams

- Healthcare providers reported/monitored work done
- Guidance/discussions on clinical activities related to childbirth and post-delivery care; Re-trainings provided if needed
- Identification of gaps in the facility and deciding on action to be taken; Discussion on strengthening of Logistics /infrastructure
- Increase interpersonal team work between members of the facility and improve cooperation.

One of the healthcare provider mentioned that:

> *"Guidance from QI team has helped us in managing PPH among women and providing resuscitation to babies."*

Further it was also added by another healthcare provider:

> *"Benefits of QI meeting ensured timely vaccination of babies, proper recording and documentation of registers. Orientation on normal deliveries was done through demonstrations. Equipment's like radiant warmer, PPH kit, room thermometer which were not available earlier in the facility were made available."*

Talking about the Quality improvement team a medical officer said:

> *"Yes, QI team has been formed. I was involved with the team. We do conduct meetings and decide which area needs to be focused. Identify the targets which are not achieved or are lagging behind. We focus on these discussed topics and try to improve it. For example, there was no insertion of PPIUCD in our facility as none of the sisters were trained for it. But now after training all staff nurses knew about it and they do insert PPIUCD."*

Even the district stakeholder added:

> *"Yes, there is a Quality improvement team who analyzes the quality of services provided during intra and postnatal period. All the activities (filling of partograph, newborn resucitation, AMTSL, use of oxytocin) are conducted in the facilities and have become a regular routine practice among the staff nurses."*

All state stakeholders beyond the facilities had talked about formation of QI teams at the facility level for improving quality of services in the labour room. They also added that on account of QI team, logistic issues were being resolved, clinical services standards has improved, and performance data was being tracked and improved in a very systematic way.

2.4 Stakeholders and healthcare provider's perspective on changes due to CaB approach implementation

The positive changes in the facility preparedness and competency of the healthcare providers was acknowledged by all stakeholders. Medical officers, district and state officials all acknowledged the contribution of CaB approach in improving the overall quality of service delivery at the facility level.

In the words of stakeholders:

- *"Various activities are undertaken as part of the "Care around Birth" approach, this includes preparedness of labour room for newborn, radiant warmer is kept on, towels are kept ready, tray for baby is kept ready, windows of labour room are closed. As soon as baby is delivered oxytocin*

is given to mother, procedure of drying baby and initiating breast feeding within first hour is undertaken."

- *"Realizing the importance of infection prevention, staff nurse do ensure that the steps for hand washing, sterilization of instruments etc. are followed."*
- *"Major visible change is seen among the labour room staff nurses, they have become more confident in providing services and they know the exact procedure of conducting deliveries."*

District stakeholders acknowledged that timely clinical interventions were being provided to mother and child which have contributed in decreasing the mortality and morbidity rates. Almost similar views resonated among the state stakeholders that the staff nurses would ensure better quality of services, especially those who had undertaken CaB approach training.

Healthcare providers did suggest ways to ensure the continuity of changes in long term (Annexure Table A2.27). Almost all the healthcare providers comprehended that the program should carry on. Sixty percent of providers who reported that QI teams was a major change, wanted to continue their work and monitor the staff, encourage people to adhere to the process, discuss and clear the doubts which arise. Forty percent providers also suggested that regular trainings should be arranged for juniors, new recruits and those who did not attend the training. Twenty percent provide rs reported that they would follow all the AMTSL steps, do all the required preparation beforehand and keep things ready for delivery and newborn care. Further, 11 percent providers said that all members should be actively involved.

On enquiring from the healthcare providers if there were any skill or methods that was difficult to perform despite training, 37 percent of the providers re sponded 'yes' (Annexure Table A2.28). This included maintaining privacy, curtains/screens between labour tables (33 percent), unable to do KMC in labour room (18 percent) and completing partograph as per requirement (15 percent). To a lesser degree, issues of initiating breastfeeding in labour room (6 percent), cleaning with bleaching solution (4 percent), convincing mothers about PPIUCD (4 percent), use of radiant warmer (3 percent), resuscitation in some cases (3 percent) etc. were also reported.

Elaborating on the reasons why the providers could not use their skills or provide the required service was on account of lack of staff /lady doctor (42 percent), size of labour room was small (20 percent) and therefore not possible to have everything inside the room. Providers also expressed lack of confidence and felt the need of more training (11 percent). Further another 11 percent providers also perceived that patients do not understand and they do require more counselling. Some other reasons mentioned included workload is more, no time (10 percent), damaged/non-functional equipment's (9 percent), ambulance not available 24 hours (8 percent), lack of bed/ labour tables (6 percent), lack of instrument (6 percent) and depends on patient condition (3 percent).

Challenges narrated by Medical Officers in providing services revolved more around the characteristic of the beneficiary and the socio- cultural norms which they bring along with them, as it was reported,

> *"Women seeking maternal health services in the facility were largely anaemic, illiterate, multigravida, lack awareness regarding family planning methods, and frequent occurrence of PPH, all of which poses challenge for service providers in ensuring quality services."*
>
> *"The major problem in initiating breast feeding within one hour are the social and cultural beliefs of the beneficiary as well as their family members, due to which they initiate pre lacteals as a first drink and also sometimes start giving tea instead of mother's milk."*

> *"Illiteracy and socio cultural beliefs are hindrance for women to stay in hospital for 48 hours following delivery."*

While 9 percent of providers did not have any suggestion for further improvement of CaB in their facility (Annexure Table A2.29), 42 percent suggested that the shortfall in staff has to be fulfilled. Further all staff need to undergo training and doctor should be available for 24 hours. CaB training as a regular activity every year was suggested by around 35 percent providers. It was suggested that refresher training should be there for those already trained and the new comers should receive the complete training. This has to be accompanied by more practical training. Need for strengthening of logistic was mentioned by 19 percent and 11 percent stressed the need for proper maintenance of equipment like spot light, electric suction machine, oxygen cylinder etc.
In the words of a healthcare provider,

> *"Time to time training and continuous monitoring should be there for improving quality of services. More number of trained staff nurses should be posted in a labour room for providing quality of services"*

Chapter 3
Quality of Services

Increase in utilization of healthcare facilities for maternal and newborn care, also sets in concern for the quality of care that is provided. The quality of care received by woman during pregnancy, delivery and postpartum period affects her overall health and also impacts the health of newborn. Poor quality of care can contribute to increase in both maternal & newborn morbidity and mortality. Improving quality of care at the facility also ensures increased beneficiary and their family satisfaction with the healthcare services provided, thereby improving the likelihood of beneficiaries returning for services in the future.

To gather evidence on the impact CaB approach implementation made on the quality of services at the intervention facility the assessment team collected information from two main sources:

1. **Service delivery observations at different stages of labour**: observation of healthcare services provided to the beneficiary at the different stages of labour during childbirth, and followed by immediate post-partum care.
2. **Client perspective (beneficiary feedback):** interview beneficiaries regarding the various components of quality pertaining to services availed and amenities available in the facility including **Respectful Maternity Care (RMC).**

3.1 Service Delivery Observations

As discussed above the observations were recorded as per the different stages of labour during childbirth. All the observations were carried out by nurses within the assessment team who filled out a checklist pertaining to practices related to different stages of pre/post-delivery.
A total of 399 beneficiaries were observed across the multiple stages of delivery, among these 14 were cases of newborn complication that required resuscitation.

3.1.1 First stage of labour

Observing the practices followed in preparing the labour room for delivery (Annexure Table A3.1), data indicates that for preparing the labour room for delivery a draught free environment was ensured in 100 percent of all the facilities. Other preparations made to take care of the newborn included, keeping the radiant warmer switched on (99 percent), placing two clean towels under the radiant warmer (95 percent), checking for functional bag and mask (91 percent), and maintaining a newborn tray with all items (90 percent) before delivery. In 99 percent of the facilities the delivery tray was kept ready with all essential items beforehand, similar preparation were made for infection prevention by the staff nurse, as they had washed their hands as per the protocol (96 percent) and had worn sterile gloves (100 percent) before all clinical procedures.

3.1.2 Second stage of labour

Upon observations (Annexure Table A3.2) the assessment team found that the progression of labour was monitored through partograph in 75 percent of the beneficiaries observed. Post-delivery, providers initiated skin to skin contact by keeping the delivered baby on mother's abdomen (97 percent), time of birth was called out (98 percent), and newborn was assessed for meconium and cry (98 percent) at birth and dried as per the guidelines (98 percent) from head to toe.

In the sample survey there were 14 births in total (9 in L3 and 5 in L2 level) that required resuscitation. Observing the steps followed for resuscitation, the standard clinical care sequence was largely followed; suction done immediately (93 percent); baby shifted to newborn care corner (93 percent); baby positioned with shoulder roll and suction done (86 percent); baby stimulated (100 percent); baby repositioned for suction (88 percent); bag and mask applied correctly (90 percent), resuscitation

initiated within first thirty seconds of birth (96 percent); resuscitation done as per guideline (77 percent) and monitoring of heart rate as per protocol (84 percent). Overall the assessment data pointed that the providers were following all essential steps as and when required.

3.1.3 Third stage of labour

Findings from the observations (Annexure Table A3.3) indicated that prefilled syringe with Uterotonic (Oxytocin) was kept ready as the cervix was completely dilated and was administered within a minute of delivery by all (100 percent) the healthcare providers. Further, in all deliveries the practice of cord cutting within one to three minutes followed by removal of placenta by CCT was done. Complete examination of placenta as per the protocol was seen in 75 percent of deliveries, and safe disposal of placenta was done in 99 percent of the cases. In 96 percent of the observation, instruments were placed in 0.5% chlorine solution for 10 minutes for decontamination after use.

Comparing the practice of administration of oxytocin from baseline findings to the External assessment, as seen in Figure 3.1 in almost all deliveries the practice of administration of oxytocin and within one minute was done during External assessment. This is a significant positive change from the baseline findings.

Figure 3.1: Practice of Administering Oxytocin during baseline and External assessment (in %)

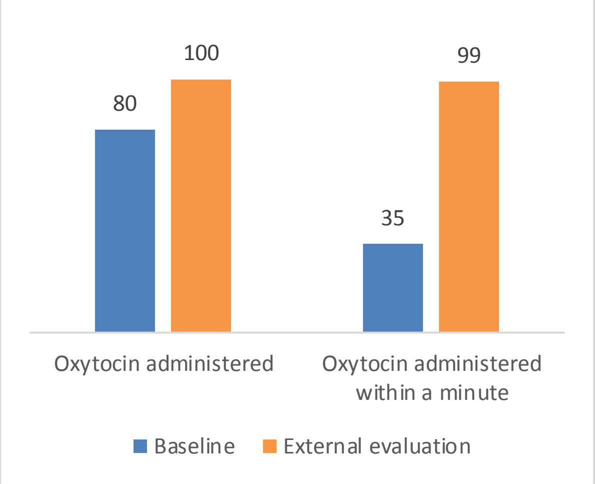

3.1.4 Fourth stage of labour

Post-natal monitoring of mother and newborn for overall condition and any signs of complication is the most critical step in this stage. Findings from observation of mother and newborn during post-natal period (Annexure Table A3.4) indicated that the mother was kept warm and comfortable (98 percent), was not separated from the newborn unless required (95 percent), and the baby was kept in skin to skin contact with the mother (86 percent). As for the newborn, breast feeding was initiated in 96 percent, and injection vitamin K1 was administered to 97 percent newborns. In 80 percent of cases, newborns were assessed for proper positioning and attachment during breastfeeding. It was noted that correct positioning during breastfeeding was done more in L2 facilities (91 percent) in comparison to the L3 (75 percent) facilities. Similarly a difference in L2 (86 percent) and L3 (67 percent) was noted when assessing post-natal vital monitoring of newborn after 30 minutes of delivery. According to the assessment one of the possible reason for the discrepancy in the service delivery between L2 and L3 is due to high case load in L3 facilities. Among the newborns that were observed, 99 percent of them were weighed at birth, followed by examination from head to toe in 91 percent, temperature was recorded for 88 percent, and respiratory rate was recorded for 87 percent newborns. The data also highlighted that 85 percent of the providers washed their hands before any examination.

3.2 Client Perspective

During the assessment beneficiaries who had utilised the services of the facility for delivery were observed and interviewed. A total of 392 beneficiaries were interviewed among which 230 were from L3 level and 94 from L2 level.

3.2.1 Profile of beneficiary

The detailed profile of the beneficiaries who came to the healthcare facility is listed in table 3.1 (Annexure Table A3.5). In summary, the average age of the mothers interviewed was 24. Also 56 percent of the beneficiaries were arriving at the facility from another village/town, hence emphasizing the importance of location of the facility, and the mode of transportation in increasing accessibility to healthcare facility/services.

Location of facility and mode of transport: It was reported by the beneficiaries that on an average they travelled around 18 km to reach the healthcare facility. Beneficiaries who had to avail services from an L-3 health facility had to travel on an average 20 km, whereas for L-2 facilities it was 12 km. The time and distance required to travel to a health facility is crucial as delays in reaching health facilities is one of the major reason for maternal morbidity/ mortality. Consequently, availability and utilization of ambulance service is ideal as it serves in the continuum of healthcare from home to facility. Sixty nine percent of the beneficiaries in L-2 facilities had used the ambulance services (102 or 108), whereas only 49 percent of beneficiaries in the L-3 facilities had done the same. Some of the reasons for not using the ambulance by the beneficiaries included, no need (18 percent), or the ambulance did not come (2 percent), or the ambulance didn't come in time (2 percent). The average out of pocket expense incurred towards transport was Rs. 324 (Annexure Table A3.6).

Table 3.1: Profile of beneficiaries (in percentage)

Particulars	Total
**Total number of beneficiaries (unweighted)**	**392**
Average age of beneficiary (in years)	24.4
**Place of Residence with respect to location of health facility**	
Resident of same village/town	44.0
From another village/town	56.0
**Location and services availed in health facility**	
Average distance of health facility from village/ town (in Kms)	18
Percent beneficiaries used ambulance services (102 or 108) to reach health facility	55
Average amount paid towards transport (in Rs.) (Out of pocket)	324
**Obstetric history of beneficiary**	
**Average number beneficiary had**	
Pregnancies	2
Current living children	2
**Sex of the newborn child**	
Male	52
Female	48

Beneficiaries were accompanied to the facility mostly by their husbands and/or mother-in-law, mother (Figure 3.2).

Figure 3.2: Person who accompanied beneficiary to facility (in %)

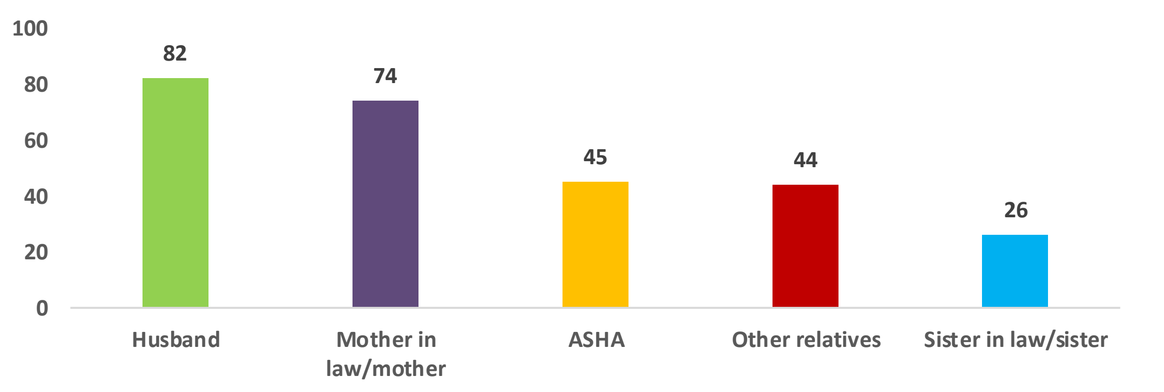

Ninety five percent of the beneficiaries came directly from home to the facility whereas 5 percent

were referred to the present facility.

3.2.2 Service received

To assess the level of satisfaction, post observation of a delivery the beneficiaries were followed up to enquire about their experience of care and satisfaction with services.

Services related to basic amenities: On enquiring about the cleanliness of the facility (Table 3.2) 99 percent of the beneficiaries acknowledged that the labour room was clean, while 96 percent found the post-natal ward to be clean. On enquiring about the cleanliness of the toilets, 84 percent of the beneficiaries considered the labour room toilets to be clean and 87 percent found the post-natal ward toilets to be clean. The beneficiaries (93 percent) found 24 hours running water in the toilets, and 81 percent found drinkable water source in the facility. 72 percent of the beneficiaries acknowledged the availability of clean bed sheets in ward. 72 percent of the beneficiaries availed free diet services (Annexure Table A3.8).

Table 3.2: Services received on arrival (in percentage)

Particulars	Total
BP was checked at the time of admission in labour room	85
Staff Nurse/ANM put her hand over abdomen at regular intervals during labour	99
Stethoscope put over abdomen at regular intervals during labour	88
Was comfortable during the examination	84
Privacy maintained during the examination or labour so that people other than facility staff could not see beneficiary	97

Services received on arrival in the facility: All beneficiaries were asked about their experience with the services they received on arrival at the healthcare facility. Most of the beneficiaries were checked for blood pressure (BP) and received regular examinations on arrival (Table 3.2). During examinations the beneficiaries were comfortable (84 percent) and almost all felt that their privacy was adequately maintained.

Table 3.3: Services received by the beneficiary (in percentage)

Particulars	Total
**% beneficiaries opined maintenance of cleanliness/hygiene in**	
Labour room	99
Ward	96
**Facility of clean toilets in**	
Labour room	84
Ward	87
**Toilets had 24 hour running water**	93.2
**Drinking water available throughout the stay in hospital**	81.4
**Provision of clean bed sheets in ward**	71.7
**Free diet services received from facilities**	71.7

Care of newborn after birth: Almost all beneficiaries (99.5 percent) accounted that their newborns were put on abdomen immediately after delivery (Annexure Table A3.9) to initiate skin to skin contact. Newborns were mostly kept with the mother (93 percent) and were examined (72 percent) as part of standard care immediately post birth.

Services received during post-natal period: Initiation of breastfeeding within first hour of delivery is crucial as it ensures that the infant receives the first milk (colostrum) which is rich in protective factors. Findings indicate that 86 percent of beneficiaries had initiated breastfeeding within the first hour of birth (Annexure Table A3.9), 81 percent of the beneficiaries were provided necessary support for initiating breastfeeding and 87 percent beneficiaries did admit that the nurse and/or doctor had checked them or newborn during the post-natal period. For mother, the check-up done during post-natal visit included measuring blood pressure (59 percent), checking vaginal bleeding (44 percent), recording temperature (31 percent), and monitoring pulse (25 percent). For newborn, check-up included observing proper breastfeeding (60 percent), general condition examination (52 percent), recording temperature (37 percent), checking normal passing of stools (23 percent), and monitoring umbilical stump for infection (8 percent).

Beneficiaries also informed that they had received information/counselling from the providers. The counselling included post-partum family planning (51 percent), maintenance of hygiene and hand washing (44 percent), and continuation of exclusive breastfeeding (41 percent) for first 6 months. Counselling information was also provided regarding general upkeep for mother like, eating regular meals (23 percent), drinking fluids regularly (18 percent), and for the newborn to not apply anything on the cord stump (17 percent) and monitor for danger signs. 49 percent of beneficiaries stated that they were able to ask questions about themselves and their newborn health when required from the healthcare provider.

Respectful Maternity Care: Respectful maternity care (RMC)[2] is a universal human right that is due to every childbearing woman. During the CaB approach trainings the healthcare providers were sensitized over the different rights in RMC and encouraged to follow them to improve the experience of care for the beneficiaries. Women's experiences with clinical care in labour room can empower and comfort them, and this in turn can improve the utilization of the healthcare facility services. Drawing the findings from observation of the client and provider interaction in labour room and from the interview of the beneficiaries, this section attempts to present and understand various components that befalls in the seven rights listed under RMC.

Evidence of RMC from observation: Data from the observation of beneficiaries in labour room reveals that privacy was maintained during delivery in 84 percent of the cases observed. Presence of partition between labour tables was one of the strategy used to ensure privacy (Annexure Table A3.10). In almost all (98 percent) observations, the behaviour of the service provider with the beneficiary was gentle, comforting and reassuring, and the tone of the service provider in providing support/ encouragement was soft and friendly in 98 percent of the cases. It was observed that to ensure better comfort for the beneficiary shifting from labour room to post-natal ward was done on stretcher in 47 percent of the cases. In 78 percent of the cases observed, a companion of woman's choice was present inside the labour room.

Evidence of RMC from beneficiary feedback: Almost all beneficiaries (99.8 percent) acknowledged that on their arrival at the health facility, a staff member had attended to them (Annexure Table A3.11). Overall almost all beneficiaries affirmed that respect /dignity was maintained during vaginal (99 percent) and abdominal (98.5 percent) examination. 97 percent of beneficiaries responded that the service provider were supportive emotionally and encouraged them throughout the process of delivery. Beneficiaries who had experienced any form of abuse was 5 percent in L3 level and around 3 percent at L2 level. 87 percent of the beneficiaries perceived that the service providers were respectful to them during clinical services, and 90 percent of beneficiaries reported that the behaviour was comforting.

Upon enquiring the beneficiaries if their complaints/concerns were satisfactorily addressed, 75 percent of them mentioned that they did not have any complaints (Annexure Table A3.12), and 21 percent had their complaints satisfactorily addressed. Beneficiaries' satisfaction on the way they were attended by the staff was also probed and around two third (67 percent) of the beneficiary said that they were 'satisfied', 31 percent responded 'very satisfied' with the services received. Almost similar response was obtained when enquired about the care and treatment received (33 percent 'very satisfied; 66 percent 'satisfied) in the health facility. Almost all beneficiaries (99.7 percent) reported

[2] ****There are seven basic rights in RMC: 1.** Right to be free from harm and ill treatment; **2.** Right to information, informed consent and refusal, and respect for her choices and preferences, including companionship during maternity care; **3.** Right to privacy and confidentiality; **4.** Right to be treated with dignity and respect; **5.** Right to equality, freedom from discrimination and equitable care; **6.** Right to healthcare and to the highest attainable level of health; **7.** Right to liberty, autonomy, self-determination and freedom from coercion

that they would like to return to the same facility to seek any health service in future and the same also shared that they would recommend their family members and friends to utilize similar services.

Stakeholder interviews: Interviews with stakeholders across the state, district and facility also concluded that considerable changes in quality of service had happened on account of CaB approach Discussing about the current interaction/ relationship between providers and the beneficiaries, Medical Officers were of the view that even though the staff nurses were always cordial to the beneficiaries the CaB approach has caused further improvement in that, as one Medical Officer stated,

> *"Yes, there is a change in the attitude of our staff nurses, before there was a hesitation in providing the services but now there is no hesitation, secondly, our staff nurses do selfless work. With no discrimination, they behave and provide services equally to all the beneficiaries."*

Further 50 percent of the medical officers interviewed stated that due to CaB approach, their staff nurses gave more respect, maintained the dignity, and ensured privacy of beneficiaries as part of RMC.

Overall, during the External assessment the findings clearly indicate an enhancement in the competency level of staff nurses related to clinical service delivery during and around the period of childbirth. The learnings from the CaB training not only impacted the quality of the services but also helped in further improving the RMC essential during service delivery.

Chapter 4
Conclusion and Recommendation

Adopting a comprehensive "Care around Birth" (CaB) approach, Vriddhi worked on improving practices for maternal and newborn care in public health facilities. The approach was built upon Evidence Based Technical Interventions (EBTI), Health System Strengthening (HSS) efforts, Quality Improvement (QI) methodologies and Respectful Maternity Care (RMC). The approach was implemented since January 2016 in 141 high case load facilities distributed across 26 High Priority Districts (HPDs) in six states (Delhi, Haryana, Himachal Pradesh, Jharkhand, Punjab and Uttarakhand) of India.

To assess the outcomes of the approach an external assessment was conducted in select intervention facilities across the project states. Overall 51 of the 141 intervention facilities were assessed on labour room environment, staff competencies and practices and beneficiary perspective. 195 staff nurses / ANMs were assessed for competency and 65 medical officers were interviewed to assess the provision of care at the facilities. Similarly, 33 district level and 8 state level officials were also interviewed. In addition, 399 deliveries were observed, and 392 beneficiary interactions conducted to assess the experience of care. Furthermore, appropriate weights were applied, and the data was extrapolated to all the 141 intervention facilities.

Findings from External assessment were compared with previous baseline assessment (Oct, 2015 – Dec, 2015) conducted by Vriddhi project team, to help identify changes if any at the intervention facilities.

4.1 Readiness of labour room

As per the assessment findings the infrastructure in labor rooms (LR) improved since the baseline. Positive changes were visible in basic amenities and facility support services. Improvement was also evident in the presence of all the medical equipment /furnishing assessed in the LR. Availability of dedicated 7 trays and their respective items in the LR improved since baseline. The assessment team also found positive efforts towards infection prevention in labor room. In comparison with the situation during baseline the current assessment found documentation and recording practices improved and systematic. However, despite the positive changes variability was observed in the findings across the facilities.

Recommendations

1. To sustain the changes made and ensure future gaps are identified and closed, there is a need to create a facility level monitoring, planning, and management plan. Quality Improvement (QI) teams set up in each facility can support such activities. The Vriddhi team needs to strengthen the QI teams on these activities by further training/support.

4.2 Competency level of Health Providers

The Vriddhi team designed a comprehensive training package for competency enhancement across the intervention facilities. Training sessions were conducted either at the district level or through onsite mentoring and were participatory in nature with the use of mannequins, role plays, demonstration etc. Upon enquiry, healthcare providers did assert that the training was participatory and improved their knowledge & skills. This statement was further endorsed by stakeholders at both district and state levels.

Overall competency scores improved across the interventions included in the "Care around Birth" package. Findings revealed better preparedness of labor rooms, improved ability to monitor progress

of labour, provision of AMTSL, ENCR, postnatal monitoring and infection prevention. Observation of deliveries further corroborated the translation of knowledge into practice.

Staff members however expressed constraints across certain areas like maintaining of privacy in the labor rooms, intra and interdepartmental coordination, low confidence levels and extended duty hours.

Recommendations

1. Challenges cited above point towards the need to address issues beyond clinical competency. It is important that service providers acquire skills on time management, team building and inter-personal communication. These skills are essential to service providers especially in high case load facilities with low human resource capacity. Though the "Care around Birth" approach has these aspects incorporated in the clinical training modules, it is recommended that Vriddhi team incorporate such soft skill aspects as separate training modules within the approach.
2. To ensure sustainability of the skills and knowledge it is necessary that facility staff undergo timely refresher trainings. It is therefore recommended that appropriate structure and mechanism are created within the government system to imbibe and strengthen efforts before the Vriddhi support is withdrawn.

4.3 Client perspective on quality of service

Interviews with beneficiaries revealed satisfaction with services provided in the intervention facilities. The beneficiaries also appreciated availability of different amenities. Beneficiaries were comfortable during examination and felt their privacy was maintained. Mothers informed that they and their newborns were monitored on a timely basis and provided satisfactory care. Beneficiaries could freely interact with service providers and upon enquiring had their complaints/ concerns satisfactorily addressed. However, it was recorded that a large number (56 percent) of beneficiaries travelled from adjoining towns or villages to the intervention facilities. It was also found that despite availability of ambulance services (102/108 services) many beneficiaries couldn't avail their services either due to lack of knowledge/ in-availability of ambulance/ delay in arrival of the ambulance. This led to both delay in clinical care and out of pocket expenses.
On elements of Respectful Maternity Care (RMC), in almost all the observed cases (99.7 percent), the behavior of service provider was gentle, comforting and reassuring. Beneficiaries felt the service provider were supportive, encouraging, and comforting.

Recommendations

1. It is recommended that the scope of the "Care around Birth" approach needs to be expanded to incorporate referral management, and consultation with state and district officers/stakeholders needs to be initiated to address the same.
2. Ensuring patient satisfaction is a challenge as it involves more behavioral change rather than clinical practices in service providers. To ensure sustainability of current positive changes, it is recommended to establish mechanism for regular counseling of healthcare providers on elements of RMC. As recommended during the previous section, the same trainer/ mentor pool can also be utilized for this purpose. Also a client feedback loop system needs to be established for monitoring patient satisfaction and grievances redress.

4.4 Crosscutting recommendations

- To ensure availability of trained human resource a system must be put in place at the district/state level that ensures identification of training needs and monitoring of performance. Further non-rotation policy needs to be implemented within the facility to ensure retention of trained service providers.

- Quality improvement (QI) and quality assurance (QA) has been an integral element of the CaB approach. To ensure the sustainability of the efforts made in QI and QA at the facility level it is recommended that spectrum of the CaB approach should be broadened in line with the National Quality Assurance Standards
- One of the key reasons for visible improvement through the approach was the data driven change adopted by the Vriddhi team. A strong monitoring and feedback mechanism was established which enabled stakeholders at all levels to assess and improve outcomes. Thus, the Vriddhi team needs to work with the state NHM to strengthen labor room data management systems.
- Looking at the achievements, the Vriddhi team should undertake advocacy with the state government to scale up the CaB approach beyond the intervention facilities.

Annexure

Table A2.1 Infrastructure and layout of delivery unit/labour room				
	Baseline	External evaluation		
Particulars		L3	L2	Total
**Total number of health facilities**	**141**	**68**	**71**	**139**
**Layout of Labour Room Unit**				
**% Facilities have**				
newborn care Corner within Labour Room	96.5	100.0	100.0	100.0
Earmarked room for Sterilization/autoclaving	39.7			
**Amenities available in Labour Room (LR)**				
Attached functional toilet facility in LR	74.5	82.1	77.8	79.9
LR have 24*7 running water facility	90.8	100.0	100.0	100.0
LR have drinking water facility	60.3	79.9	48.7	63.9
LR have 24 *7 Electricity supply with functional Power Backup that includes invertor or generator	80.1	96.3	96.2	96.3
A functional Room Heater	75.9			84.1
LR air-conditioned	36.2	72.3	29.5	50.4
A functional Refrigerator	60.3	100.0	82.2	90.9
**Windows of labour room**				
With tinted glass	-	54.0	56.2	55.1
With tinted glass + with see through glass with curtain	-	43.8	39.8	41.7
With see through glass with curtain + with broken windows	-	0.0	4.1	2.1
Iron window with curtain	-	3.9	0.0	1.9
No window	-	6.9	0.0	3.4
Wooden window	-	0.0	4.1	2.1

Table A2.2 Medical equipment /furnishing in Labour Room				
	Baseline	External evaluation		
Particulars		L3	L2	Total
**Total number of health facilities**	**141**	**68**	**71**	**139**
Mean no. of labour tables available	2.3	2.7	2.5	2.6
**% of Equipment/Furnishing available**				
Mackintosh with each Labour Table (LT)	70.2	100.0	88.1	93.9
Functional Kellys pad on each of LT	41.1	82.3	75.7	78.9
Autoclaved delivery sets readily available	72.3	96.0	95.9	96.0
Modular Light for conducting deliveries	48.2	76.3	38.8	57.1
Functional wall clock with seconds hand / digital clock in LR	90.1	100.0	95.9	97.9
Wall mounted thermometer for measuring room temperature	61.0	93.1	79.0	85.9
Functional Haemoglobinometer with reagents and lancet	34.8	65.2	75.5	70.5
Functional suction apparatus in LR	35.5	92.0	75.9	83.8
**Type of suction apparatus does the LR have**				
Electric	63.8	92.0	64.6	78.0
Movable Delivery trolley in LR	66.7	92.2	76.2	84.0
Functional Oxygen cylinder in LR or central oxygen supply	75.2	87.9	96.7	92.4
Functional Pulse Oximeter in LR	16.3	64.5	63.0	63.7
BP apparatus	91.5	100.0	100.0	100.0

Table A2.2 Medical equipment /furnishing in Labour Room				
	Baseline	External evaluation		
Particulars		L3	L2	Total
Adult Stethoscope	90.8	100.0	100.0	100.0
Fetoscope	77.3	100.0	100.0	100.0
Ambu bag	81.6	95.6	100.0	97.8
Oxygen hood (Adult)	41.1	55.9	33.1	44.2
Paediatric stethoscope	22.7	65.1	70.3	67.8
Baby weighing scale	95.0	100.0	100.0	100.0
Radiant warmer	89.4	100.0	100.0	100.0
Radiant warmer have a dedicated stabilizer	9.9	65.3	60.5	62.9

Table A2.3 Items are available in designated Trays and/or outside the tray (in percentages)							
	Baseline	External evaluation					
Particulars		L3		L2		Total	
**Total number of health facilities**	**141**	**68**		**71**		**139**	
		**In Designated tray**	**Outside tray**	**in Designated tray**	**Outside tray**	**in Designated tray**	**Outside tray**
**Items available in designated Trays and/or outside the tray**							
**In delivery tray**							
Scissors	50.4	93.0	7.0	100.0	0.0	96.6	3.4
Artery forceps	53.2	96.0	4.0	96.5	3.5	96.2	3.8
Sponge holding forceps	51.1	96.0	4.0	100.0	0.0	98.0	2.0
Speculum	37.6	82.7	17.3	87.1	12.9	85.0	15.0
Urinary catheter	27.7	43.9	56.1	64.7	35.3	54.5	45.5
BP blade / surgical blade for cutting cord	14.9	47.3	52.7	71.7	24.2	59.8	38.1
Cord clamps and ligatures	25.5	59.0	41.0	75.2	24.8	67.3	32.7
Bowl for antiseptic solution	18.4	63.9	32.2	65.7	30.5	64.8	31.4
kidney tray	24.1	84.2	11.8	75.7	20.5	79.9	16.2
Gauze pieces; cotton swabs	27.7	67.0	33.0	72.5	27.5	69.8	30.2
Sanitary pads	22.0	47.3	44.7	56.8	31.3	52.2	37.8
Gloves	22.0	51.2	48.8	60.6	35.6	56.0	42.0
**In Medicine tray**							
Inj. Oxytocin (to be kept in the refrigerator)	29.1	59.1	40.9	70.0	30.0	64.7	35.3
Inj Gentamycin	27.7	51.5	40.4	88.9	11.1	70.6	25.4
Inj Vit K1	19.9	83.1	16.9	85.4	14.6	84.3	15.7
Tab Misoprostol	28.4	91.2	8.8	85.7	14.3	88.4	11.6
Tab Nifedipine	27.7	80.6	12.5	70.3	7.0	75.3	9.7
Tab Methyldopa	19.1	40.1	19.8	46.0	10.5	43.1	15.1
Tab Magnesium sulphate	43.3	65.2	19.8	69.0	14.6	67.1	17.1
**PPIUCD tray**							
PPIUCD insertion forceps	45.4	87.5	8.5	92.7	4.1	90.2	6.2
Sims speculum	38.3	91.5	8.5	89.4	0.0	90.5	4.1
Sponge holding forceps	41.1	91.5	4.0	92.9	3.8	92.3	3.9

Table A2.3 Items are available in designated Trays and/or outside the tray (in percentages)							
	Baseline	External evaluation					
Particulars		L3		L2		Total	
Cu IUCD 380A in sterile pack	29.8	51.8	27.8	57.1	31.5	54.5	29.7
Cu IUCD 375 in sterile pack	14.9	50.1	24.6	50.0	28.3	50.1	26.5
Cotton swabs	19.1	62.6	37.4	63.2	33.5	62.9	35.4
Betadine solution	11.3	37.4	62.6	53.3	43.4	45.5	52.8

Table A2.4 Availability of items in Labour room newborn tray				
	Baseline	External evaluation		
Particulars		L3	L2	Total
**Total number of health facilities**	**141**	**68**	**71**	**139**
**newborn tray**				
newborn thermometer	33.3	95.6	95.9	95.8
Self-inflating bag for New Born resuscitation	93.6	100.0	100.0	100.0
Mask-neonatal size (0)	80.1	100.0	95.9	97.9
Mask-neonatal size (1)	88.7	100.0	100.0	100.0
Oxygen hood (neonatal)	38.3	67.8	40.4	53.7
Dee Lees mucus extractor	90.1	100.0	100.0	100.0
Shoulder roll	63.1	100.0	100.0	100.0
Two pre warmed towels/sheets for wrapping the baby	73.8	96.0	100.0	98.0
Sterilized thread /cord clamp	96.5	100.0	100.0	100.0
Needle (26 gauge) and syringe (1ml)	55.3	100.0	91.9	95.9
Inj. Vitamin K1	58.9	95.6	100.0	97.8
% facilities had the items of the newborn tray available	-	58.9	40.4	49.4

Table A2.5 Display of protocols in LR				
	Baseline	External evaluation		
Particulars		L3	L2	Total
**Total number of health facilities**	**141**	**68**	**71**	**139**
**Display of Protocols in LR**				
Simplified Partograph	45.4	85.2	90.6	87.9
Vaginal bleeding before 20 weeks	42.6	88.7	90.6	89.7
Vaginal bleeding after 20 weeks	41.8	93.1	94.4	93.8
Management of PPH	67.4	97.1	100.0	98.6
Eclampsia	56.0	97.1	100.0	98.6
Active Management of Third Stage of Labour (AMTSL)	64.5	93.4	96.2	94.8
newborn resuscitation	63.8	97.1	100.0	98.6
Kangaroo Mother Care	33.3	77.5	68.1	72.7
Breastfeeding	39.7	77.3	79.8	78.6
Hand washing	63.8	89.0	100.0	94.6
Preparation of 1 litre bleaching solution	40.4	93.0	96.5	94.8
Infection prevention	46.1	89.0	87.3	88.1
Processing of used items	33.3	93.0	87.3	90.1
LR Sterilization	9.9	73.2	45.1	58.8
Management of atonic PPH	37.6	97.1	91.9	94.4

Table A2.6 Display of IEC materials in Postnatal ward (in percentages)				
	Baseline	External evaluation		
Particulars		L3	L2	Total
Breastfeeding	39.7	76.0	74.9	75.4
Post-Partum Family Planning	-	60.0	41.1	50.3
Post-natal care within 1st two hours of birth	-	68.4	64.1	66.2
Post-natal care after 1st two hours of birth	-	60.0	73.8	67.1
Discharge counselling	-	0	0	0
Set of six RMC posters for service providers	-	0	0	0
One RMC poster for beneficiary	-	57.3	41.1	49.0
KMC Protocol	33.3	60.5	71.4	66.1

Table A2.7a Registers available and updated in facilities (in percentages)								
	Baseline		External evaluation					
Particulars			L3		L2		Total	
	Available	Updated	Available	Updated	Available	Updated	Available	Updated
Referral Register (Out)	85.8	91.7	97.1	73.3	96.9	73.0	96.5	73.1
Maternal death record Register	31.9	88.9	80.9	64.6	68.7	33.5	74.6	48.7
LR sterilization Register	19.1	77.8	71.9	55.9	53.7	42.7	62.6	49.1
Handing over-taking over Register	37.6	77.4	83.7	67.8	88.6	66.2	86.2	67.0
Discharge Register	42.6	91.7	87.6	71.6	83.0	59.8	85.3	65.5
PPIUCD / FP register	72.3	96.1	100.0	88.0	92.9	69.7	96.4	78.6
PNC Register	16.3	82.6	75.6	59.7	77.3	54.1	76.5	56.8

Table A2.7b Analysis of documented deliveries from register (in Percentages)			
Particulars	L3	L2	Total
**Total number of deliveries documented from register**	**340**	**357**	**697**
Partograph used for the delivery	89.9	96.4	93.2
Injection Oxytocin administered within 1 minute of delivery	100.0	97.6	98.8
Delivery outcome as "Live birth"	99.2	99.3	99.3
Newborn given birth dose of vaccines (OPV+BCG+Hep-B)	98.4	96.9	97.7
Newborn given vitamin K1 before discharge	99.2	99.3	99.3
Newborn had Asphyxia at the time of birth	21.7	16.5	19.1
Newborn successfully resuscitated if had Asphyxia at the time of birth	17.7	16.5	17.1
Mother was counselled for postpartum family planning (PPFP) methods	92.6	97.1	94.9
Mother accepted any postpartum family planning (PPFP) method	29.5	35.1	32.4

Table A2.8 Infection Prevention in the Labour Room				
	Baseline	External evaluation		
Particulars		L3	L2	Total
**Total number of health facilities**	**141**	**68**	**71**	**139**
**Availability in the Labour Room**				
**Hand hygiene & antisepsis**				
Hand washing facility at Point of Use (Sink with running water)	94.3	96.0	100.0	98.0
Elbow operated taps	46.1	84.9	90.6	87.8

**Material for personal protection**				
Availability of Masks	75.9	96.0	100.0	98.0
Sterile gloves are available at labour room	95.7	100.0	100.0	100.0
Gown/ Apron	84.4	100.0	95.9	97.9
Shoe cover/gum boots	33.3	71.9	68.9	70.4
Caps	59.6	96.0	76.7	86.1
Heavy duty gloves and gum boots for housekeeping staff	23.4	92.2	77.8	84.8
Personal protective kit for delivering HIV positive patients	35.5	76.2	39.1	57.2
**Environment control of patient care areas**				
Staff is trained for preparing cleaning solution (0.5% chlorine solution) as per the standard procedure	73.8	100.0	100.0	100.0
External footwear is restricted	42.6	96.1	78.4	87.1
**BMW management**				
Colour coded bins at point of waste generation	88.7	96.0	100.0	98.0
Plastic colour coded plastic bags	68.1	88.0	84.6	86.2
Segregation of different category of waste as per guidelines	79.4	96.0	100.0	98.0
There is no mixing of infectious and general waste	79.4	96.0	100.0	98.0
Functional needle cutters	94.3	100.0	100.0	100.0
Puncture proof box	57.4	96.0	88.9	92.3
Disinfection of sharp before disposal	56.0	100.0	100.0	100.0
Transportation of bio medical waste is done in close container/trolley	68.1	88.1	92.7	90.4
Instruments dipped in 0.5% chlorine solution immediately after use (decontamination)	76.6	100.0	100.0	100.0
**Method of waste disposal**				
Onsite burying	44.0	35.8	46.0	41.0
Onsite incineration	7.1	7.8	30.8	19.6
Agency collection	53.9	72.3	57.3	64.6
Others	-	11.8	3.8	7.7
**Separate pit(s) available for Placenta**	57.4	96.1	92.9	94.5

Table A2.9 Support/Ancillary Services at Facility

	Baseline	External evaluation		
**Particulars**		**L3**	**L2**	**Total**
**Total number of health facilities**	**141**	**68**	**71**	**139**
**Designation of respondents**				
% facilities have an enquiry/Help desk	27.0	80.4	46.2	62.9
% facilities have Security services at all hours	28.4	53.9	51.1	52.5
% facilities has dietary facility/service for indoor patients	85.8	100.0	88.9	94.3
% Citizen charter displayed at prominent place in facilities	70.2	81.4	81.6	81.5
**Functional referral transport service available at facilities**	-			
**% facilities having ambulance/referral transport facility**	-	96.0	92.7	94.3
Yes, 108 available	-	70.5	50.9	60.4
Yes, 102 available	-	29.4	34.8	32.2
Yes, health facility ambulance available	-	59.7	39.7	49.5
Others (Mamta Van)	-	15.7	24.3	20.1

Table A2.10 Facilities where IEC display in facilities in admission/OPD						
**Particulars**	**L3**		**L2**		**Total**	
**Total number of health facilities**	**68**		**71**		**139**	
**% of facilities where IEC display in facility in admission/ OPD area**	**IEC displayed**	**In Local Language**	**IEC displayed**	**In Local Language**	**IEC displayed**	**In Local Language**
JSY entitlements (wall painting/banner etc.)	80.3	37.5	75.9	33.9	78.1	35.7
JSSK entitlements (wall painting/banner etc.)	81.2	41.4	88.1	33.9	84.8	37.6
Referral Transport Details (wall painting/banner etc.)	86.2	45.4	81.1	30.1	83.5	37.6
Complaint/suggestion box available in waiting area	84.8	8.3	81.9	15.2	83.3	11.8
A clear signage at entrance of the facility (near registration counter) to direct patients to LR	88.2	12.3	74.0	19.3	80.9	15.8

Table A2.11 Background characteristic of health providers (in percentages)			
**Particulars**	**L-3**	**L-2**	**Total**
**Total number of health providers (unweighted)**	118	77	195
**Total number of health providers (weighted)**	738	438	1176
**Designation of health provider**			
Staff Nurse	91.9	74.7	85.4
ANM	8.2	25.3	14.5
**Highest qualification**			
B.Sc. nursing	18.0	9.4	14.8
M.Sc. nursing	0.8	0.0	0.5
Diploma in nursing	71.2	77.7	73.6
Others	9.4	12.9	10.7
**Mean number of years working as nurse/ANM**	9.5	8.9	9.3
**Mean number of years posted in this facility**	5.2	5.0	5.2
**Mean number of years working in the labour room**	4.0	4.9	4.3
**Place of Residence**			
Staff quarters of the facility	21.9	29.8	24.8
Sharing staff quarter in facility	0.0	3.9	1.5
In same village/town where facility is located	45.2	44.1	44.8
In village/town other than where facility is located	29.7	22.3	26.9
Others	3.2	0.0	2.0

Table A2.12 Posting of Nurses and Service Provided (in percentages)			
**Particulars**	**L-3**	**L-2**	**Total**
**Total number of health providers (unweighted)**	118	77	195
**Total number of health providers (weighted)**	738	438	1176
**Place of posting other than labour room**			
Operation Theatre (OT)	36.9	26.7	33.1
Ward	87.4	69.4	80.7
Out Patient Care Department (OPD)	23.9	63.6	38.7
Others	43.1	41.1	42.4
**Shift duty or are on call 24 hour**			
Yes shift duty	97.2	92.9	95.6
On 24 hours call	2.8	4.6	3.5
Yes shift duty & On emergency duty	0.0	2.4	0.9

**Services provided during duty hours**			
ANC check-up	75.3	94.2	82.3
Conduct deliveries	95.9	98.8	97.0
Help doctor in caesarean deliveries	30.9	12.3	24.0
Assist doctor	56.8	34.8	48.6
Follow up of the indoor patient	83.6	83.1	83.4
General: IV drug, injection, medicine, oxygen etc.	94.7	94.6	94.6
Others	7.9	26.2	14.7

Table A2.13 Type of training received by health providers (in Percentages)

Particulars	L-3	L-2	Total
**Total number of health providers (unweighted)**	118	77	195
**Total number of health providers (weighted)**	738	438	1176
**Trainings received from state government during the last two years**			
Skilled Birth Attendant (SBA)	30.9	41.0	34.7
Navjat Shishu Suraksha Karyakram (NSSK)	39.5	40.2	39.8
Post-Partum IUCD (PPIUD)	45.0	47.8	46.0
Daksh skill labs	21.1	24.8	22.5
Others	53.4	67.0	58.5

Table A2.14 Capacity building in Care Around Birth Approach (in Percentages)

Particulars	L-3	L-2	Total
**Total number of health providers (unweighted)**	118	77	195
**Total number of health providers (weighted)**	738	438	1176
**Health providers participated in “Care around Birth” training**			
held in 2016	42.5	50.7	45.6
Held in 2017	3.2	0.0	2.0
**Average duration of training (in days)**	2.2	2.3	2.2
**Topics covered in training**			
Essential Childbirth Care and Labour monitoring	89.7	92.0	90.7
Active Management of Third Stage of Labour	91.2	100.0	94.8
Essential newborn Care and Resuscitation	93.2	100.0	96.0
newborn Vaccination	50.7	67.0	57.5
Postnatal monitoring and counselling at discharge	85.3	89.6	87.1
Others	23.3	43.8	31.8
**Percentage of health providers received orientation at the facility level in “Care around Birth” in 2016**	62.8	65.0	63.6
**Topics covered in orientations held in facility**			
Essential Childbirth Care and Labour Monitoring	94.4	96.2	95.1
Active Management of Third Stage of Labour	91.9	100.0	95.0
Essential newborn Care and Resuscitation	100.0	97.9	99.2
newborn Vaccination	62.2	72.6	66.2
Postnatal monitoring and counselling at discharge	95.7	95.9	95.8
Others	25.0	42.0	31.5

Table A2.15 Methods used for Training (in percentages)

Particulars	L-3	L-2	Total
**Total number of health providers (unweighted)**	118	77	195
**Total number of health providers (weighted)**	738	438	1176
**Methods used during the trainings**			
Demonstration	95.0	96.3	95.5
Case studies	52.0	45.5	49.5
Practice on mannequins	95.9	97.5	96.5

Table A2.15 Methods used for Training (in percentages)			
Particulars	L-3	L-2	Total
Role plays	84.2	81.9	83.4
In dyads/ working in small groups	68.0	67.2	67.7
Visualization in participatory planning writing	45.8	48.1	46.7
Any other	15.8	18.7	16.9
**Method helped in enhancing provider's knowledge and skills**			
Demonstration	57.5	53.7	56.0
Case studies	4.3	2.6	3.7
Practice on mannequins	21.9	34.9	26.8
Role plays	28.5	42.9	34.0
In dyads/ working in small groups	2.1	4.2	2.9
Visualization in participatory planning writing	1.1	4.3	2.3
Video	2.6	6.7	4.1
Others	8.5	13.0	10.2

Table A2.16 Advantages of Care around Birth training			
Particulars	L3	L2	Total
Mother care during delivery	48.3	66.2	55.1
Child care during/after delivery	35.2	39.2	36.7
Resuscitation	12.1	18.7	14.6
KMC	9.8	12.5	10.9
QI team/monitoring	8.3	8.9	8.5
AMTSL	8.2	2.6	6.1
Breast Feeding	4.9	6.5	5.5
Drying the baby	5.9	3.8	5.1
Referral	2.7	9.0	5.1
Cleanliness/hygiene	1.7	5.7	3.2
Others (Vaccination, Delivery Preparation, Registers/records, Family planning, Child check-ups, Counselling, Follow up, Staff)	18.3	19.5	18.9
**Total Health providers received CAB training**	**672**	**407**	**1079**

Table A2.17 Other activities undertaken in facility as part of the approach			
Particulars	L3	L2	Total
Registers/records	81.5	75.4	79.2
QI team/monitoring	71.9	79.7	74.8
Training	28.7	22.6	26.4
Mother care during delivery	22.4	27.7	24.4
Others (Child care during/after delivery, BMW, Medicines Cleanliness/hygiene, Counselling, Resuscitation, KMC, Referral, Follow up, Logistics/infrastructure, Breast Feeding, Vaccination)	37.2	17.2	29.6
**Total health providers interviewed**	738	438	1176

Table A2.18 Competancy in Partograph (in percentages)								
	Baseline				External evaluation			
Particulars	<50 %	51-80%	80%+	Total	<50 %	51-80%	80%+	Total
**Partograph Score**								
L3 level	65.6	22.8	11.6	224	5.4	63.4	31.2	738
L2 level	63.5	22.0	14.5	200	8.6	52.0	39.4	438
Total	64.6	22.4	13.0	424	6.6	59.2	34.3	1176
**Foetal Heart Rate**								
L3 level	65.2	0.0	34.8	224	8.6	45.9	45.5	738
L2 level	58.5	0.0	41.5	200	16.7	34.6	48.7	438
Total	62.0	0.0	38.0	424	11.6	41.7	46.7	1176

Table A2.18 Competancy in Partograph (in percentages)								
	Baseline				External evaluation			
Particulars	<50 %	51-80%	80%+	Total	<50 %	51-80%	80%+	Total
**Membrane and Amniotic Fluid**								
L3 level	71.0	0.0	29.0	224	3.1	28.8	68.1	738
L2 level	71.0	0.0	29.0	200	4.7	12.6	82.8	438
Total	71.0	0.0	29.0	424	3.7	22.7	73.6	1176
**Cervical Dilation**								
L3 level	72.8	0.0	27.2	224	53.8	8.4	37.8	738
L2 level	69.0	0.0	31.0	200	38.0	21.6	40.4	438
Total	71.0	0.0	29.0	424	47.9	13.3	38.8	1176
**Time Recording**								
L3 level	-	-	-	-	8.5	14.7	77.4	738
L2 level	-	-	-	-	10.4	23.8	65.8	438
Total	-	-	-	-	9.2	17.7	73.1	1176
**Contractions**								
L3 level	94.2	0.0	5.8	224	22.8	54.7	22.5	738
L2 level	94.0	0.0	6.0	200	22.3	58.7	19.0	438
Total	94.1	0.0	5.9	424	22.6	56.2	21.2	1176
**Interventions**								
L3 level	-	-	-	-	1.0	99.0	-	738
L2 level	-	-	-	-	14.4	85.6	-	438
Total	-	-	-	-	6.0	94.0	-	1176
**BP**								
L3 level	64.7	4.5	30.8	224	1.2	7.8	91.0	738
L2 level	60.0	3.5	36.5	200	1.2	7.8	91.0	438
Total	62.5	4.0	33.5	424	1.2	7.8	91.0	1176
**BP-Diastolilc**								
L3 level	-	-	-	-	1.2	13.1	85.7	738
L2 level	-	-	-	-	1.3	7.9	90.7	438
Total			-	-	1.2	11.2	87.6	1176
**BP-Systolic**								
L3 level	-	-	-	-	6.1	11.3	82.6	738
L2 level	-	-	-	-	5.0	8.8	86.2	438
Total					5.7	10.4	83.9	1176
**Pulse**								
L3 level	-	-	-	-	46.4	50.1	3.5	738
L2 level	-	-	-	-	50.4	49.6	0.0	438
Total	-	-	-	-	47.9	49.9	2.2	1176
**Temperature Recording**								
L3 level	-	-	-	-	6.6	5.4	88.0	738
L2 level	-	-	-	-	0.0	9.9	90.1	438
Total	-	-	-	-	4.2	7.1	88.8	1176
**Patient Details**								
L3 level	63.4	0.0	36.6	224	**15.4**	**52.3**	**32.3**	**738**
L2 level	59.0	0.0	41.0	200	**11.2**	**53.1**	**35.6**	**438**
Total	61.3	0.0	38.7	424	**13.9**	**52.6**	**33.8**	**1176**
**Clinical Endpoint**								
L3 level	60.3	0.0	39.7	224	-	-	-	-
L2 level	48.5	0.0	51.5	200	-	-	-	-
Total	54.7	0.0	45.3	424	-	-	-	-

Table A2.19 Competency in preparedness of labour room			
Particulars	L3	L2	Total
**Total number of health providers (unweighted)**	**118**	**77**	**195**
**Total number of health providers (weighted)**	**738**	**438**	**1176**
**Ensured draught free labour room by switching off fan and closing windows (S)**	85.7	83.3	84.8
**Switched on radiant warmer at least 20 minutes before expected time of delivery (S)**	97.7	98.7	98.1
**Ensured delivery room temperature is >25^0 C (S)**	96.1	90.9	94.1
**Ensured availability of components of Newborn Care Corner (SD)**			
Shoulder roll	94.3	96.5	95.1
Bag and mask (0 & 1 Size)	98.7	100	99.2
Mucous Extractor	97.9	95.0	96.8
Functional Source of Oxygen	97.3	91.0	95.0
Functional Clock with seconds hands	67.1	80.5	72.1
**Placed two clean towels under the radiant warmer maintaining sterile conditions (S)**	95.5	97.3	96.2
**Hand washing (D) (to be demonstrated using soap and water)**			
Rolled up sleeves, removed watch, bangles and rings	81.7	92.7	85.8
Rolled up the full sleeves up to elbow	84.9	86.3	85.4
Wet the hands with water from the tap or being poured by someone using mug or pitcher	91.1	98.8	94.0
Wet hands and forearms up-to the elbow	91.0	97.4	93.4
Using plain water and soap, washed parts of the hand in the following sequence: Palms and fingers and web spaces→ Back of hands → Fingers and knuckles→ Thumbs→ Finger tips and nails→ Wrists and forearms up-to elbow	100.0	97.5	99.1
Rinsed with clean water flowing from the tap or being poured by someone using mug or pitcher	97.5	96.2	97.0
Above steps in sequence	71.9	73.2	72.4
**Wore a pair of sterile gloves (S)**	98.7	98.7	98.7
**Ensured availability of Clean cord ties/ clamps and surgical blade (S)**	96.2	89.2	93.6
**Functioning of bag and mask checked (D) (to be demonstrated on bag and mask)**			
Felt pressure on the palm when the bag is squeezed	87.9	88.9	88.3
Upon squeezing enough the pop off valve opened and makes a sound	88.5	87.7	88.2
Checked that the bags re-inflates quickly when you release pressure	88.5	86.4	87.7
**The above signal functions in bold were done in sequence**	62.6	63.6	63.0

Table A2.20 Competency in providing the routine care provided to baby after birth.			
Particulars	L3	L2	Total
**Total number of health providers (unweighted)**	**118**	**77**	**195**
**Total number of health providers (weighted)**	**738**	**438**	**1176**
Called out the time of birth (S)	75.6	62.4	70.7
Delivered the baby over mother's abdomen (S)	97.8	97.6	97.7
Checked for meconium (S)	92.9	78.0	87.3
Checked whether baby is crying or breathing normally (SD)	98.4	94.7	97.0
Immediate warmth provided (SD)	85.4	78.8	82.9
Drying the baby	**95.6**	**89.0**	**93.1**
Dried newborn with a warm cloth in the following chronology. Head→ Face→ Neck→ Axilla→ Arms→ Hands→ Chest→ Abdomen→ Groin→ Legs→ Feet→ Back	97.5	100.0	98.4
Removed the wet cloth	100.0	97.6	99.1

Table A2.20 Competency in providing the routine care provided to baby after birth.			
**Particulars**	**L3**	**L2**	**Total**
Put the baby between mother's breast in skin to skin contact	98.1	91.5	95.6
**Skin to skin contact provided (SD)**	**49.3**	**57.0**	**52.2**
Place the baby between the mother's breast in a prone position	97.8	92.7	95.9
Turn the face of the baby one side	95.4	87.8	92.6
Cover the baby's head & back with a warm cloth	81.4	85.4	82.9
Cover the mother and baby with an additional blanket	51.0	62.0	55.1
**Cord clamping (SD)**	**42.3**	**53.0**	**46.3**
Delayed the clamping of cord for 1-3 min	95.9	96.2	96.0
Tied with thread in two places (2 & 5 cm. from umbilicus)	95.8	96.2	96.0
Cut umbilical cord with a clean blade	91.8	93.5	92.4
Observed for oozing of blood	58.1	64.1	60.3
Did not apply anything on stump	58.6	70.1	62.9
**Breast feeding (SD)**			
Looked for baby cues (S)	64.9	58.1	62.4
**Support the mother to initiate breast feeding**			
1. Positioning (D)<br>The whole body of baby is supported	98.2	100.0	98.9
2. The head and trunk are in one line	96.8	97.3	97.0
3. Head is not tilted	89.2	93.4	90.8
4. The body of newborn is close to the mother	96.6	97.6	96.9
**Attachment (S)**			
1. The mouth of baby is wide open	93.2	91.2	92.5
2. The upper part of areola is more visible than the lower part	90.1	76.8	85.2
3. Lower lip averted	86.8	88.8	87.5
4. Chin of baby touching the breast	78.9	75.8	77.8
**Advice on advantages of colostrum feeding reinforced (S)**	45.7	32.3	40.7
**Vitamin K1 administered within one hour of birth (S)**	**88.1**	**85.3**	**87.1**
1. Deep intramuscular, anterolateral aspect of thigh	90.8	87.9	89.7
2. Dose for vitamin K1	94.4	96.2	95.1
**The provider spoke about starting the examination around one hour of birth**	62.0	66.1	63.5
**Newborn examination**			
1. Hand washing before examination **(S)**	41.3	36.9	39.7
2. Examination started at around one hour from birth	72.6	68.8	71.1
**Measured respiratory rate (SD)**			
1. Exposed chest of the baby	68.3	61.2	65.7
2. Counted the breathes for one minute using a watch/ clock	64.6	57.1	61.8
**Weight recorded (SD) (to be demonstrated using a weighing scale)**			
1. Set the zero of scale with a cloth placed over the tray	89.6	79.4	85.8
2. Placed baby on the scale with minimum clothes, preferably naked	89.6	80.9	86.4
3. Read the measurement correctly up to the nearest 50 grams weight	78.0	71.9	75.8
4. Removed the baby from the tray, wrap the baby immediately	88.2	78.6	84.6
5. Record the weight	94.0	92.4	93.4
**Temperature recorded (SD)**			
1. Place the silver end of the bulb vertically in the middle of the armpit, under the baby's arm.	85.1	79.9	83.1
2. Gently hold the baby's arm against the body	89.2	90.7	89.7
3. Remove the thermometer after for 3-5 minutes	94.2	93.4	93.9
4. Read and record the temperature	97.7	94.6	96.6
**Head to toe examination (SD)**			
1. Assessed general appearance alertness and tone	87.0	92.3	88.9

Table A2.20 Competency in providing the routine care provided to baby after birth.			
**Particulars**	**L3**	**L2**	**Total**
2. Check for sex of the baby	89.8	75.2	84.4
**Examined head for**			
1. Fontanelles and sutures	94.7	93.6	94.3
2. Cleft lip	96.8	93.8	95.7
3. Cleft palate	97.6	95.0	96.7
Examined eyes for redness and discharge	93.7	86.1	90.9
Examined skin for jaundice, pallor and cyanosis	89.8	86.3	88.5
Inspected skin for sores or breaks	70.7	78.5	73.6
Examined for muscle tone	84.9	88.6	86.3
Palpated abdomen and liver	88.3	89.0	88.5
**Examined extremities, skeletal system**			
1. Club hands	94.6	96.2	95.2
2. Club feet	91.6	93.8	92.4
Other congenital anomalies like			
1. Spina bifida	82.7	81.1	82.1
**The above signal functions in bold were done in sequence**	36.2	42.7	38.6

Table A2.21 Competency in providing Resuscitation			
**Particulars**	**L3**	**L2**	**Total**
**Total number of health providers (unweighted)**	**118**	**77**	**195**
**Total number of health providers (weighted)**	**738**	**438**	**1176**
Called out time of birth (S)	67.3	60.4	64.7
Checked for crying	98.4	97.6	98.1
(Baby not crying) Suction immediately: mouth first and then nostrils	92.1	92.5	92.2
Dried baby thoroughly (D)	94.1	93.8	94.0
**Assessed for breathing (SD)**			
1. Checked whether the baby was crying or breathing normally by observing chest movements or listening to breathing sounds	96.4	94.9	95.8
**Baby not crying/ breathing**	96.5	97.4	96.8
**Quickly placed two forceps/ ties (anywhere on the cord) and cut (SD)**	99.4	98.8	99.2
**Moved baby to the newborn care corner (SD)**<br>**Positioning (D)**			
**1.** Placed shoulder roll	98.0	97.6	97.8
2.			
**3.** Positioned the head in sniffing position	93.6	94.9	94.1
**Suction (D)**			
**1.** Suctioned first mouth then both nostrils	97.7	92.6	95.8
**Stimulation (rubbing at back and flickering at soles) (D)**	92.6	85.0	89.7
**Repositioning (D)**			
**1.** Placed shoulder roll	87.6	86.9	87.4
**2.** Positioned the head in sniffing position	87.5	86.6	87.2
**Checked if the selected mask in the bag and mask assembly covered chin, mouth & nose(D)**	95.2	94.0	94.8
**Applied face mask firmly covering chin, mouth & nose(D)**	98.3	97.7	98.1
**Ventilate through bag and mask (D)**			
**1.** Ventilation started	97.7	96.0	97.0
**2.** Checked for the chest rise	92.1	90.0	91.3
3. For each ventilation cycle spoke "Breathe two-three"	98.6	98.8	98.7
**4.** Ventilated for thirty seconds	83.4	88.9	85.5
**Assessed the baby (D)**Assessed for breathing	92.3	88.5	90.9
**Baby still not crying/ breathing (prompt to the provider)**	85.2	87.5	86.0
**Called for help (S)** Asked to arrange for vehicle for transportation and inform the doctor	88.3	88.5	88.4

Table A2.21 Competency in providing Resuscitation			
**Particulars**	**L3**	**L2**	**Total**
**Attached oxygen to the bag and mask assembly (D)**	93.7	87.1	91.3
**Continued bag and mask ventilation(D)**	87.5	87.3	87.4
**Checked heart rate (D)**			
**i) Heart rate below 100 beats per minute (prompt to the provider)**	74.5	74.5	74.5
**Continued bag and mask ventilation with oxygen(D)**	90.9	87.2	89.5
**Checked heart rate after next 30 seconds**	76.6	85.0	79.8
**After one minute from the start of bag and mask ventilation the baby started breathing and maintained 100 beats per minute (prompt to the provider)**	73.5	74.5	73.8
**Gradually stopped ventilation (D)**	79.7	77.1	78.7
**Gave baby to the mother(S)**	82.8	74.9	79.9
**Monitored the baby with the mother (S)**			
1. Breathing: rate and regularity (normal: 40-60 per minute & regular)	86.4	81.8	84.7
2. Heart Rate: rate (normal: >100 per minute)	94.6	76.8	87.9
3. Temperature: within the normal range (normal: 36.5-37.4^{0}C)	95.8	92.4	94.6
4. Color (normal: pink /// abnormal: blue or pale)	98.7	92.3	96.4
**All above signal functions both bold and non-bold were done in sequence**	33.9	48.5	39.3

Table A2.22 Competency in Counselling on danger signs in newborns and when to return			
**Particulars**	**L3**	**L2**	**Total**
**Total number of health providers (unweighted)**	**118**	**77**	**195**
**Total number of health providers (weighted)**	**738**	**438**	**1176**
**When to return (S)**			
Breastfeeding or drinking poorly	92.7	95.0	93.6
Convulsions	61.3	70.9	64.9
Fast breathing	79.5	79.7	79.6
Difficult breathing	80.6	85.9	82.6
Develops a fever or feels cold to touch	92.1	86.3	90.0
Diarrhea	69.7	75.0	71.7
Blood in stools	41.3	34.7	38.8
**Loss of consciousness**	24.5	39.2	29.9

Table A2.23 Competency in Counselling on newborn at discharge			
**Particulars**	**L3**	**L2**	**Total**
**Total number of health providers (unweighted)**	**118**	**77**	**195**
**Total number of health providers (weighted)**	**738**	**438**	**1176**
**Counselling at discharge done (S)**			
1. Instructed the mothers about the benefits of breast-feeding	96.2	90	93.9
2. Exclusive breast feeding for 6 months not even water	86.1	90.1	87.6
**3.** Maintenance of hygiene	82.0	88.8	84.5
4. Do not apply anything on the cord and keep the cord and umbilicus dry	52.2	66.2	57.4
**5.** Counsel about immunization of baby at age of 6, 10 & 14 weeks	80.3	87.8	83.1

Table A2.24 Competency in newborn Vaccination			
**Particulars**	**L3**	**L2**	**Total**
**Total number of health providers (unweighted)**	**118**	**77**	**195**
**Total number of health providers (weighted)**	**738**	**438**	**1176**
**Vaccination done (S)**			
1. Administered BCG intradermal left arm	94.9	94.1	94.6
**2.** Administered birth dose of Hepatitis B (IM)- within 24 hours	94.3	91.7	93.3
3. Administered zero dose OPV (Oral)	95.7	90.6	93.8

Table A2.25 Competency scored regarding postnatal monitoring			
**Particulars**	**L-3**	**L-2**	**Total**
**Total number of health providers (unweighted)**	**118**	**77**	**195**
**Total number of health providers (weighted)**	**738**	**438**	**1176**
**Meaning of Fourth stage of labour**			
It is the stage from delivery of placenta up to two hours	61.2	59.8	60.7
It is the stage from delivery of placenta up to four hours	24.2	16.5	21.3
It is the stage from delivery of baby up to two hours	10.9	13.7	12.0
It is the stage from delivery of baby to delivery of placenta	3.7	7.4	5.1
Others	0.0	2.6	1.0
**Savita gave birth to a healthy baby girl one hour ago. You managed the third stage of labour actively, the placenta was complete, and she had no perineal or vaginal lacerations. Timing for monitoring her uterus and vaginal bleeding during the second hour after delivery of the placenta**			
Every 15 minutes	59.4	69.3	63.1
Every 30 minutes	37.6	26.9	33.6
Every 10 minutes	1.9	1.1	1.6
Every 60 minutes	1.1	2.7	1.7
**Timings for counselling and suporting to the women regarding breast feed to newborn**			
As early as possible within 1 hour of birth	98.1	98.8	98.4
Only After the woman has been shifted to the ward	0.0	1.2	.5
Within 2 days of delivery	1.9	0.0	1.2
**Indication for the uterus if it is not firm even after one hour of delivery of placenta**			
Uterus may have placental remnants & Uterine atony	61.2	49.6	56.9
Uterine atony	21.2	19.1	20.4
Uterus may have placental remnants	11.0	20.3	14.4
Uterus may have placental remnants & Lacerations in genital tract	5.0	9.9	6.8
Lacerations in genital tract	1.7	0.0	1.0
Uterus may have placental remnants, Uterine atony& Lacerations in genital tract	0.0	1.1	0.4
**Counselling to be given during post-partum period**			
Exclusive breastfeeding, including colostrum feeding	6.4	11.0	8.1
To take adequate rest, sleep	1.4	4.7	2.6
Maintain hygiene including perineal hygiene, and washing her hands before handling the baby	7.9	4.0	6.4
Family planning method	6.3	6.5	6.3
All of the above	83.7	79.4	82.1
Others	7.8	8.2	8.0
**The condition(s) where the birth companion should be asked to stay with the mother and newborn and call for help(s)**			
Mother complains of severe headache	65.3	74.5	68.7
Mother feeds baby	52.1	53.2	52.5

Table A2.25 Competency scored regarding postnatal monitoring			
**Particulars**	**L-3**	**L-2**	**Total**
Every time mother passes urine	46.5	53.9	49.2
Only when it is time for discharge	21.2	24.2	22.3
Other	8.1	6.9	7.7
**Time where thorough examination of a newborn should be conducted**			
At 30 minutes	59.1	43.8	53.4
At one hour	31.6	46.0	37.0
At one and half hour	1.0	3.8	2.0
At two hour	5.3	5.4	5.3
**Frequency of monitoring a new -born for breathing and temperature within one hour of birth**			
Every 15 minutes	72.1	79.8	75.0
Every 30 minutes	22.2	13.0	18.7
Every 10 minutes	5.8	7.2	6.3
**Number of times a healthy newborn should take breastfeeds within 24 hours**			
At least 10 times	62.7	46.9	56.8
At least 8 times	26.5	44.8	33.3
At least 9 times	6.3	2.5	4.9
At least 7 times	4.4	5.9	5.0
**The normal range of temperature of a newborn**			
36.5-37.4 C	65.6	78.8	70.6
35.5-36.4 C	12.4	13.9	12.9
34.5-35.4 C	1.3	2.6	1.7
37.5-38.4 C	15.4	3.4	10.9
Others	1.0	1.2	1.1

Table A2.26 Competencies in Infection Prevention			
**Particulars**	**L-3**	**L-2**	**Total**
**Total number of health providers (unweighted)**	**118**	**77**	**195**
**Total number of health providers (weighted)**	**738**	**438**	**1176**
**Step taken immediately in labour ward for Contaminated instruments (%)**			
Soaked in 0.5% chlorine solution for 10 minutes	78.0	79.2	78.5
Soaked in 0.5% chlorine solution for 30 minute	13.5	8.4	11.6
Washed with soap and water and soaked in 0.5% chlorine solution for 10 minutes	3.7	9.9	6.0
Washed with soap and water and soaked in 0.5% chlorine solution for 30 minutes	3.9	2.4	3.4
**Most appropriate method and sequence of processing soiled instruments, used surgical gloves and other usable items**			
Decontamination→ Cleaning→ Sterilization/ High Level Disinfection	78.0	73.3	76.2
Cleaning→ Decontamination→ Sterilization/ High Level Disinfection	11.6	15.0	12.9
Sterilization/High Level Disinfection →Cleaning→ Decontamination	5.2	4.0	4.7
Decontamination→ Sterilization/High Level Disinfection → Cleaning	5.2	7.7	6.1
**Steps needs to be taken for disposal of the placenta**			
Use gloves while handling the placenta	11.8	7.1	10.0
Put the placenta into a leak-proof bag containing bleach	6.1	7.4	6.6
Dispose the placenta in the yellow bag	34.2	17.1	27.9
All of the above	60.4	74.4	65.7
**Preventive measures taken while taking blood samples or establishing an intravenous line (%)**			
It is not necessary to wear gloves	6.0	3.6	5.1

Table A2.26 Competencies in Infection Prevention			
**Particulars**	**L-3**	**L-2**	**Total**
Sterile surgical gloves should be worn	61.3	54.1	58.6
New examination gloves should be worn	26.1	37.6	30.4
Double gloves should be worn	6.6	4.7	5.9
**Reasons for decontaminating surgical instruments by soaking in 0.5% chlorine solution**			
Completely kills all microorganisms, including bacterial	83.3	81.3	82.6
Rapidly kills viruses such as HIV and hepatitis B	50.6	53.9	51.8
Does not kill viruses such as HIV and Hepatitis B	5.5	6.0	5.7
Does not need to be done if instruments are thoroughly washed and rinsed	1.4	0.0	0.9
**Particulars**	**L-3**	**L-2**	**Total**
**Frequency of washing the hands**			
Before and after examining a patient/client	23.2	15.7	20.4
Before and after using gloves	5.7	7.4	6.4
After contact with blood or other body fluids, or soiled instruments	8.4	3.5	6.6
All of the above	75.0	81.9	77.6
**Things needs to be done for disposal of placenta**			
Dispose the placenta in the yellow bag	34.2	17.1	27.9
Use gloves while handling the placenta	11.8	7.1	10.0
Put the placenta into a leak-proof bag containing bleach	6.1	7.4	6.6
All of the above	60.4	74.4	65.7
**Colour of bin used after conducting a delivery for disposing placenta, oxytocin syringe and needle respectively**			
Yellow bin, red bin and white puncture proof container	73.1	67.1	70.9
White puncture proof container and Yellow bin	13.0	15.3	13.9
Yellow bin and red bin<br>White puncture proof container	8.7	6.5	7.9
Yellow bin and red bin	4.0	6.5	4.9
**Categorization of medical waste/contaminated waste which should be segregated in the Red bag**			
Used mutilated catheters I.V bottles and tubes, disinfected plastic gloves, other plastic material	93.1	77.5	87.3
Tubing like I.V. drip sets and different types of Catheters and tubes	48.0	58.4	51.9
Used swabs/ gauze/ bandage, other items (surgical waste) contaminated with blood	12.7	17.2	14.4
General waste from food	0.6	2.4	1.3
**Procedure followed for preparing 0.5% Chlorine solution for decontamination of used (infectious) items**			
Addition of 3 table spoon bleaching powder paste to 1 litre water	67.8	57.8	64.0
Addition of 3 teaspoon bleaching powder paste to 1 litre water and same ratio for larger volume	21.0	19.8	20.6
Addition of 1 teaspoon bleaching powder to 1 litre water and same ratio for larger volumes	5.4	13.5	8.4
Addition of 3 teaspoon Bleaching powder to 10 litre water	1.7	1.2	1.5
Others	1.8	4.4	2.8
**Timing for preparing fresh 0.5% Chlorine solution for decontamination of used (infectious) items**			
Every day	92.1	92.9	92.4
Once in two days	4.1	2.5	3.5
Every week	1.7	0.0	1.1
Once in three days	0.9	1.2	1.0

Table A2.27 Mechanism to ensure continuity in long term				
**Particulars**		**L3**	**L2**	**Total**
**% providers responded on ways to ensure its continuity in long term**		98.6	100.0	99.1
QI team/monitoring	Should encourage other people to do the process. Training to new staff thru documentation. Random check-ups of all the requirements. Continuous monitoring of the process done the staff and working with the staff and demonstrating the process. Discussing and solving problems. Clearing doubts.	58.5	63.5	60.4
Training	Regular trainings should be arranged for juniors and new comers or those who did not attend the training. Refresher trainings to be arranged	46.7	38.1	43.5
AMTSL	Follow all the steps and do all the required preparation beforehand and keep things ready for delivery and newborn care.	16.4	20.2	17.8
Staff	All members should be actively involved. Regular trainings for the newcomers. All the shortfalls should be taken care of.	11.5	10.4	11.1
Registers/records	Systematically maintaining the records and registers. Should be checked in between for completeness.	3.3	7.5	4.9
Others (IEC materials, Medicines, Mother care during delivery, Cleanliness/hygiene, Logistics/infrastructure, Follow up, Delivery Preparation, Counselling, Child care during/after delivery)	Vriddhi project should continue. Keep improving quality. Proper arrangement is required for good quality work. All posters should be displayed properly and also used as guidelines. Informing seniors about the stock so that medicine made available before it finishes. Inform about the medicine to the next shift staff. Proper care of the mother as taught in the training and nice behaviour with the mother. Cleanliness should be supervised. Following all the practices learnt for sterilization of the equipment. Proper care of instruments. Should be repaired at the earliest if anything is damaged or not functional. Maintaining the equipment. Inform immediately to the in-charge about supply or damage. Quarters for staff nurse on night duty. All routine vital check-ups to be done regularly. 7 trays should be ready 24 hours. Following the steps learnt. Tell people about proper breastfeeding practices, nutrition and danger signs, and Also counsel about PPIUCD. Taking care of the baby as per the process learnt	11.2	13.6	12.1
**Total health providers interviewed**		**738**	**438**	**1176**

Table A2.28 Skills /methods not used by providers with reasons				
**Particulars**		**L3**	**L2**	**Total**
**Health providers unable to use a skill or methods of service delivery learnt while providing services**		36.0	39.2	37.2
**skill or methods of service delivery unable to use while providing services**				
Mother care during delivery	Maintaining Privacy, Curtains/screens between labour tables, care for high risk patient, following all steps of delivery	28.5	40.7	33.2
KMC	KMC service, Not able to do KMC in labour room	23.0	9.6	17.8
AMTSL	Maintaining room temperature, Filling partograph, completing and properly filling partograph	15.2	13.4	14.5
Breast Feeding	Initiate within labour room, initiate immediately within an hour	8.6	3.1	6.4

Table A2.28 Skills /methods not used by providers with reasons				
**Particulars**		**L3**	**L2**	**Total**
Logistics/infrastructure	Labour table with partition,	2.8	11.9	6.3
Cleanliness/hygiene	Cleaning with bleaching solution	5.3	2.8	4.4
Family planning	Convince about PPIUCD	4.1	2.8	3.6
Child care during/after delivery	Use of Radiant Warmer	2.8	3.1	2.9
Resuscitation	Resuscitation in some cases	2.8	2.8	2.8
Registers/records	PNC and discharge registers	4.3	0.0	2.7
Training	No training to all staff	2.2	3.1	2.5
Others (Delivery Preparation, Staff, Referral, Counselling, Drying the baby, Medicines)	Managing black fever, provide service in ward room, vital check-ups, HIV/AIDS testing, giving magnesium sulphate, BP checking. Too much workload hence cannot follow all steps and processes. Counselling about nutrition for self and child. Meconium care, drying the baby properly. Some cases cannot handle and have to refer. Magnesium injection	17.8	19.3	18.2
**Reasons for unable to use skill or methods of service delivery while providing services**				
Lack of staff/ Lady Doctor	Not able to provide all the services properly due to shortage of staff. Lady doctor is not available hence sometimes patient needs to be referred	42.4	41.3	41.8
Area/room is small	Labour room is very small. It is not possible to keep all required things inside the room. KMC/newborn corner is not separate or is small so not able to maintain privacy	18.5	23.3	20.3
Need more training/not confident	Not confident in some of the things to do alone. Have not received training.	12.2	8.7	10.8
Patient/mother do not understand/ need more counselling	Patient and their relatives do not understand/cooperate hence more time is spent in counselling/convincing them	11.4	8.7	10.6
More workload/no time/busy	There are so many patients that we do not have sufficient time for each. Cannot even fill the records properly. Cannot wash hands also properly.	9.6	9.9	9.7
Damaged/non-functional equipments	Machines are damaged or defective and not repaired mostly the radiant warmer	11.8	3.5	8.6
Facility not available 24 hours	Many times ambulance is not available 24 hrs and patient cannot be brought	1.8	18.6	8.1
Lack of beds/Labour table	Sometimes two women have to share beds due to lack of sufficient beds, 4 labour tables are put in a small place and cannot maintain privacy. Due to lack of required number of labour tables delivery is to be done on the floor.	8.1	2.9	6.1
Lack of instruments	Not having KMC sets/magnesium sulphate	5.5	7.0	6.1
Depends on patient condition	Patient come at very advanced stage and we are not able to follow all the steps	3.0	2.9	2.9
**Total health providers unable to use any skill or methods of service delivery while providing services that was learnt**		**271**	**172**	**443**

Table A2.29 Suggestions for "care around birth"				
**Particulars**		**L3**	**L2**	**Total**
**No suggestions**		**11.2**	**5.9**	**9.2**
Staff	Shortfall should be completed. All staff should be trained. Need lady doctor, paediatrician, and gynaecologist. Doctor for 24 hours.	40.7	45.4	42.4
Training	CAB training every year. Refresher training for already trained. Training for new comers. More practice for what is learnt in the training. Practical training. Training for all staff including ASHA, sahiya.	31.4	42.0	35.3
Logistics/infrastructure	Electricity should be available 24 hours, big and tall curtains in the labour room, labour room should be big, sufficient labour tables, PNC room should be there, Full time ambulance, KMC and newborn corner, attached toilet with labour room, canteen for eatables for patient and relatives. More beds blood bank facility	18.2	21.3	19.3
AMTSL	Proper maintenance of the equipment like spot light, electric suction machine, oxygen cylinder, thermometer.	11.0	10.8	10.9
Others (QI team/monitoring, Child care during/after delivery, Cleanliness/hygiene, Counselling, Delivery Preparation, Mother care during delivery, KMC, Registers/records, Medicines, BMW, Family planning)	Patient's relatives should maintain silence in the facility, ASHA should teach mother beforehand for early initiation of breast feeding. Regular and continuous monitoring should be there, QI team should provide all the support in work and process as they are doing now. More quantity of towels, avail all instruments to avoid referral, functional room heater, temperature should be maintained. Patient relatives to be more attentive to cleanliness issues, More counselling during ANC period, Counselling for low birth weight babies. ANC counselling should be done, Clean clothes for woman who come for delivery. Napkins should be sufficient for mother. Facility should be inside labour room. All the registers should be available and properly checked. All related medicine should be always available. Plastic yellow bags should be there for placenta. Proper training for counselling and service of PPIUCD	25.9	28.8	26.9
**Total**		**738**	**438**	**1176**

Table A2.30: Health Facility Profile				
	**Baseline**	**External evaluation**		
**Particulars**		**L3**	**L2**	**Total**
**Total number of health facilities**	**141**	**68**	**71**	**139**
% facilities have a New Born Stabilization Unit (NBSU)	22.7	42.3	27.0	34.5
% facilities in which NBSU is sanctioned	-	7.9	0.0	3.8
% facilities in which NBSU is functional	8.5	34.3	13.9	23.9
% facilities have a Special newborn Care Unit (SNCU)	9.2	43.7	7.1	25.0
% facilities in which SNCU is functional	9.9	39.4	3.3	20.9
% facilities have functional Blood Bank (blood has been received/given in the past three months)	16.3	55.4	0.0	27.0
Yes, but not functional	0.7	0.0	3.5	1.8
% facilities have functional Blood storage unit (blood is stored /given in the past three months)	17.0	59.3	3.5	30.8
Yes, but not functional	1.4	8.3	0.0	4.1

Table A2.31: Availability of trays and its items (Percentages)				
	Baseline	External evaluation		
Particulars		L3	L2	Total
**Total number of health facilities**	**141**	**68**	**71**	**139**
**Delivery tray**				
All items available	41.8	88.1	84.1	86.0
Tray available	66.0	96.0	100.0	98.0
All items in tray	5.7	15.7	37.4	26.8
**Medicine tray**				
All items available	11.3	50.1	41.3	45.6
Tray available	51.1	100.0	94.4	97.1
All items in tray	2.1	11.6	11.6	11.6
**PPIUCD tray**				
All items available	29.1	54.4	67.0	90.5
Tray available	56.0	91.9	96.7	94.4
All items in tray	3.5	3.9	3.8	3.9

Table A2.32: "Most significant change" in service delivery, labour room amenities and infrastructure after implementation of CAB approach				
Particulars		L3	L2	Total
Mother care during delivery	Managing PPH, Uterine massage, respectful behavior towards the mother	54.5	53.4	54.1
AMTSL	Filling and reading partograph, managing eclampsia, referrals for CS	49.0	61.9	53.8
KMC	KMC corner is provided with clothing and proper privacy	31.3	27.4	29.8
Child care during/after delivery	Putting the baby on mothers abdomen immediately after birth	28.7	18.3	24.8
Training	Time to time training with demonstration by the seniors and discussion of the problems to solve and train other new/untrained staff	24.3	23.3	23.9
Logistics/infrastructure	Availability of ambulance service. Proper arrangement of labour room, labour tables, partitions, KMC, newborn corner. Availability of instruments/ equipment and repairing of the damaged equipment's	18.3	26.2	21.3
Cleanliness/hygiene	Improved cleaning practices, use of solution, sterilization of equipment's, proper disposal of used material	21.1	17.9	19.9
Resuscitation	Proper technique of resuscitation reducing referrals and deaths	15.0	24.3	18.4
Breast Feeding	Initiation of breast feeding immediately, if possible on labour table itself. Proper positioning of the mother and child while breast feeding	16.7	8.2	13.5
QI team/monitoring	improved quality of work done and less time spent	10.8	9.5	10.3
IEC materials	Posters, chards are displayed at proper places to improve awareness among the patient and service delivery	8.1	11.1	9.2
Registers/records	Regular maintenance of the records and registers and utilization of the data	8.1	8.7	8.4
BMW	Maintaining colour codes for disposing of garbage materials	7.2	9.3	8.0
Medicines	Availability of all related medicines and shortage is managed faster	9.2	3.9	7.2

Table A2.32: "Most significant change" in service delivery, labour room amenities and infrastructure after implementation of CAB approach				
**Particulars**		**L3**	**L2**	**Total**
Counselling	Counselling of mother regarding breastfeeding techniques, proper nutrition, and danger signs for self and child to come back to facility	6.8	2.3	5.1
Others (Delivery Preparation, Family planning, Vaccination, Referral, Drying the baby, Child check-ups, Staff)	Vital check-ups like BP, FHR, Contractions, Pulse, Preparation of the labour room, instruments, equipment. Counselling of mother for FP and use of PPIUCD. Giving vitamin K1 immediately after breastfeeding initiation and all the birth doses before discharge. Reduced referrals due to proper managing cases. Proper cleaning of meconium and drying the baby. Vital check-ups of the child every 15 minutes for at least 2 hours. Changed and improved behaviour of staff towards other staff and patients,	14.8	21.3	17
**Total health providers interviewed**		**738**	**438**	**1176**

Table A2.33: Knowledge regarding Active Mamagemnt of Third satge of labour			
**Particulars**	**L-3**	**L-2**	**Total**
**Total number of health providers (unweighted)**	**118**	**77**	**195**
**Total number of health providers (weighted)**	**738**	**438**	**1176**
**Meaning of third stage of labour**			
Delivery of baby to delivery of placenta	85.8	73.6	81.2
Full dilatation of cervix to delivery of baby	5.9	9.8	7.3
Delivery of placenta to two hours after	4.7	4.8	4.8
Initiation of true labour pain to full dilatation of cervix	3.6	10.5	6.2
Others	0.0	1.3	0.5
**Critical component(s) of Active Management of Third stage of Labour (AMTSL)**			
Administration of Uterotonic	88.8	81.5	86.1
Inspection of the placenta	46.2	46.7	46.4
Delayed cord cutting	28.2	26.2	27.5
Administration of Antibiotic	1.6	1.3	1.5
Others	0.0	1.2	0.5
**Timing of administering the uterotonic as part of AMTSL**			
Within one minute of birth of baby	92.2	85.0	89.5
Within 1 min of delivery of placenta	2.7	7.3	4.4
Just before delivery of baby	4.0	6.4	4.9
Others	0.0	1.3	0.5
**Drug of choice as a uterotonic for use in AMTSL in a facility**			
Injection Oxytocin	94.8	87.0	91.9
Tablet Misoprostol	1.1	0.0	0.7
Both Injection Methergine & Injection Oxytocin	0.6	2.7	1.4
Others	2.3	9.1	4.8
**Ideal condition for storing oxytocin**			
In a refrigerator	89.9	85.4	88.2
In the Freezer compartment of the refrigerator	5.8	13.2	8.6
In an open kidney dish in the delivery room	2.3	0.0	1.4
In a drawer in the Nurse's office, away from light	1.2	1.3	1.2
**Steps taken if placenta is undelivered even after 30 minutes of oxytocin administration, controlled cord traction and uterus has contracted**			
The woman to be referred to a facility with emergency obstetric services (FRU)	74.6	79.9	76.6
Stop controlled cord traction and wait for signs of placental separation	31.7	19.6	27.2
Controlled cord traction and fundal pressure should be attempted	19.5	15.0	17.8

Table A2.33: Knowledge regarding Active Mamagemnt of Third satge of labour			
Particulars	L-3	L-2	Total
More aggressive controlled cord traction should be attempted	9.2	11.4	10.0
Others	1.3	1.1	1.2
**Steps done before performing AMTSL**			
Assess if baby requires resuscitation	3.4	2.5	3.1
Gently palpate the woman's abdomen to rule out the presence of another baby	51.2	51.0	51.1
Dry the baby and give skin-to-skin contact	2.7	5.3	3.7
All of the above	39.6	40.2	39.8
Others	1.7	0.0	1.0
**Practices done for preventing AMTSL during problems of delivery**			
Post-partum hemorrhage	96.9	95.1	96.2
Sepsis	12.8	16.1	14.0
Obstructed Labour	6.8	2.7	5.2
Eclampsia	2.4	2.5	2.4
**Step for ruling out before administering uterotonic (misoprostol, oxytocin) drug**			
Pulsation of the umbilical cord	3.0	6.2	4.2
Uterine contractedness	16.3	12.6	14.9
The presence of another baby	88.5	85.2	87.3
Signs of placenta separation (e.g., lengthening of the cord)	7.7	9.6	8.4
**Common causes of post- partum haemorrhage**			
Retained placenta	76.1	86.6	80.0
Uterine atony	70.1	56.8	65.1
Genital lacerations	67.0	55.2	62.6
Prolonged Labour	30.9	30.2	30.6

First stage of labour

Table A3.1: Observations made during the 1st stage of labour			
Particulars	L3	L2	Total
**Total number of beneficiaries observed (un-weighted)**	**303**	**96**	**399**
**Total number of beneficiaries observed (weighted)**	**115961**	**51152**	**167113**
Draught free environment in labour room ensured by switching off fans and closing windows	100.0	100.0	100.0
Radiant warmer switched on for receiving the baby	98.2	99.1	98.5
Two clean towels placed under the radiant warmer maintaining sterile conditions	93.9	97.4	95.0
Hand washing done as per protocol (observation)	97.0	95.1	96.4
Pair of sterile gloves worn by the service providers	99.6	100.0	99.7
Service provider checked the functioning of bag and mask	92.8	88.4	91.4
**Partograph use during delivery**			
i)Partograph used	76.5	69.9	74.5
ii) Filled as per stage of delivery	76.5	69.9	74.5
iii) Filled for all parameters relevant to stage of delivery	76.5	69.0	74.2
Delivery tray kept ready	99.3	98.6	99.1
newborn tray kept ready	88.0	95.9	90.4

Second stage of labour

Table A3.2: Observations made during the 2nd stage of labour			
**Particulars**	**L3**	**L2**	**Total**
**Total number of beneficiaries observed (unweighted)**	303	96	399
**Total number of beneficiaries observed (weighted)**	115961	51152	167113
**Partograph use during delivery**			
i) Partograph used	76.5	70.4	74.6
ii) Filled as per stage of delivery	75.7	70.4	74.1
iii) Filled for all parameters relevant to stage of delivery	75.7	70.4	74.1
**Baby delivered over mother's abdomen and skin to skin contact initiated**	97.3	95.1	96.6
**Time of birth called out**	97.0	98.6	97.5
**newborn assessed for meconium and cry at birth**	98.3	96.5	97.8
**Baby dried from head to toe as per guidelines**	98.0	99.1	98.3
**Total number of newborn who required resuscitation (unweighted)**	9	5	14
**Total number of newborn who required resuscitation (weighted)**	3609	2529	6138
**Steps done for resuscitation**			
Suction done immediately if baby did not cry at birth (mouth first then nose)	88.3	100.0	93.1
Baby is shifted to the newborn care corner	88.3	100.0	93.1
Baby positioned in the newborn care corner with shoulder roll and suction done	76.5	100.0	86.2
Baby stimulated (rubbing at back and flickering at soles)	100.0	100.0	100.0
Baby repositioned for suction	100.0	70.7	87.9
Bag and mask applied correctly for resuscitation	82.1	100.0	89.5
Resuscitation initiated within the first thirty seconds of birth	93.8	100.0	96.4
Resuscitation done as per guidelines (chest rise visible, "breathe two three" spoken for each cycle, initial ventilation continued for 30 seconds)	82.1	70.7	77.4
Called for help if baby did not initiate breathing after the first cycle	93.8	53.0	77.0
Oxygen tube attached to bag and mask	93.8	53.0	77.0
Resuscitation re-initiated as per protocol for the second cycle of 30 seconds	93.8	70.7	84.3
Monitored the heart rate as per protocol during the course of resuscitation	93.8	70.7	84.3
If after one minute from the start of bag and mask ventilation the baby started breathing and maintained 100 beats per minute then resuscitation stopped as per protocol	93.8	53.0	77.0
If after one minute of resuscitation baby does not breathe spontaneously then referral done to higher facility as per protocol	82.1	35.3	62.9

Active Management of Third stage of labour

Table A3.3 Observations made during the 3rd stage of labour			
**Particulars**	**L3**	**L2**	**Total**
**Total number of beneficiaries observed (unweighted)**	303	96	399
**Total number of beneficiaries observed (weighted)**	115961	51152	167113
Prefilled syringe with Uterotonic (Oxytocin) kept ready as soon as the cervix is completely dilated	99.6	99.1	99.5
Injection Oxytocin administered	99.6	99.1	99.5

Oxytocin administered within a minute of delivery	99.6	97.7	99.0
Cord cutting time within 1 to 3 minutes	98.9	99.1	98.9
Placenta delivered by controlled cord traction (CCT)	99.2	99.1	99.2
Examines the placenta completely as per protocol	73.5	78.2	75.0
Safe disposal of placenta done	99.3	97.8	98.8
Places instruments in 0.5% chlorine solution for 10 minutes for Decontamination	95.2	98.7	96.3

Postnatal monitoring first 2 hours after delivery

Table A3.4 Observations made during the 4th stage of labour

Particulars	L3	L2	Total
**Total number of beneficiaries observed (un-weighted)**	303	96	399
**Total number of beneficiaries observed (weighted)**	115961	51152	167113
Mother and her newborn are not separated unless required	95.7	94.6	95.3
Mother kept warm and comfortable	98.5	97.7	98.3
Baby is maintained in skin to skin contact with the mother	84.9	87.2	85.6
Breast feeding initiated	95.0	96.8	95.5
Injection Vit K1 administered to the baby	96.3	98.2	96.9
Baby assessed for proper breast feeding (positioning and attachment)	75.3	90.9	80.1
Post-natal vitals for mother are recorded every 30 min. after birth	70.1	86.5	75.1
Post-natal vitals for newborn are recorded 30 min. after birth	67.3	85.7	72.9
**Newborn examination**			
Hand washing done before examination	85.0	86.0	85.3
Baby weighed	99.6	97.6	99.0
Temperature recorded	87.5	89.4	88.1
Respiratory rate recorded	87.2	86.8	87.1
Baby examined from head to toe	91.9	88.8	91.0

Client perspective

Table A3.5 Profile of beneficiaries

Particulars	L-3	L-2	Total
**Total number of beneficiaries (unweighted)**	**230**	**94**	**392**
**Total number of beneficiaries (weighted)**	**113846**	**50259**	**164105**
Average age of beneficiary (in years)	24.3	24.6	24.4
**Religion**			
Hindu	73.3	67.7	71.6
Muslim	17.4	24.3	19.5
Christian	3.0	6.2	4.0
Sikhs	6.0	0	4.1
Others	0.3	1.8	0.8
**Caste**			
Other backward class	44.6	45.5	44.9
Scheduled caste	26.0	18.7	23.8
General	19.1	19.6	19.2
Scheduled tribe	10.3	14.7	11.7
Others	0	1.5	0.5
**Main occupation**			
Housewife	86.5	85.0	86.1
Cultivator (agriculture)	7.1	7.6	7.3
Daily wage earner (labour)	3.6	5.0	4.0
Others	2.8	2.4	2.7
**Place of Residence with respect to location of health facility**			

Resident of same village/town	43.5	45.2	44.0
From another village/town	56.5	54.8	56.0

Table A3.6 Location and services availed in health facility

Particulars	L-3	L-2	Total
**Total number of beneficiaries (unweighted)**	230	94	392
**Total number of beneficiaries (weighted)**	113846	50259	164105
Average distance of health facility from village/ town (in Kms)	20.3	12.2	17.9
Percent beneficiaries used ambulance services (102 or 108) to reach health facility	49.2	69.4	55.4
**Reasons for not using ambulance services**			
No need	21.1	6.4	17.6
Called but did not come	1.3	2.1	1.5
Called but did not come in time	1.0	4.3	1.8
Do not know about ambulance services	8.4	2.1	6.9
Others	21.1	14.9	19.6
**% of beneficiaries who**			
Used ambulance and it was free	42.3	56.4	45.7
Used ambulance and average expense incurred (in Rs.)	359.3	325.0	342.6
Did not use ambulance and it was free	18.8	10.6	16.8
Did not use ambulance and average expense incurred (in Rupees)	309.4	356.9	319.1
**Average amount paid towards transport (in Rs.)**	316.4	344.9	323.8
**Person who accompanied beneficiary to facility**			
Husband	85.4	73.0	81.6
Mother in law/mother	71.9	78.0	73.8
ASHA	42.8	50.0	45.0
Other relatives	45.1	42.7	44.4
Sister in law/sister	27.1	23.9	26.1
Brother	4.7	7.6	5.6
Neighbour	2.7	7.4	4.1
Other	2.4	3.8	2.9
**Percentage of beneficiaries who came to this facility directly from home for delivery**			
Came directly from home to this facility	92.9	100.0	95.1
Referred from another health facility	7.1	0.0	4.9

Table A3.7 Obstetric history of beneficiary

Particulars	L-3	L-2	Total
**Total number of beneficiaries (unweighted)**	230	94	392
**Total number of beneficiaries (weighted)**	113846	50259	164105
**Average number beneficiary had**			
Pregnancies	2.1	2.3	2.2
Current living children	1.9	2.2	2.0
**Sex of the newborn child**			
Male	50.2	55.1	51.7
Female	49.8	44.9	48.3

Table A3.8 Services received by the beneficiary

Particulars	L-3	L-2	Total
**Total number of beneficiaries (unweighted)**	230	94	392
**Total number of beneficiaries (weighted)**	113846	50259	164105
**% beneficiaries opined maintenance of cleanliness/hygiene in**			
Labour room	98.7	100	99.1
Ward	96.1	96.7	96.3

**Facility of clean toilets in**			
Labour room<br>Did not use<br>No toilet	84.6<br>1.0<br>4.1	82.9<br>4.6<br>0	84.1<br>2.1<br>2.8
Ward<br>Did not use	88.0<br>1.1	83.0<br>0.0	86.5<br>0.7
**Toilets had 24 hour running water**	96.8	85.1	93.2
Not used	0.3	0.0	0.2
Don't know	0.7	0.0	0.5
**Drinking water available throughout the stay in hospital**	81.5	81.3	81.4
**Provsion of clean bed sheets in ward**	70.0	75.6	71.7
**Bedsheets changed daily**	39.0	42.0	39.9
**Free diet services received from facilities**			
Provided and utilized	70.0	75.5	71.7
Provided but not utilized	10.9	7.3	9.8
Not provided	19.1	17.2	18.5
**Satisfied with the quality of food provided in the facility**			
Very satisfied	17.4	16.7	17.2
Satisfied	79.9	83.3	81.0
Dissatisfied	2.6	0.0	1.8
% of beneficiaries who did not incur cost for the delivery in the facility	85.7	82.1	84.6
**Avergae amount spend in the facility for delivery by those who incurred expenses**	390.6	441.9	409.1

Table A3.9 Experience of care received by beneficiaries

Particulars	L-3	L-2	Total
**Total number of beneficiaries (unweighted)**	230	94	392
**Total number of beneficiaries (weighted)**	113846	50259	164105
**% of beneficiaries mentioned that**			
Haemoglobin estimation/blood was taken from finger at admission	58.6	39.4	52.7
BP was checked at the time of admission in labour room	86.0	81.6	84.6
Staff Nurse/ANM put her hand over abdomen at regular intervals during labour	98.6	98.2	98.5
Stethoscope put over abdomen at regular intervals during labour	90.3	81.0	87.5
Doctor examined during labour	66.1	46.8	60.2
Was comfortable during the examination	86.3	78.1	83.8
Privacy maintained during the examination or labour so that people other than facility staff could not see beneficiary	97.5	95.1	96.8
**Baby put on beneficiaries abdomen immediately after delivery**	99.2	100	99.5
**Had problems/ complications during delivery**	14.7	15.3	14.9
**After delivery baby was kept with**			
Mother	90.5	97.1	92.5
Elsewhere	8.8	2.9	7.0
**Child taken for any examination**	73.1	68.3	71.6
Providing of stretcher while shifting from labour room to ward	44.1	22.9	37.6
**% of beneficiary who**			
Initiated breastfeeding within the first hour of birth	85.6	85.3	85.6
Were provided with support for initiating breastfeeding to child	75.5	94.5	81.3
Child is drinking mother's milk	92.0	98.0	93.9
**Check-up done during postnatal visit**<br>**For Mothers**			
Blood pressure	56.7	62.8	58.8
Pulse	25.1	25.0	25.1
Vaginal bleeding	44.5	41.5	43.5

Temperature	26.4	38.4	30.5
Any other	10.1	5.1	8.4
Did not check anything	18.9	18.8	18.9
**For newborn**			
Umbilical stump	8.8	7.7	8.4
Passing stools	22.1	25.3	23.2
Breast feeding	56.1	67.0	59.8
Temperature	33.2	45.4	37.4
General condition	53.1	51.1	52.4
Any other	3.6	6.6	4.6
Did not check anything	8.7	4.6	7.3
**Information provided regarding postnatal visit**			
Continue exclusive breastfeeding on demand	36.8	50.2	41.4
Do not apply anything on the cord stump	15.7	18.3	16.6
Hygiene and hand washing	40.4	49.6	43.6
Drink fluids regularly	15.2	22.0	17.5
Eat regular meals	24.1	21.8	23.3
Post-partum family planning	47.1	59.7	51.4
Other	3.5	1.1	2.7
Did not provide any information	25.2	11.6	20.6
**Beneficiary was able to ask questions about herself/ infant's health**	46.0	54.5	48.6

Respectful maternity care

Table A3.10 General observations

Particulars	L3	L2	Total
**Total number of beneficiaries observed (un-weighted)**	303	96	399
**Total number of beneficiaries observed (weighted)**	115961	51152	167113
Privacy maintained (partition between labour tables)	80.6	91.6	84.0
Service provider was gentle, comforting and reassuring the woman	97.8	99.1	98.2
Friendly/soft tone of the service provider in providing support/ encouragement	97.5	99.1	98.0
Stretcher was provided for shifting from labour room to ward	53.3	32.0	46.8
Any companion of woman's choice was inside the labour room accompanying her	71.7	92.0	77.9
Case sheet filled up to the relevant stage	95.5	87.7	93.1
Labour room register filled up to the relevant stage	96.1	87.7	93.5

Table A3.11 Respect provided during labour room delivery

Particulars	L-3	L-2	Total
**Total number of beneficiaries (unweighted)**	230	94	392
**Total number of beneficiaries (weighted)**	113846	50259	164105
Staffs attended to them when they arrived in facility	99.7	100	99.8
**% beneficiaries mentioned respect/dignity maintained during**			
Vaginal examination	98.9	99.1	99.0
Breast examination	47.9	60.4	51.8
No checking done	32.1	19.8	28.3
Abdominal examination	98.2	99.1	98.5
**Family member/ friend accompanied in labour room at the time of delivery**	64.9	91.4	73.0
**Service provider supported emotionally and encouraged during the process of delivery**	98.0	95.2	97.1

**Beneficiary experienced the following from any nurse/staff of the facility during labour /delivery**<br>Scolded or shouted	5.3	1.8	4.2
Abused	2.1	0.9	1.7
Slapped	0.7	0.9	0.8
Humiliated in any other way	1.3	1.8	1.5
**% perceive service providers were respectful to them**	86.8	87.6	87.1
**Behaviour of the service provider towards beneficiary**			
Behaviour of service provider was very good / right/ talked nicely /okay	90.7	89.1	90.2
Service was good /good care provided	5.2	2.0	4.2
Behaviour not good / scolded	2.4	2.7	2.5

Table A3.12 Satisfaction regarding Respectful Maternity Care

Particulars	L-3	L-2	Total
**Total number of beneficiaries (unweighted)**	230	94	392
**Total number of beneficiaries (weighted)**	113846	50259	164105
**Beneficiary complaint(s)/concerns expressed satisfactorily during her stay**			
Yes, answered satisfactorily	21.5	20.8	21.3
Answered but not satisfied	2.8	4.4	3.3
No complains/concerns	75.6	74.8	75.4
**% satisfied with the way they were attended by the staff while receiving the services**			
Very satisfied	29.8	32.6	30.7
Satisfied	67.5	66.3	67.1
Cannot say	2.7	0.0	1.9
Dissatisfied	0.0	1.1	.3
**% satisfied with the care and treatment at the health facility**			
Very satisfied	29.7	39.1	32.6
Satisfied	69.0	59.8	66.2
Cannot say	1.2	1.1	1.2
**% would like to return to this facility in future to seek any health services**	99.5	100	99.7
**% would recommend this facility to family and friends**	99.5	100	99.7
