# Supplementary material for: Care Around Birth Approach: A Training, Mentoring, and Quality Improvement Model to Optimize Intrapartum and Immediate Postpartum Quality of Care in India
Source: Glob Health Sci Pract. 2021 Sep 30;9(3):590–610. doi: 10.9745/GHSP-D-20-00368 (PMC8514027; doi:10.9745/GHSP-D-20-00368)
Supplement: 20-00368-Sarin-Supplement1.pdf [file 20-00368-Sarin-Supplement1.pdf]

Supplement to: Taneja G, Sarin E, Bajpayee D, et al. Care around birth approach: a training, mentoring, and quality improvement model to optimize intrapartum and immediate postpartum quality of care. Glob Health Sci Pract. 2021;9(3).
https://doi.org/10.9745/GHSP-D-20-00368

USAID – VRIDDHI (Scaling up RMNCH+A Interventions) Project

"CARE AROUND BIRTH" APPROACH

BASELINE ASSESSMENT TOOLS

Supplement to: Taneja G, Sarin E, Bajpayee D, et al. Care around birth approach: a training, mentoring, and quality improvement model to optimize intrapartum and immediate postpartum quality of care. Glob Health Sci Pract. 2021;9(3).
https://doi.org/10.9745/GHSP-D-20-00638

CONTENTS

SECTION 1: BASELINE ASSESSMENT 1: LABOR ROOM ASSESSMENT 2

SECTION 2: BASELINE ASSESSMENT 2: COMPETANCY ASSESSMENTS 26

SECTION 2.1: COMPETENCY ASSESSMENT – MATERNAL HEALTH 27

SECTION 2.2: COMPETENCY ASSESSMENT – NEWBORN HEALTH 35

SECTION 3: BASELINE ASSESSMENT 3: RECORD REVIEW 42

SECTION 1: BASELINE ASSESSMENT 1: LABOR ROOM ASSESSMENT

Supplement to: Taneja G, Sarin E, Bajpayee D, et al. Care around birth approach: a training, mentoring, and quality improvement model to optimize intrapartum and immediate postpartum quality of care. Glob Health Sci Pract. 2021;9(3).
https://doi.org/10.9745/GHSP-D-20-00638

CARE AROUND BIRTH

LABOR ROOM ASSESSMENT

IDENTIFICATION

1.Name of State____

2.Name of District____

3.Name of Block____

4.Name of City/Town/Village in which facility is located___________________

5.Location of the Health facility (Rural 1; Urban 2)…………………………….

6.Type of Facility

HSC/ Sub Centre……………………………………..1
Non 24*7 PHC ……………………………………..2
24*7 PHC……………………………………………...3
Non FRU CHC………………………………………..4
FRU CHC ……………………………………………..5
Sub Divisional Hospital…………………………..6
District Hospital……………………………………7
Medical College Hospital……………………….8
Others(specify)______________________9

7.Level of MCH facility (L1…1; L2 …2; L3… 3)…………………………………….

8.Whether the facility is functioning as Delivery Point as per GOI benchmarks? (Yes..1; No…2)

9.Contact details of key functionaries

Name of the health facility in-charge:_______________________________

Mobile number of health facility in-charge:___________________________

Name of sister/staff in-charge of the labour room/labour room in-charge:_____________

10.Designation of the respondent(s)

	Yes	No
10a.Medical Officer……………………………………	1	2
10b.Staff in-charge of labour room………………	1	2
10c.Pharmacist………………………………………….	1	2
10.d Staff nurse/ANM…………………………………	1	2
10e.Others(specify)______________________	1	2

Supplement to: Taneja G, Sarin E, Bajpayee D, et al. Care around birth approach: a training, mentoring, and quality improvement model to optimize intrapartum and immediate postpartum quality of care. Glob Health Sci Pract. 2021;9(3). https://doi.org/10.9745/GHSP-D-20-00638

SECTION 1: HEALTH FACILITY PROFILE

Q.NO	QUESTION AND FILTER	CODING CATEGORIES	SKIP TO
A.1	Total population covered by the Health facility as per the facility records (for latest year)	Tot. Pop ☐☐☐☐☐☐	
A.2	Total number of in-patient beds in the facility<br>**IF NOT SANCTIONED WRITE "000" IN "SANCTIONED" & "999" IN "AVAILABLE".**<br>**IF BEDS ARE SANCTIONED BUT NOT AVAILABLE THEN WRITE NUMBER OF BEDS SANCTIONED IN "SANCTIONED" & WRITE "000" IN "AVAILABLE"**	Sanctioned……………. ☐☐☐<br>Available………………… ☐☐☐	
A.3	Total number of labour rooms available	No. of Labour room(s) available ☐	
A.4	Total number of labour tables available in the labour room(s).<br>**IF NOT AVAILABLE THEN WRITE "0" IN "AVAILABLE"**	Available…………………….. ☐	
A.5	Total number of major OTs available in the facility.<br>**IF NOT APPLICABLE FOR THE FACILITY CODE "9" IN BOTH "AVAILABLE" & "FUNCTIONAL".**	Available…………………….. ☐<br>Functional…………………… ☐	
A.6	Does this facility have a New Born Stabilisation Unit (NBSU)?	Yes………………………… 1<br>No………………………….. 2<br>Not Applicable………… 9	2, 9 → **A.8**
A.7	Is the NBSU functional?	Yes………………………… 1<br>No………………………….. 2	
A.8	Does this facility have a Special New born Care Unit (SNCU)?	Yes………………………… 1<br>No………………………….. 2<br>Not Applicable………… 9	2, 9 → **A.10**
A.9	Is the SNCU functional?	Yes………………………… 1<br>No………………………….. 2	

Supplement to: Taneja G, Sarin E, Bajpayee D, et al. Care around birth approach: a training, mentoring, and quality improvement model to optimize intrapartum and immediate postpartum quality of care. Glob Health Sci Pract. 2021;9(3).
https://doi.org/10.9745/GHSP-D-20-00638

Q.NO	QUESTION AND FILTER	CODING CATEGORIES	SKIP TO
A.10	Does this facility have Blood Bank?	Yes.......................... 1<br>No.......................... 2<br>Not Applicable............ 9	 2, 9 → A.12
A.11	Is the Blood Bank functional?	Yes.......................... 1<br>No.......................... 2	
A.12	Does this facility have a Blood Storage Unit?	Yes.......................... 1<br>No.......................... 2<br>Not Applicable............ 9	 2, 9 → A.14
A.13	Is the Blood storage unit functional?	Yes.......................... 1<br>No.......................... 2	
A.14	Does this facility have a CSSD?	Yes.......................... 1<br>No.......................... 2<br>Not Applicable............ 9	 2, 9 → A.16
A.15	Is the CSSD functional?	Yes.......................... 1<br>No.......................... 2	
A.16	Does this facility have an in house Laboratory?	Yes.......................... 1<br>No.......................... 2	

Q.NO	QUESTION AND FILTER	**Inhouse 24x7**	**In house not 24x7**	**Outsourced**	**Not available**	SKIP TO
A.17	Do you have provision to conduct the following tests in your health facility at all hours (24x7) or outsourced these service to a private lab where a client can avail the services free of charge at all hours?					
A.17.1	Test for Haemoglobin	1	2	3	4	
A.17.2	Urine test for albumin and sugar	1	2	3	4	
A.17.3	Urine test for pregnancy	1	2	3	4	
A.17.4	Rapid Diagnostic Kit for Malaria	1	2	3	4	
A.17.5	Screening for HIV	1	2	3	4	

Q.NO	QUESTION AND FILTER	CODING CATEGORIES				SKIP TO
**CHECK COVER PAGE "Level of MCH facility" AND MARK "X: IN APPROPRIATE BOX**<br>**L2 &L3** ☐ → **GO TO A.17.6** **L1** ☐ → **GO TO A. 18**						
		**Inhouse 24x7**	**In house not 24x7**	**Outsourced**	**Not available**	
A.17.6	Blood Group	1	2	3	4	
A.17.7	MP slide test…………………………..	1	2	3	4	
A.17.8	Confirmatory test for HIV………………	1	2	3	4	
A.17.9	Blood Glucose…………………………..	1	2	3	4	
A.17.10	CBC……………………………………	1	2	3	4	
A.17.11	Bleeding time and clotting Time………..	1	2	3	4	
A.17.12	Hepatitis B Screening…………………..	1	2	3	4	
A.17.13	Screening for Syphilis-RPR……………..	1	2	3	4	
A.17.14	Liver function test……………………….	1	2	3	4	
A.17.15	USG……………………………………	1	2	3	4	
**SUPPORT/ANCILLARY SERVICES AT FACILITY**						
A.18	Whether the facility has an enquiry/Help desk?<br>**OBSERVE AND RECORD THE ANSWER**	Yes……………………. 1<br>No…………………….. 2<br>Not Applicable………... 9				
A.19	Whether the facility has Security services at all hours?<br>**OBSERVE/ASK AND RECORD THE ANSWER**	Yes……………………. 1<br>No…………………….. 2<br>Not Applicable………... 9				
A.20	Whether this facility has dietary facility/service for indoor patients?	Yes……………………. 1<br>No…………………….. 2<br>Not Applicable………... 9				
A.21	Whether this facility has Laundry services?	Yes……………………. 1<br>No…………………….. 2<br>Not Applicable………... 9				
A.22	Whether the Citizen charter is displayed at prominent place in this facility?<br>**OBSERVE AND RECORD THE ANSWER**	Yes……………………. 1<br>No…………………….. 2<br>Not Applicable………... 9				
A.23	Whether this facility has a functional ambulance service?	Yes……………………. 1<br>No…………………….. 2<br>Not Applicable………... 9				

Supplement to: Taneja G, Sarin E, Bajpayee D, et al. Care around birth approach: a training, mentoring, and quality improvement model to optimize intrapartum and immediate postpartum quality of care. Glob Health Sci Pract. 2021;9(3). https://doi.org/10.9745/GHSP-D-20-00638

SECTION 2: PERFORMANCE PARAMETERS

Data reference period: Three calendar months preceding the date of visit to be collected

(Methodology: Review Facility records/registers / HMIS)

Q.NO	QUESTION AND FILTER	CODING CATEGORIES			SKIP TO
Number of deliveries conducted in the health facility during the last three calendar months<br>**NOTE DOWN NUMBERS FOR PRECEDING THREE REPORTING MONTHS FROM CONCERNED FACILITY RECORDS/ REGISTERS.**					
		**Last Month** ________________ **(month)**	**Last but one month** ________________ **(month)**	**Last but two months** ______________ **(month)**	
C1	Total number of Deliveries	☐☐☐☐	☐☐☐☐	☐☐☐☐	
C1.1	Number of vaginal deliveries	☐☐☐	☐☐☐	☐☐☐	
C1.2	Number of assisted vaginal deliveries	☐☐☐	☐☐☐	☐☐☐	
C1.3	Number of C-sections<br>**IF NOT APPLICABLE RECORD "999".**	☐☐☐	☐☐☐	☐☐☐	
C.2	Total Number of pregnant women who came for delivery to the facility during the last three calendar months referred to higher level health facility<br>**NOTE DOWN NUMBERS FOR PRECEDING THREE REPORTING MONTHS FROM CONCERNED FACILITY RECORDS/ REGISTERS.**<br>**IF NONE RECORD "00".**<br>**RECORD "98" IF REFERRAL REGISTER IS NOT AVAILABLE OR DATA NOT MAINATAINED IN ANY RECORDS & WRITE A NOTE AT THE BOTTOM OF THE PAGE**	**Last Month**<br>☐☐	**Last but one month**<br>☐☐	**Last but two months**<br>☐☐	
C.3	Total Number of Maternal Deaths occurred during the last three calendar months at the facility<br>**IF NONE RECORD "0"**	☐	☐	☐	
C.4	Number of live births out of the total deliveries conducted at the facility during the last three calendar months	☐☐☐	☐☐☐	☐☐☐	
C.4a	Total number of new borns weighed immediately after birth	☐☐☐	☐☐☐	☐☐☐	

Q.NO	QUESTION AND FILTER	CODING CATEGORIES			SKIP TO
		Last Month	Last but one month	Last but two months	
C.5	Number of Low Birth Weight Babies out of the total deliveries conducted during the last three calendar months at the facility **IF NONE RECORD "00"**	☐☐	☐☐	☐☐	
C.6	Number of new born who were referred to another health facility with appropriate infrastructure and staff for better care during the last three calendar months **IF NONE RECORD "00"**	☐☐	☐☐	☐☐	
C.7	Number of new born admitted to NBSU/SNCU during the last three calendar months **IF NONE RECORD "00"** **IF NOT APPLICABLE RECORD "99".**	☐☐	☐☐	☐☐	
C.8	Number of Neonatal deaths out of the total deliveries conducted during the last three calendar months in the facility **IF NONE RECORD "00"**	☐☐	☐☐	☐☐	
C.9	Number of Still births out of the total deliveries conducted during the last three calendar months in the facility **IF NONE RECORD "00"**	☐☐	☐☐	☐☐	
C.10	Number of new born out of the total deliveries conducted at the facility who were given all the birth doses of BCG, OPV and Hepatitis B before discharge **IF NONE RECORD "000"**	☐☐☐	☐☐☐	☐☐☐	
C.10.1	Number of new born given BCG before discharge **IF NONE RECORD "000"**	☐☐☐	☐☐☐	☐☐☐	
C.10.2	Number of new born given Zero dose of OPV before discharge **IF NONE RECORD "000"**	☐☐☐	☐☐☐	☐☐☐	
C.10.3	Number of new born given birth dose of Hepatitis-B before discharge **IF NONE RECORD "000"**	☐☐☐	☐☐☐	☐☐☐	
C.11	Number of Post-Partum Sterilisations conducted during the last three calendar months in the facility **IF NONE RECORD "000"** **IF NOT APPLICABLE RECORD "999".**	☐☐☐	☐☐☐	☐☐☐	
C.12	Number of women who availed PPIUCD services out of the total number of deliveries conducted at the facility **IF NONE RECORD "000"** **IF NOT APPLICABLE RECORD "999".**	☐☐☐	☐☐☐	☐☐☐	

Supplement to: Taneja G, Sarin E, Bajpayee D, et al. Care around birth approach: a training, mentoring, and quality improvement model to optimize intrapartum and immediate postpartum quality of care. Glob Health Sci Pract. 2021;9(3). https://doi.org/10.9745/GHSP-D-20-00638

SECTION 3: HUMAN RESOURCES

Training related data: consider trainings received during the last 5 years

(Methodology: Document review, provider interaction)

Q.NO	QUESTION AND FILTER	CODING CATEGORIES	SKIP TO
D.1.1	Number of Gyn.& Obs. Specialists at the facility<br>**IF NOT SANCTIONED: RECORD "00" IN "No. sanctioned" & "99" IN "No. in position"**<br>**IF SANCTIONED & CURRENTLY POST IS VACANT THEN RECORD "00" IN "No. in position"**	No. sanctioned…………. ☐☐<br>No. in position…………. ☐☐	
D.1.2	*Number of Gyn* .& Obs. Specialists *trained on **lap sterilisation*** **IF NONE RECORD "00"**	No. trained…………….. ☐☐	
D.1.3	*Number of* Gyn.& Obs. Specialists *trained on **PPIUCD*** **IF NONE RECORD "00"**	No. trained…………….. ☐☐	
D.1.4	Is the Gyn & Obs. specialist available at all hours?	Yes……………………… 1<br>No………………………. 2<br>Not Applicable………… 9	
D.2.1	Number of Paediatrics Specialists at the facility<br>**IF NOT SANCTIONED: RECORD "00" IN "No. sanctioned" & "99" IN "No. in position"**<br>**IF SANCTIONED & CURRENTLY POST IS VACANT THEN RECORD "00" IN "No. in position"**	No. sanctioned…………. ☐☐<br>No. in position…………. ☐☐	
D.2.2	Number of Paediatricians trained on **NSSK**<br>**IF NONE RECORD "00"**	No. trained…………….. ☐☐	
D.2.3	Number of Paediatricians trained on **FIMNCI**<br>**IF NONE RECORD "00"**	No. trained…………….. ☐☐	
D.2.4	Number of Paediatricians trained on **FBNC**<br>**IF NONE RECORD "00"**	No. trained…………….. ☐☐	
D.2.5	Is paediatrician available at all hours?	Yes……………………… 1<br>No………………………. 2<br>Not Applicable………… 9	
D.3.1	Number of Anaesthesia Specialist<br>**IF NOT SANCTIONED: RECORD "00" IN "No. sanctioned" & "99" IN "No. in position"**<br>**IF SANCTIONED & CURRENTLY POST IS VACANT THEN RECORD "00" IN "No. in position"**	No. sanctioned…………. ☐☐<br>No. in position…………. ☐☐	
D.3.2	Is an Anaesthesia Specialist available at all hours?	Yes……………………… 1<br>No………………………. 2<br>Not Applicable………… 9	

Q.NO	QUESTION AND FILTER	CODING CATEGORIES	SKIP TO
D.4	Number of General Surgeons at the facility<br>**IF NOT SANCTIONED: RECORD "00" IN "No. sanctioned" & "99" IN "No. in position"**<br>**IF SANCTIONED & CURRENTLY POST IS VACANT THEN RECORD "00" IN "No. in position"**	No. sanctioned…………. ☐☐<br>No. in position…………. ☐☐	
D.5.1	Number of Medical officers at the facility<br>**IF NOT SANCTIONED: RECORD "00" IN "No. sanctioned" & "99" IN "No. in position"**<br>**IF SANCTIONED & CURRENTLY POST IS VACANT THEN RECORD "00" IN "No. in position"**	No. sanctioned…………. ☐☐<br>No. in position…………. ☐☐	
D.5.2	Number of Medical officers trained on **BEmOC**<br>**IF NONE RECORD "00"**	No. trained…………….. ☐☐	
D.5.3	Number of Medical officers trained on **CEmOC**<br>**IF NONE RECORD "00"**	No. trained…………….. ☐☐	
D.5.4	Number of Medical officers trained on **LSAS**<br>**IF NONE RECORD "00"**	No. trained…………….. ☐☐	
D.5.5	Number of Medical officer trained on **PPIUCD**<br>**IF NONE RECORD "00"**	No. trained…………….. ☐☐	
D.5.6	Number of Medical officers trained on **MTP**<br>**IF NONE RECORD "00"**	No. trained…………….. ☐☐	
D.5.7	Number of Medical officers trained on **FBNC**<br>**IF NONE RECORD "00"**	No. trained…………….. ☐☐	
D.5.8	Number of Medical officers trained on **FIMNCI**<br>**IF NONE RECORD "00"**	No. trained…………….. ☐☐	
D.5.9	Number of Medical officers trained on **NSSK**<br>**IF NONE RECORD "00"**	No. trained…………….. ☐☐	
D.5.10	Is there a full time MO available for the LR?	Yes……………………. 1<br>No……………………. 2<br>Not Applicable………… 9	
D.6.1	No. of AYUSH Medical Officers at the facility<br>**IF NOT SANCTIONED: RECORD "00" IN "No. sanctioned" & "99" IN "No. in position"**<br>**IF SANCTIONED & CURRENTLY POST IS VACANT THEN RECORD "00" IN "No. in position"**	No. sanctioned…………. ☐☐<br>No. in position…………. ☐☐	
D.6.2	Number. of AYUSH Medical Officers trained in **SBA**<br>**IF NONE RECORD "00"**	No. trained…………….. ☐☐	
D.7.1	Number of GNM (Nursing Staff) posted currently at LR<br>**IF NOT SANCTIONED: RECORD "00" IN "No. sanctioned" & "99" IN "No. in position"**<br>**IF SANCTIONED & CURRENTLY POST IS VACANT THEN RECORD "00" IN "No. in position"**	No. sanctioned…………. ☐☐<br>No. in position…………. ☐☐	

Supplement to: Taneja G, Sarin E, Bajpayee D, et al. Care around birth approach: a training, mentoring, and quality improvement model to optimize intrapartum and immediate postpartum quality of care. Glob Health Sci Pract. 2021;9(3). https://doi.org/10.9745/GHSP-D-20-00638

Q.NO	QUESTION AND FILTER	CODING CATEGORIES	SKIP TO
D.7.2	Number of GNM (Nursing staff) trained on **SBA**<br>**IF NONE RECORD "00"**	No. trained………………	
D.7.3	Number of GNM (Nursing staff) trained on **NSSK**<br>**IF NONE RECORD "00"**	No. trained………………	
D.7.4	Number of GNM (Nursing staff) trained on **PPIUCD**<br>**IF NONE RECORD "00"**	No. trained………………	
D.7.5	Number of GNM (Nursing staff) trained on **FIMNCI**<br>**IF NONE RECORD "00"**	No. trained………………	
D.7.6	Number of GNM (Nursing staff) trained on **PPTCT**<br>**IF NONE RECORD "00"**	No. trained………………	
D.7.7	Number of GNM (Nursing staff) trained on **FBNC**<br>**IF NONE RECORD "00"**	No. trained………………	
D.8.1	Number of ANM Nursing Staff posted at Labour Room	No. Posted………………	
D.8.2	Number of ANM Nursing Staff trained on **SBA**<br>**IF NONE RECORD "00"**	No. trained………………	
D.8.3	Number of ANM Nursing Staff trained on **NSSK**<br>**IF NONE RECORD "00"**	No. trained………………	
D.8.4	Number of ANM Nursing Staff trained on **PPIUCD**<br>**IF NONE RECORD "00"**	No. trained………………	
D.8.5	Number of ANM Nursing Staff trained on **PPTCT**<br>**IF NONE RECORD "00"**	No. trained………………	
D.9.1	Is a Radiologist / Ultrasonologist available at the facility	Yes…………………….. 1<br>No……………………... 2<br>Not Applicable………… 9	
D.9.2	Is Radiologist / Ultrasonologist available on call during off working hours?	Yes…………………….. 1<br>No……………………... 2<br>Not Applicable………… 9	1 → **D.10.1**<br>9 → **D.10.1**
D.9.3	If no, then is the Obstetrician / MO trained to perform USG ?	Yes…………………….. 1<br>No……………………... 2	
D.10.1	Number of Lab Technicians available at facility<br>**IF NOT SANCTIONED: RECORD "00" IN "No. sanctioned" & "99" IN "No. in position"**<br>**IF SANCTIONED & CURRENTLY POST IS VACANT THEN RECORD "00" IN "No. in position"**	No. sanctioned………….<br>No. in position………….	

Q.NO	QUESTION AND FILTER	CODING CATEGORIES		SKIP TO
D.10.2	Is Lab Technician available on call during off working hours?	Yes……………………..	1	
		No……………………..	2	
		Not Applicable…………	9	

SECTION 4: INFRASTRUCTURE & LAYOUT OF DELIVERY UNIT / LABOUR ROOM

(Methodology – Direct Observation)

Q.NO	QUESTION AND FILTER	CODING CATEGORIES				SKIP TO
	**Layout of Labour Room Unit**		**Yes**	**No**	**NA**	
E.1	Whether LR unit has waiting area for attendants/ASHA?	Waiting area………	1	2		
E.2	Is there a dedicated Receiving area in the delivery unit?	Receiving area……	1	2	9	
E.3	Is there an Examination Room in LR unit?	Examination room..	1	2	9	
E.4	Is there a dedicated Pre delivery room?	Pre delivery room...	1	2	9	
E.5	Is there a dedicated Delivery room?	Dedicated del. room	1	2		
E.6	Is there a Post delivery observation room?	Observation room	1	2	9	
E.7	Is there a dedicated nursing station within or in proximity to labour room?	Nursing station	1	2		
E.8	Is there a New born care Corner within Labour Room?	NBCC in LR	1	2		
E.9	Is there a dedicated Eclampsia room?	Eclampsia room	1	2	9	
E.10	Is there an earmarked room for Sterilisation / autoclaving?	Room: sterlisn./auto.	1	2	9	
E.11	Is there a store room?	Store room	1	2		
	**Layout of Labour Room Unit**					
E.12	Are the corridors connecting the labour room broad enough to manage stretcher and trolleys?	Corridors are broad	1	2		
E.13	Is the Labour room in Proximity and has functional linkage with OT?	linkage with OT	1	2	9	
E.14	Is the Labour room in proximity and has functional linkage with NBSU/SNCU?	linkage with NBSU/SNCU	1	2	9	
E.15	Whether the fire exit in LR is adequate to permit safe escape of its occupants at the time of fire?	Adequate fire exit	1	2		
	**Amenities available in LR**					
E.16	Is there an attached functional toilet facility in LR?	Attached toilet	1	2		
E.17	Whether LR has 24*7 running water facility?	Running water facility	1	2		
E.18	Whether LR has Drinking water supply?	Drinking water	1	2		

Q.NO	QUESTION AND FILTER		CODING CATEGORIES			SKIP TO
	**Amenities available in LR..Contd..**		**Yes**	**No**	**NA**	
E.19	Whether LR has functional telephone and Intercom?	telephone and Intercom	1	2		
E.20	Whether LR has public announcement (PA) system/communication in waiting area?	PA System	1	2	9	
E.21	Does LR have 24 *7 Electricity supply (functional Power Backup)	24*7 Electricity	1	2		
E.22	Is the LR air-conditioned (Functional AC)	Air conditioning	1	2		
E.23	Is there cross ventilation available in the LR with an exhaust fan?	Cross ventilation	1	2	9	
E.24	Is there a functional Room Heater?	Room heater	1	2	9	
E.25	Is there a functional Refrigerator?	Refrigerator	1	2		
	**Safety/Privacy measures in LR**					
E.26	Are the floors of the labour room non-skid and even?		1	2		
E.27	Are the walls tiled up to 6 ft?		1	2		
E.28	Do the windows have grills and wire meshwork?		1	2	9	
E.29	Is privacy maintained at the windows (eg. Provision of Frosted Glass)?		1	2	9	
E.30	Is a functional fire Extinguisher installed in the Labour Room?		1	2		
E.31	Are fire exits clearly visible and routes to reach exit are clearly marked?		1	2		
	**General Equipment / Furnishing**					
E.32	Is a complaint/suggestion box available in waiting area		1	2	9	
E.33	Are Stepping stools available with each Labour table?		1	2		
E.34	Are Wheel chairs/patient's trolleys available for use in LR?		1	2		
E.35	Are Mosquito Repellents available?		1	2		
	**Signage system**					
E.36	Is there a clear signage at entrance of the facility (near registration counter) to direct patients to LR?	Signage to LR at entrance	1	2		
E.37	Is there signage for Restricted Area (RA) displayed outside LR?	Signage for RA	1	2		
E.38	Are Names of Doctors and Nurses on duty in LR displayed outside the LR?	LR staff name displayed	1	2		
E.39	Whether the Services available in LR displayed?	Services in LR displayed	1	2		
E.40	Whether the signages and information displayed are also available in local language?	Signage & info. In local language	1	2		

Supplement to: Taneja G, Sarin E, Bajpayee D, et al. Care around birth approach: a training, mentoring, and quality improvement model to optimize intrapartum and immediate postpartum quality of care. Glob Health Sci Pract. 2021;9(3). https://doi.org/10.9745/GHSP-D-20-00638

Q.NO	QUESTION AND FILTER		CODING CATEGORIES			SKIP TO
	**Display of Protocols in LR (appropriate for the level of facility)- Are the following protocols displayed**					
	**Display of Protocols in LR ..Contd..**		**Yes**	**No**	**NA**	
E41.1	Simplified Partograph	Simplified Partograph	1	2		
E41.2	Vaginal bleeding before 20 weeks	Vaginal bleeding<20wks	1	2	9	
E41.3	Vaginal bleeding after 20 weeks	Vaginal bleeding>20wks	1	2	9	
E41.4	Management of PPH	PPH management	1	2	9	
E41.5	Eclampsia	Eclampsia	1	2	9	
E41.6	AMTSL	AMTSL	1	2	9	
E41.7	New born resuscitation	New born resuscitation	1	2	9	
E41.8	Kangaroo Mother Care	Kangaroo Mother Care	1	2	9	
E41.9	Breastfeeding	Breastfeeding	1	2	9	
E41.10	Hand washing	Hand washing	1	2	9	
E41.11	Preparation of 1 litre bleaching soln.	Prep. Of bleachng. soln.	1	2	9	
E41.12	Infection prevention	Infection prevention	1	2	9	
E41.13	Processing of used items	Processing of used items	1	2	9	
E41.14	LR Sterilization	LR Sterilization	1	2	9	
E41.15	Management of atonic PPH	Atonic PPH	1	2	9	
	**IEC Display in facility in Admission/ OPD area (in Local language)**					
E42.1	Whether JSY entitlements displayed (wall painting/banner etc) in admission/OPD area?	JSY entitlements displayed	1	2		
E42.2	Whether JSSK entitlements displayed (wall painting/banner etc) in admission/OPD area?	JSSK entitlements displayed	1	2		
E42.3	Whether Referral Transport Details displayed (wall painting/banner etc) in admission/OPD area?	Referral Transport Details displayed	1	2	9	

SECTION 5: MEDICAL EQUIPMENT & FURNISHINGS

(Methodology: Direct Observation)

Q.NO	QUESTION AND FILTER		CODING CATEGORIES			SKIP TO
	**Equipment / Furnishing**		**Yes**	**No**	**NA**	
F.1	Is there a mattress on every delivery table?	mattress on delivery table	1	2		
F.2	Is there a Mackintosh with each labour table (LT)?	Mackintosh on LT	1	2		
F.3	Is there a functional Kellys pads on each of LT?	Kellys pads on LT	1	2		
F.4	Whether autoclaved delivery sets readily available?	Autoclaved delivery set	1	2		
F.5	Is there a Modular Light for conducting deliveries?	Modular light	1	2		
F.6	Is there a wall clock with seconds hand / Digital in LR?	Wall clock	1	2		
F.7	Is there a wall mounted thermometer for measuring room temperature?	wall mounted thermometer	1	2		
F.8	Is there a functional Haemoglobinometer with reagents and lancet?	Haemoglobinometer	1	2		
F.9.1	Is there a functional Suction apparatus- Electric in LR?	Suction apparatus	1	2	9	
F.9.2	Is there a functional foot operated Suction apparatus LR?	foot operated Suction apparatus	1	2	9	
F.9.3	Is there a functional Suction apparatus- Central in LR?	Suction apparatus- Central	1	2	9	
F.10	Is there a movable Delivery trolley in LR?	Delivery trolley	1	2		
F.11.1	Is there a functional Oxygen cylinder in LR?	Oxygen cylinder	1	2	9	
F.11.2	Is there a functional Oxygen Concentrator in LR?	Oxygen Concentrator	1	2	9	
F.11.3	Is there a functional Central oxygen supply in LR?	Central oxygen supply	1	2	9	
F.12	Is there a Screen/Partition between two tables for privacy?	Screen/Partition	1	2	9	
F.13	Is there a functional Adult Weighing Scale in LR?	Adult Weighing Scale	1	2		
F.14	Is there a functional Pulse Oximeter(only at L3) in LR?	Pulse Oximeter	1	2	9	
F.15	Is there a functional Foetal Doppler(Only at L3) in LR?	Foetal Doppler	1	2	9	
F.16	BP apparatus		1	2		
F.17	Adult Stethoscope		1	2		
F.18	Fetoscope		1	2		
F.19	Ambu bag		1	2		
F.20	Oxygen hood (Adult)		1	2		
F.21	Autoclave		1	2	9	

Supplement to: Taneja G, Sarin E, Bajpayee D, et al. Care around birth approach: a training, mentoring, and quality improvement model to optimize intrapartum and immediate postpartum quality of care. Glob Health Sci Pract. 2021;9(3).
https://doi.org/10.9745/GHSP-D-20-00638

Q.NO	QUESTION AND FILTER	CODING CATEGORIES		SKIP TO
		Yes	No	
F.22	Pediatric stethoscope	1	2	
F.23	Baby weighing scale	1	2	
F.24	Radiant warmer	1	2	
F24.1	Does the radiant warmer have a dedicated stabiliser?	1	2	
	**New born tray**			
F.25	New born Thermometer	1	2	
F.26	Self-inflating bag for New Born resuscitation	1	2	
F.27	Mask-neonatal size (0)	1	2	
F.28	Mask-neonatal size (1)	1	2	
F.29	Oxygen hood (neonatal)	1	2	
F.30	Dee Lees mucus extractor	1	2	
F.31	Two pre warmed towels/sheets for wrapping the baby	1	2	
F.32	Sterilized thread /cord clamp	1	2	
F.33	Needle (26 gauge) and syringe (1ml)	1	2	
F.34	Inj. Vitamin K 1	1	2	
F.35	Shoulder Roll	1	2	
F.36	Designated New born tray present	Yes………………………………<br>No…………………………………	1<br>2	

SECTION 6: DRUGS & CONSUMABLES AND VACCINES

(Methodology: observation, Record review, Provider interaction in the Labour room & Medical store)

A. **Availability of Labour Room Trays** *(Observation)*

Q.NO	QUESTION AND FILTER	CODING CATEGORIES				SKIP TO
	Check if following items are available in designated Trays and / or individually	**Designated tray**	**Individually**	**Not available**	**NA**	
	**Delivery tray**					
G.1.a	Scissors	1	2	3	9	
G.1.b	Artery forceps	1	2	3	9	
G.1.c	Sponge holding forceps	1	2	3	9	
G.1.d	Speculum	1	2	3	9	
G.1.e	Urinary catheter	1	2	3	9	
G.1.f	BP blade / surgical blade for cutting cord	1	2	3	9	
G.1.g	Bowl for antiseptic solution	1	2	3	9	
G.1.h	kidney tray	1	2	3	9	
G.1.i	Gauze pieces; cotton swabs	1	2	3	9	

Supplement to: Taneja G, Sarin E, Bajpayee D, et al. Care around birth approach: a training, mentoring, and quality improvement model to optimize intrapartum and immediate postpartum quality of care. Glob Health Sci Pract. 2021;9(3). https://doi.org/10.9745/GHSP-D-20-00638

Q.NO	QUESTION AND FILTER	CODING CATEGORIES				SKIP TO
G.1.j	Cord clamps and ligatures	1	2	3	9	
G.1.k	Sanitary pads	1	2	3	9	
G.1.l	Gloves	1	2	3	9	
G.1.m	Designated Delivery tray present	Yes……………………………………..<br>No……………………………………..			1<br>2	
G.1.n	Total number of delivery trays present in LR<br>**IF NONE RECORD "0"**	Number of delivery trays…..				
G.2	**Episiotomy tray**	**Designated tray**	**Individually**	**Not available**	**NA**	
G.2.a	Inj. Xylocaine 2%	1	2	3	9	
G.2.b	10ml disposable syringe with needle	1	2	3	9	
G.2.c	Episiotomy scissor	1	2	3	9	
G.2.d	Artery forceps	1	2	3	9	
G.2.e	Allis forceps	1	2	3	9	
G.2.f	Sponge holding forceps	1	2	3	9	
G.2.g	Toothed forceps	1	2	3	9	
G.2.h	Thumb forceps	1	2	3	9	
G.2.i	Kidney tray	1	2	3	9	
G.2.j	Needle holder	1	2	3	9	
G.2.k	Needle (round body and cutting)	1	2	3	9	
G.2.l	Chromic catgutno.0	1	2	3	9	
G.2.m	Gauze pieces; cotton swabs	1	2	3	9	
G.2.n	Antiseptic solution	1	2	3	9	
G.2.o	Gloves	1	2	3	9	
G.2.p	Designated Episiotomy tray present	Yes……………………………………..<br>No……………………………………..			1<br>2	
G.3	**Medicine tray**	**Designated tray**	**Individually**	**Not available**	**NA**	
G.3.a	Inj. Oxytocin (to be kept in the refrigerator)	1	2	3	9	
G.3.b	Inj Gentamycin	1	2	3	9	
G.3.c	Inj Vit K1	1	2	3	9	
G.3.d	Inj Betamethasone	1	2	3	9	
G.3.e	Inj Hydralazine	1	2	3	9	
G.3.f	Cap Ampicillin	1	2	3	9	
G.3.g	Tab Metronidazole	1	2	3	9	
G.3.h	Tab Paracetamol	1	2	3	9	
G.3.i	Tab Ibuprofen	1	2	3	9	
G.3.j	Tab B Complex	1	2	3	9	

Q.NO	QUESTION AND FILTER	CODING CATEGORIES				SKIP TO
	**Medicine tray …contd.**	**Designated tray**	**Individually**	**Not available**	**NA**	
G.3.k	Tab Misoprostol	1	2	3	9	
G.3.l	Tab Nifedipine	1	2	3	9	
G.3.m	Tab Methyldopa	1	2	3	9	
G.3.n	IV fluids (Ringer Lactate, Normal saline)	1	2	3	9	
G.3.o	Magnifying Glass	1	2	3	9	
G.3.p	Nevirapine and other HIV drugs (Only for ICTC and ART centers)	1	2	3	9	
G.3.q	Designated Medicine tray present	Yes…………………………………<br>No…………………………………			1<br>2	
	**Emergency drug tray**	**Designated tray**	**Individually**	**Not available**	**NA**	
G.4.a	Inj Oxytocin (to be kept in fridge)	1	2	3	9	
G.4.b	Inj Magesium sulphate 50%	1	2	3	9	
G.4.c	Inj calcium gluconate 10%	1	2	3	9	
G.4.d	Inj Dexamethasone	1	2	3	9	
G.4.e	Inj Ampicillin	1	2	3	9	
G.4.f	Inj Gentamycin	1	2	3	9	
G.4.g	Inj Metronidazole	1	2	3	9	
G.4.h	Inj Lignocaine 2%	1	2	3	9	
G.4.i	Inj Adrenaline	1	2	3	9	
G.4.j	Inj Hydrocortisone succinate	1	2	3	9	
G.4.k	Inj Diazepam	1	2	3	9	
G.4.l	Inj Pheniramine maleate	1	2	3	9	
G.4.m	Inj Carboprost	1	2	3	9	
G.4.n	Inj Pentazocin chloride	1	2	3	9	
G.4.o	Inj Promethazine	1	2	3	9	
G.4.p	Inj Betamethasone	1	2	3	9	
G.4.q	Inj Hydralazine	1	2	3	9	
G.4.r	IV fluids - RL, NS	1	2	3	9	
G.4.s	IV sets with at least two 16 guage needles	1	2	3	9	
G.4.t	IV cannula	1	2	3	9	
G.4.u	Vials for blood collection	1	2	3	9	
G.4.v	syringes and needles	1	2	3	9	
G.4.w	Tab nifedipine	1	2	3	9	
G.4.x	Tab methyldopa	1	2	3	9	

Supplement to: Taneja G, Sarin E, Bajpayee D, et al. Care around birth approach: a training, mentoring, and quality improvement model to optimize intrapartum and immediate postpartum quality of care. Glob Health Sci Pract. 2021;9(3).
https://doi.org/10.9745/GHSP-D-20-00638

Q.NO	QUESTION AND FILTER	CODING CATEGORIES				SKIP TO
	**Emergency drug tray..contd**	**Designated tray**	**Individually**	**Not available**	**NA**	
G.4.y	Suction catheter	1	2	3	9	
G.4.z	Mouth gag	1	2	3	9	
G.4.aa	Designated Emergency medicine tray present	Yes...<br>No...			1<br>2	
G.5	**MVA tray**	**Designated tray**	**Individually**	**Not available**	**NA**	
G.5.a	Gloves	1	2	3	9	
G.5.b	Speculum	1	2	3	9	
G.5.c	Anterior vaginal wall retractor	1	2	3	9	
G.5.d	Posterior vaginal wall retractor	1	2	3	9	
G.5.e	Sponge holding forceps	1	2	3	9	
G.5.f	MVA syringe and cannulas	1	2	3	9	
G.5.g	MTP cannulas	1	2	3	9	
G.5.h	Urinary catheter	1	2	3	9	
G.5.i	Small bowl for antiseptic solution	1	2	3	9	
G.5.j	Sterilised gauze/ pads	1	2	3	9	
G.5.k	Cotton swabs	1	2	3	9	
G.5.l	Disposable syringe and needle	1	2	3	9	
G.5.m	Tab Misoprostol	1	2	3	9	
G.5.n	Designated MVA tray present	Yes...<br>No...			1<br>2	
G.6	**PPIUCD tray**	**Designated tray**	**Individually**	**Not available**	**NA**	
G.6.a	PPIUCD insertion forceps	1	2	3	9	
G.6.b	Sims speculum	1	2	3	9	
G.6.c	Sponge holding forceps	1	2	3	9	
G.6.d	Cu IUCD 380A in sterile pack	1	2	3	9	
G.6.e	Cu IUCD 375 in sterile pack	1	2	3	9	
G.6.f	Cotton swabs	1	2	3	9	
G.6.g	Betadine solution	1	2	3	9	
G.6.h	Designated PPIUCD tray present	Yes...<br>No...			1<br>2	

Supplement to: Taneja G, Sarin E, Bajpayee D, et al. Care around birth approach: a training, mentoring, and quality improvement model to optimize intrapartum and immediate postpartum quality of care. Glob Health Sci Pract. 2021;9(3). https://doi.org/10.9745/GHSP-D-20-00638

B. Drug / Commodity

Record the existing stock and if there has been any stock out of the following key Drugs / commodities over the past 3 months at the facility. *(Methodology: Record review in Medical Store)*

Q.NO	QUESTION AND FILTER	CODING CATEGORIES			SKIP TO
**G.7**	**Key Drug / Commodity**	**Stock available at the time of assessment**		**Total number days the drug is out of stock (enter days, if NA write "99" in box)**	
		**Yes**	**No**		
G.7.1	Inj. Oxytocin 10IU	1	2	☐☐	
G.7.2	Tab Misoprostol 200 mcg	1	2	☐☐	
G.7.3	Inj. Magnesium Sulfate 50%	1	2	☐☐	
G.7.4	Inj. Ampicillin 500mg	1	2	☐☐	
G.7.5	Inj. Gentamicin 80 mg	1	2	☐☐	
G.7.6	Inj. Vit. K1	1	2	☐☐	
G.7.7	Inj. Dexamethasone Sodium Phosphate	1	2	☐☐	
G.7.8	IV Fluids (Normal Saline)	1	2	☐☐	
G.7.9	IUCD 380	1	2	☐☐	

SECTION 7: Documentation & Records

Available and Updated for last 3 calendar months

(Methodology: Direct Observation)

Q.NO	QUESTION AND FILTER	CODING CATEGORIES						SKIP TO
	**Records / Documents**	**Available**			**Updated**			
		**Yes**	**No**	**NA**	**Yes**	**No**	**NA**	
H.1	Labour Room Register	1	2	9	1	2	9	
H.2	Partograph in case sheets	1	2	9	1	2	9	
H.3	Referral Register (In)	1	2	9	1	2	9	
H.4	Referral Register (Out)	1	2	9	1	2	9	
H.5	Referral slips	1	2	9	1	2	9	
H.6	Maternal death record Register	1	2	9	1	2	9	
H.7	Discharge Register	1	2	9	1	2	9	
H.8	Discharge slip	1	2	9	1	2	9	

Q.NO	QUESTION AND FILTER	CODING CATEGORIES						SKIP TO
	**Records / Documents…….Contd.**	**Available**			**Updated**			
		**Yes**	**No**	**NA**	**Yes**	**No**	**NA**	
H.9	PPIUCD Register	1	2	9	1	2	9	
H.10	LR sterilization Register	1	2	9	1	2	9	
H.11	Equipment Stock Register	1	2	9	1	2	9	
H.12	Drug Stock Register	1	2	9	1	2	9	
H.13	Handing over-taking over Register	1	2	9	1	2	9	
H.14	PNC Register	1	2	9	1	2	9	
H.15	Admission register	1	2	9	1	2	9	
H.16	MTP register	1	2	9	1	2	9	
H.17	Admission sheets/ BHT	1	2	9	1	2	9	
H.18	FP register	1	2	9	1	2	9	
H.19	Laboratory register	1	2	9	1	2	9	

SECTION 8: INFECTION CONTROL IN LABOUR ROOM

(Methodology: Direct Observation / Staff interaction)

Q.NO	QUESTION AND FILTER	CODING CATEGORIES		SKIP TO
	**Check for availability of the following in the Labour Room**	**Yes**	**No**	
	**Hand hygiene & antisepsis**			
I.1	Availability of hand washing Facility at Point of Use (Sink with running water)	1	2	
I.2	Availability of antiseptic soap with soap dish/ liquid antiseptic with dispenser.	1	2	
I.3	Availability of Alcohol based Hand rub	1	2	
I.4	Availability of elbow operated taps	1	2	
I.5	Availability of Antiseptic Solutions	1	2	
I.6	Hand washing sink is wide and deep enough to prevent splashing and retention of water	Yes………… 1<br>No………….. 2<br>Not applicable……… 9		

Q.NO	QUESTION AND FILTER	CODING CATEGORIES		SKIP TO
	**Material for personal protection**	**Yes**	**No**	
I.7	Availability of Masks	1	2	
I.8	Sterile gloves are available at labour room	1	2	
I.9	Use of elbow length gloves for obstetrical purpose	1	2	
I.10	Availability of gown/ Apron	1	2	
I.11	Availability of shoe cover/gum boots	1	2	
I.12	Availability of Caps	1	2	
I.13	Heavy duty gloves and gum boats for housekeeping staff	1	2	
I.14	Personal protective kit for delivering HIV patients	1	2	
	**Environment control of patient care areas**			
I.15	Facility layout ensures separation of routes for clean and dirty items	1	2	
I.16	Availability of disinfectant as per requirement (Chlorine solution, Gluteraldehye)	1	2	
I.17	Availability of cleaning agent as per requirement (Hospital grade phenyl, disinfectant detergent solution)	1	2	
I.18	Staff is trained for spill management	1	2	
I.19	Cleaning of patient care area with detergent solution	1	2	
I.20	Staff is trained for preparing cleaning solution (0.5% chlorine solution)as per standard procedure	1	2	
I.21	External footwear is restricted	1	2	

Supplement to: Taneja G, Sarin E, Bajpayee D, et al. Care around birth approach: a training, mentoring, and quality improvement model to optimize intrapartum and immediate postpartum quality of care. Glob Health Sci Pract. 2021;9(3). https://doi.org/10.9745/GHSP-D-20-00638

Q.NO	QUESTION AND FILTER	CODING CATEGORIES		SKIP TO
	**BMW management**	**Yes**	**No**	
I.22	Availability of colour coded bins at point of waste generation	1	2	
I.23	Availability of plastic colour coded plastic bags	1	2	
I.24	Segregation of different category of waste as per guidelines	1	2	
I.25	There is no mixing of infectious and general waste	1	2	
I.26	Availability of functional needle cutters	1	2	
I.27	Availability of puncture proof box	1	2	
I.28	Disinfection of sharp before disposal	1	2	
I.29	Transportation of bio medical waste is done in close container/trolley	1	2	
I.30	Is the Method of waste disposal -			
I.30.1	Onsite burying	1	2	
I.30.2	Onsite incineration	1	2	
I.30.3	Agency collection	1	2	
I.31	**If yes for onsite disposal:** Is/ are pit(s) available for Placenta	Yes………………………………. 1<br>No……………………………… 2<br>Not applicable…………………… 9		
	**Instrument processing**	**Yes**	**No**	
I.32	Are the instruments dipped in 0.5% chlorine solution immediately after use (decontamination)?	1	2	
I.33	Are the instruments sterilised using			
I.33.1	an autoclave	1	2	
I.33.2	a boiler	1	2	
I.33.3	Chemical method	1	2	
I.34	Are processed instruments stored in a dry and clean environment to ensure that the instruments remain sterile?<br>(**Remark**: Wrapped and stored in Covered tray)	1	2	

Supplement to: Taneja G, Sarin E, Bajpayee D, et al. Care around birth approach: a training, mentoring, and quality improvement model to optimize intrapartum and immediate postpartum quality of care. Glob Health Sci Pract. 2021;9(3).
https://doi.org/10.9745/GHSP-D-20-00638

SECTION 9. CLIENT FEEDBACK

(Feedback to be collected using the Client feedback tool provided and the inputs collated below)

I. Admission process and quality of services at the facility

Q.NO	QUESTION AND FILTER	Write Responses using codes given below: Yes =1 No =2 Don't Know =8					SKIP TO
		Client 1	Client 2	Client 3	Client 4	Client 5	
J.Q.1	Was all relevant information displayed at the reception/registration counter?						
J.Q.2	Was there any difficulty to get admitted, when you had arrived for delivery?<br>If yes, what difficulty? **WRITE DIFFICULTY FACED AT THE BOTTOM OF THE PAGE**						
J.Q.3	Was there any staff to attend to you, as you arrived?						
J.Q.4	Are you satisfied by the care and treatment provided at the health facility?						
J.Q.5	Were you able to ask questions about yourself and your infant's health?						
J.Q.6	Did you get satisfactory answers to your questions?						
J.Q.7	Are you happy with the way you were attended by the staff while you were provided the services?						
J.Q.8	Would you like to return to this facility to treat or to get advice on any of your future health problems?						
J.Q.9	Would you recommend this facility to family and friends?						
	II. **Cleanliness, Privacy and facilities at the Labour Room / Ward**						
J.Q.10	In your opinion, was cleanliness/hygiene maintained in the patient wards/rooms?						
J.Q.11	Were clean bed sheets provided and changed frequently?						
J.Q.12	Were the toilet(s) in LR/ward clean?						
J.Q.13	Were you satisfied with the privacy that you got in the labour room?						
J.Q.14	Was there availability of water throughout the day?						
J.Q.15	Were you satisfied with the quality of food provided?						

Q.NO	QUESTION AND FILTER	Write Responses using codes given below: Yes =1 No =2 Don't Know =8					SKIP TO
		**Client 1**	**Client 2**	**Client 3**	**Client 4**	**Client 5**	
	**III. Expenses incurred for the Delivery**						
J.Q.16	Did you incur any expenses out of your pocket for the following?						
J.Q.16.1	Transport to reach the facility from your home?						
J.Q.16.2	To Arrange transport to go back from hospital to your home?						
J.Q.16.3	To feed yourself while admitted in the ward						
J.Q.16.4	To buy medicines and consumables like gloves						
J.Q.16.5	To conduct tests						

Assessor: ……………………………………. Signature: ………………………………… Date: ……………………………………

Supplement to: Taneja G, Sarin E, Bajpayee D, et al. Care around birth approach: a training, mentoring, and quality improvement model to optimize intrapartum and immediate postpartum quality of care. Glob Health Sci Pract. 2021;9(3).
https://doi.org/10.9745/GHSP-D-20-00638

SECTION 2: BASELINE ASSESSMENT 2: COMPETANCY ASSESSMENTS

Supplement to: Taneja G, Sarin E, Bajpayee D, et al. Care around birth approach: a training, mentoring, and quality improvement model to optimize intrapartum and immediate postpartum quality of care. Glob Health Sci Pract. 2021;9(3). https://doi.org/10.9745/GHSP-D-20-00638

SECTION 2.1: COMPETENCY ASSESSMENT – MATERNAL HEALTH

Supplement to: Taneja G, Sarin E, Bajpayee D, et al. Care around birth approach: a training, mentoring, and quality improvement model to optimize intrapartum and immediate postpartum quality of care. Glob Health Sci Pract. 2021;9(3). https://doi.org/10.9745/GHSP-D-20-00638

CARE AROUND BIRTH

COMPETENCY ASSESSMENT – MATERNAL HEALTH

State: …………………………. District: ………………………. Block: ……………………. Facility ……………………………..
Name: …………………………………………. Designation: Staff Nurse / ANM Years of service …………………….

Training status (please encircle as yes or no):

Skilled Birth Attendant	Yes / No
Navjat Shishu Suraksha Karyakram	Yes / No
Post-Partum IUCD	Yes / No

Case study: To fill in the Partograph

Rani (wife of Rambhajan), 18 years of age, was admitted to your hospital today at 10:00 am with complaints of 39 weeks pregnancy and labour pains since 7:00 am. This is her first pregnancy.
You are attending to Rani at the facility.

Plot the following findings on the partograph:

At **10:00 am**:

- The cervix is dilated 5 cm.
- She had 2 contractions in 10 minutes, each lasting less than 20 seconds.
- The FHR is 140/minute.
- The membranes are intact.
- Her blood pressure is 100/70 mmHg.
- Her temperature is 37° C.
- Her pulse is 80 per minute.

10:30 am: FHR 140, contractions 2/10 each 20 seconds, pulse 90/minute
11:00 am: FHR 136, contractions 2/10 each 20 seconds, pulse 88/minute
11:30 am: FHR 140, contractions 2/10 each 20 seconds, pulse 84/minute
12:00 noon: FHR 136, contractions 3/10 each 30 seconds, pulse 88/minute, membranes ruptured, amniotic fluid clear
12:30 pm: FHR 146, contractions 3/10 each 35 seconds, pulse 90/minute, amniotic fluid clear
1:00 pm: FHR 150, contractions 4/10 each 40 seconds, pulse 92/minute, amniotic fluid clear
1:30 pm: FHR 160, contractions 4/10 each 45 seconds, pulse 94/minute, amniotic fluid clear
At 2:00 pm:

- Cervix dilated 6 cm
- Amniotic fluid meconium-stained
- Contractions 4/10 each 45 seconds
- FHR 162/minute
- Pulse 100/minute
- Temperature 37.6° C
- Blood pressure 130/80 mmHg

Q 1. What action would you take at 2:00pm? **Enter it on the Partograph**

Knowledge assessment: Active Management of Third Stage of Labour

S. No **Please encircle the correct option in the following multiple choice questions. Please note that each question has only one correct answer.**

1. The third stage of labour is the period from -
   a) Full dilatation of cervix to delivery of baby
   b) Delivery of baby to delivery of placenta
   c) Delivery of placenta to two hours after
   d) Initiation of true labour pain to full dilatation of cervix

2. The following is a critical component of Active management of Third stage of Labour
   a) Administration of Antibiotic
   b) Administration of Uterotonic
   c) Delayed cord cutting
   d) Inspection of the placenta

3. The uterotonic as part of AMTSL ,is ideally administered
   a) Within one minute of birth of baby
   b) Within 1 min of delivery of placenta
   c) Just before delivery of baby
   d) At fetal crowning

4. The drug of choice as a uterotonic for use in AMTSL in a facility is -
   a) Injection Methergine
   b) Injection Oxytocin
   c) Tablet Misoprostol
   d) a & b

5. Under ideal condition, oxytocin should be stored
   a) In a refrigerator
   b) In an open kidney dish in the delivery room
   c) In a drawer in the Nurse’s office, away from light
   d) In the Freezer compartment of the refrigerator

6. If placenta is undelivered after 30 minutes of oxytocin administration and controlled cord traction and the uterus is contracted
   a) More aggressive controlled cord traction should be attempted
   b) Controlled cord traction and fundal pressure should be attempted
   c) The woman to be referred to a facility with emergency obstetric services (FRU)
   d) Stop controlled cord traction and wait for signs of placental separation

7. During third stage of labour, **we must NOT**

a) Administer uterotonic
b) Do controlled cord traction
c) Perform uterine fundal massage
d) Give fundal pressure per abdomen

8. The practice of AMTSL helps in preventing

a) Eclampsia
b) Post-partum hemorrhage
c) Sepsis
d) Obstructed Labour

9. What should you rule out before administering an uterotonic drug?

a) Pulsation of the umbilical cord
b) Uterine contractedness
c) The presence of another baby
d) Signs of placenta separation (e.g., lengthening of the cord)

10. The commonest cause of post-partum hemorrhage is:

a) Retained placenta
b) Genital lacerations
c) Uterine atony
d) Prolonged Labour

Knowledge assessment- Post natal monitoring

S.No	Please encircle the correct option in the following multiple choice questions. Please note that each question has only one correct answer.
1.	The fourth stage of labor is

a) It is the stage from delivery of baby to delivery of placenta
b) It is the stage from delivery of placenta up to two hours
c) It is the stage from delivery of baby up to two hours
d) It is the stage from delivery of placenta up to four hours

2. Savita gave birth to a healthy baby girl one hour ago. You managed the third stage of labor actively, the placenta was complete, and she had no perineal or vaginal lacerations. How often should you monitor her uterus and vaginal bleeding during the second hour after delivery of the placenta?

    a) Every 10 minutes
    b) Every 15 minutes
    c) Continuously
    d) Every 60 minutes

3. The woman should be counselled and supported to breast feed the newborn

    a) As early as possible within 1 hour of birth
    b) Only After the woman has been shifted to the ward
    c) Only after the mother has bathed after delivery
    d) Within 2 days of delivery

4. If the uterus is not firm even after one hour of delivery of placenta, what does it indicate?

    a) Uterus may have placental remnants
    b) Lacerations in genital tract
    c) Uterine atony
    d) a & c
    e) a & b

5. In the postpartum period the mother needs to be counselled on

    a) Exclusive breastfeeding, including colostrum feeding,
    b) To take adequate rest, sleep
    c) Maintain hygiene including perineal hygiene, and washing her hands before handling the baby.
    d) All of the above

6. The birth companion should be asked to stay with the mother and newborn and call for help in the following condition

    a) Mother feeds baby
    b) Mother complains of severe headache
    c) Every time mother passes urine
    d) Only when it is time for discharge

7. Government of India recommends that all woman delivering in a public health facility should stay in the facility for a minimum period of

   a) 24 hours
   b) 48 hours
   c) 72 hours
   d) 12 hours

8. How frequently should a newborn be monitored for breathing and temperature within one hour of birth?

   Response:

   a) Every 10 minutes
   b) Every 15 minutes
   c) Every 20 minutes
   d) Every 30 minutes

9. How many times a healthy newborn should take breastfeeds within 24 hours?

   a) At least 7 times
   b) At least 8 times
   c) At least 9 times
   d) At least 10 times

10. What is the normal range of temperature of a newborn?

   a) 34.5-35.5^{0}C
   b) 35.5-36.5^{0}C
   c) 36.5-37.5^{0}C
   d) 37.5-38.5^{0}C

Knowledge assessment- Infection Prevention

S.No	**Please encircle the correct option in the following multiple choice questions. Please note that each question has only one correct answer.**
1.	Contaminated instruments in the labor ward should immediately be

   a) Soaked in 0.5% chlorine solution for 10 minutes
   b) Soaked in 0.5% chlorine solution for 30 minutes
   c) Washed with soap and water and soaked in 0.5% chlorine solution for 10 minutes
   d) Washed with soap and water and soaked in 0.5% chlorine solution for 30 minutes

2. When taking blood samples or establishing an intravenous line
   a) It is not necessary to wear gloves
   b) Gloves should always be worn
   c) Gloves should be worn only if the patient is HIV+
   d) Gloves should be worn only if the patient is a case of Tuberculosis

3. The placenta is disposed in which of the following bags?
   a) Red bag
   b) Blue bag
   c) Black bag
   d) Yellow bag

4. Which of the following is the most appropriate sequence of processing soiled instruments?
   a) Decontamination→ Cleaning→ Sterilization
   b) Cleaning→ Decontamination→ Sterilization
   c) Sterilization → Cleaning→ Decontamination
   d) Decontamination→ Sterilization → Cleaning

5. When should handwashing be done?
   a) Before and after examining a patient/client
   b) Before and after using gloves
   c) After contact with blood or other body fluids, or soiled instruments
   d) All of the above

6. What category of medical waste/contaminated waste should be segregated in the Yellow bag?
   a) Used mutilated catheters I.V bottles and tubes, disinfected plastic gloves, other plastic material
   b) Used swabs/ gauze/ bandage, other items (surgical waste) contaminated with blood
   c) Tubing like I.V. drip sets and different types of Catheters and tubes.
   d) General waste from food

7. Decontamination of surgical instruments by soaking in 0.5% chlorine solution
   a) Completely kills all microorganisms, including bacterial
   b) Rapidly kills viruses such as HIV and Hepatitis B
   c) Does not need to be done if instruments are thoroughly washed and rinsed
   d) Does not kill viruses such as HIV and Hepatitis B

8. 0.5% Chlorine solution for decontamination of used (infectious) items is prepared by:

   a) Addition of 3 table spoon bleaching powder paste to 1 litre water
   b) Addition of 1 teaspoon bleaching powder to 1 litre water and same ratio for larger volumes
   c) Addition of 3 teaspoon Bleaching powder to 10 litre water
   d) Addition of 3 teaspoon bleaching powder paste to 1 litre water and same ratio for larger volumes

9. After conducting a delivery, if you have to dispose placenta, oxytocin syringe and needle respectively- which color bins will you use?

   a) Yellow bin, red bin and white puncture proof container
   b) Yellow bin and red bin
   c) White puncture proof container, Yellow bin and red bin
   d) White puncture proof container and Yellow bin

10. Fresh 0.5% Chlorine solution for decontamination of used (infectious) items is required to be prepared

   a) Every week
   b) Once in two days
   c) Every day
   d) Once in three days

Assessor: …………………………………… Signature: ………………………………… Date: ……………………………………

Supplement to: Taneja G, Sarin E, Bajpayee D, et al. Care around birth approach: a training, mentoring, and quality improvement model to optimize intrapartum and immediate postpartum quality of care. Glob Health Sci Pract. 2021;9(3).
https://doi.org/10.9745/GHSP-D-20-00638

SECTION 2.2: COMPETENCY ASSESSMENT – NEWBORN HEALTH

Supplement to: Taneja G, Sarin E, Bajpayee D, et al. Care around birth approach: a training, mentoring, and quality improvement model to optimize intrapartum and immediate postpartum quality of care. Glob Health Sci Pract. 2021;9(3).
https://doi.org/10.9745/GHSP-D-20-00638

CARE AROUND BIRTH

COMPETENCY ASSESSMENT – NEWBORN HEALTH

State: ……………………………………………… District: …………………………… Block: …………..………………………....

Facility: ……………………………………………………………………………………… Date: ……………………………......

Assessed providers: (1)………Name………./……..Designation……….. (2)………Name………./…….Designation…….

(3)…….Name……/…Designation....…. (4)….....…Name……/……Designation……. (5)……..Name……/……...Designation………

Part A- ESSENTIAL NEWBORN CARE

Guidance for the assessor:

i. Introduce yourself and explain to the labor room staff that the purpose of this exercise is to assess her/his competency on essential care to the newborns. This will eventually help us understand the areas where the support needs to be provided.
ii. Select a place which is well lit and place neonatalie at a hard and elevated surface for conducting the assessment, preferably a newborn care corner.
iii. Make her/him aware of the mannequin and the tools available to assess her competency.
iv. 'S' against each item means that the provider has to explain it by just speaking and 'D' means she/he has to demonstrate that function. 'SD' means the response should be a mix of both but mainly the demonstration.
v. Describe the below mentioned case scenarios to the person being assessed. If she/he has difficulty in understanding the scenario, explain it to her/him again.
vi. Discuss that during demonstration s/he has to demonstrate for both essential newborn care and resuscitation exactly the way she/he deals with a real newborn at her/his facility.

Scenario 1. A mother is about to deliver. For newborn care what are the preparations you do in your labor room?

Activity	Provider 1		Provider 2		Provider 3		Provider 4		Provider 5	
	1=Yes, 2=No (encircle)									
**i. Ensured draught free labor room by switching off fan and closing windows (S)**	**1**	**2**	**1**	**2**	**1**	**2**	**1**	**2**	**1**	**2**
**ii. Switched on the radiant warmer at least 20 minutes before expected time of delivery (S)**	**1**	**2**	**1**	**2**	**1**	**2**	**1**	**2**	**1**	**2**
**iii. Ensured delivery room temperature is >25^{0} C (S)**	**1**	**2**	**1**	**2**	**1**	**2**	**1**	**2**	**1**	**2**
**iv. Ensured availability of components of Newborn Care Corner (SD)**	**1**	**2**	**1**	**2**	**1**	**2**	**1**	**2**	**1**	**2**
□ Shoulder roll	1	2	1	2	1	2	1	2	1	2
□ Bag and mask (0 & 1 Size)	1	2	1	2	1	2	1	2	1	2
□ Mucous Extractor	1	2	1	2	1	2	1	2	1	2
□ Source of oxygen	1	2	1	2	1	2	1	2	1	2
□ Clock with seconds hands	1	2	1	2	1	2	1	2	1	2
**v. Placed two clean towels under the radiant warmer maintaining sterile conditions (S)**	**1**	**2**	**1**	**2**	**1**	**2**	**1**	**2**	**1**	**2**
**vi. Hand washing (D)** (to be demonstrated using soap and water)	**1**	**2**	**1**	**2**	**1**	**2**	**1**	**2**	**1**	**2**
□ Rolled up sleeves, removed watch, bangles and rings	1	2	1	2	1	2	1	2	1	2
□ Wet the hands with water from the tap or being poured by someone using mug or pitcher	1	2	1	2	1	2	1	2	1	2
□ Rolled up the full sleeves up to elbow	1	2	1	2	1	2	1	2	1	2
□ Wet hands and forearms up-to the elbow	1	2	1	2	1	2	1	2	1	2
□ Using plain water and soap, washed parts of the hand in the following sequence: Palms and fingers and web spaces→ Back of hands → Fingers and knuckles→ Thumbs→ Finger tips and nails→ Wrists and forearms up-to elbow	1	2	1	2	1	2	1	2	1	2
□ Rinsed with clean water flowing from the tap or being poured by someone using mug or pitcher	1	2	1	2	1	2	1	2	1	2

Activity	Provider 1		Provider 2		Provider 3		Provider 4		Provider 5	
	1=Yes, 2=No (encircle)									
☐ Were the steps in sequence?	1	2	1	2	1	2	1	2	1	2
**vii. Wore a pair of sterile gloves (S)**	**1**	**2**	**1**	**2**	**1**	**2**	**1**	**2**	**1**	**2**
**viii. Ensured availability of Clean cord ties/ clamps and surgical blade (S)**	**1**	**2**	**1**	**2**	**1**	**2**	**1**	**2**	**1**	**2**
**ix. Functioning of bag and mask checked (D)** (to be demonstrated on bag and mask)	**1**	**2**	**1**	**2**	**1**	**2**	**1**	**2**	**1**	**2**
☐ Felt pressure on the palm when the bag is squeezed	1	2	1	2	1	2	1	2	1	2
☐ Upon squeezing enough the pop off valve opened and makes a sound	1	2	1	2	1	2	1	2	1	2
☐ Checked that the bags re-inflates quickly when you release pressure	1	2	1	2	1	2	1	2	1	2
Whether the above signal functions in bold were done in sequence?	**1**	**2**	**1**	**2**	**1**	**2**	**1**	**2**	**1**	**2**

Scenario 2. A baby has just been delivered. What routine care you will provide to that baby after birth? Demonstrate all the steps using the mannequin and tools provided to you. (Tell during the course that the baby started crying immediately after birth)

Activity	Provider 1		Provider 2		Provider 3		Provider 4		Provider 5	
	1=Yes, 2=No (encircle)									
**i. Called out the time of birth (S)**	**1**	**2**	**1**	**2**	**1**	**2**	**1**	**2**	**1**	**2**
**ii. Delivered the baby over mother's abdomen (S)**	**1**	**2**	**1**	**2**	**1**	**2**	**1**	**2**	**1**	**2**
**iii. Checked for meconium (S)**	**1**	**2**	**1**	**2**	**1**	**2**	**1**	**2**	**1**	**2**
**iv. Checked whether baby is crying or breathing normally (SD)**	**1**	**2**	**1**	**2**	**1**	**2**	**1**	**2**	**1**	**2**
Immediate warmth provided (SD)										
**v. Drying the baby**	**1**	**2**	**1**	**2**	**1**	**2**	**1**	**2**	**1**	**2**
☐ Dried newborn with a warm cloth in the following chronology. Head→ Face→ Neck→ Axilla→ Arms→ Hands→ Chest→ Abdomen→ Groin→ Legs→ Feet→ Back	1	2	1	2	1	2	1	2	1	2
☐ Removed the wet cloth	1	2	1	2	1	2	1	2	1	2
☐ Put the baby between mother's breast in skin to skin contact	1	2	1	2	1	2	1	2	1	2
**vi. Skin to skin contact provided (SD)**	**1**	**2**	**1**	**2**	**1**	**2**	**1**	**2**	**1**	**2**
☐ Place the baby between the mother's breast in a prone position	1	2	1	2	1	2	1	2	1	2
☐ Turn the face of the baby one side	1	2	1	2	1	2	1	2	1	2
☐ Cover the baby's head & back with a warm cloth	1	2	1	2	1	2	1	2	1	2
☐ Cover the mother and baby with an additional blanket	1	2	1	2	1	2	1	2	1	2
**vii. Cord clamping (SD)**	**1**	**2**	**1**	**2**	**1**	**2**	**1**	**2**	**1**	**2**
☐ Delayed the clamping of cord for 1-3 min	1	2	1	2	1	2	1	2	1	2
☐ Tied with thread in two places (2 & 5 cm. from umbilicus)	1	2	1	2	1	2	1	2	1	2
☐ Cut umbilical cord with a clean blade	1	2	1	2	1	2	1	2	1	2
☐ Observed for oozing of blood	1	2	1	2	1	2	1	2	1	2
☐ Did not apply anything on stump	1	2	1	2	1	2	1	2	1	2
**viii. Breast feeding (SD)**	**1**	**2**	**1**	**2**	**1**	**2**	**1**	**2**	**1**	**2**
☐ Looked for baby cues (S)	1	2	1	2	1	2	1	2	1	2
Support the mother to initiate breast feeding										

Supplement to: Taneja G, Sarin E, Bajpayee D, et al. Care around birth approach: a training, mentoring, and quality improvement model to optimize intrapartum and immediate postpartum quality of care. Glob Health Sci Pract. 2021;9(3). https://doi.org/10.9745/GHSP-D-20-00638

Activity	Provider 1		Provider 2		Provider 3		Provider 4		Provider 5	
	1=Yes, 2=No (encircle)									
□ Positioning (D)<br>● The whole body of baby is supported<br>● The head and trunk are straight and in one line (head is not tilted)<br>● Newborn's nose is opposite to the mother's nipple<br>● The body of newborn is close to the mother	1	2	1	2	1	2	1	2	1	2
□ Attachment (S)<br>● The mouth of baby is wide open<br>● The upper part of areola is more visible than the lower part<br>● Lower lip everted<br>● Chin of baby touching the breast	1	2	1	2	1	2	1	2	1	2
□ Advice on advantages of colostrum feeding reinforced (S)	1	2	1	2	1	2	1	2	1	2
**ix. Vitamin K1 administered within one hour of birth (S)**	**1**	**2**	**1**	**2**	**1**	**2**	**1**	**2**	**1**	**2**
□ Deep intramuscular, anterolateral aspect of thigh	1	2	1	2	1	2	1	2	1	2
**The provider spoke about starting the examination around one hour of birth**	**1**	**2**	**1**	**2**	**1**	**2**	**1**	**2**	**1**	**2**
**x. Newborn examination**	**1**	**2**	**1**	**2**	**1**	**2**	**1**	**2**	**1**	**2**
Handwashing before examination **(S)**	1	2	1	2	1	2	1	2	1	2
Examination started at around one hour from birth **(S)**	1	2	1	2	1	2	1	2	1	2
a) Measured respiratory rate (SD)	**1**	**2**	**1**	**2**	**1**	**2**	**1**	**2**	**1**	**2**
□ Exposed chest of the baby	1	2	1	2	1	2	1	2	1	2
□ Counted the breathes for one minute using a watch/ clock	1	2	1	2	1	2	1	2	1	2
b) Weight recorded (SD) (to be demonstrated using a weighing scale)	**1**	**2**	**1**	**2**	**1**	**2**	**1**	**2**	**1**	**2**
□ Set the zero of scale with a cloth placed over the tray	1	2	1	2	1	2	1	2	1	2
□ Placed baby on the scale with minimum clothes, preferably naked	1	2	1	2	1	2	1	2	1	2
□ Read the measurement correctly up to the nearest 50 grams weight	1	2	1	2	1	2	1	2	1	2
□ Removed the baby from the tray, wrap the baby immediately	1	2	1	2	1	2	1	2	1	2
□ Record the weight	1	2	1	2	1	2	1	2	1	2
c) Temperature recorded (SD)	**1**	**2**	**1**	**2**	**1**	**2**	**1**	**2**	**1**	**2**
□ Place the silver end of the bulb vertically in the middle of the armpit, under the baby's arm.	1	2	1	2	1	2	1	2	1	2
□ Gently hold the baby's arm against the body	1	2	1	2	1	2	1	2	1	2
□ Remove the thermometer after for 3-5minutes	1	2	1	2	1	2	1	2	1	2
□ Read and record the temperature	1	2	1	2	1	2	1	2	1	2
d) Head to toe examination (SD)	**1**	**2**	**1**	**2**	**1**	**2**	**1**	**2**	**1**	**2**
□ Assessed general appearance alertness and tone	1	2	1	2	1	2	1	2	1	2
□ Check for sex of the baby	1	2	1	2	1	2	1	2	1	2
□ Examined head for<br>• Fontanelles and sutures<br>• Cleft lip<br>• Cleft palate	1	2	1	2	1	2	1	2	1	2
□ Examined eyes for redness and discharge	1	2	1	2	1	2	1	2	1	2
□ Examined skin for jaundice, pallor and cyanosis	1	2	1	2	1	2	1	2	1	2
□ Inspected skin for sores or breaks	1	2	1	2	1	2	1	2	1	2
□ Examined for muscle tone	1	2	1	2	1	2	1	2	1	2
□ Palpated abdomen and liver	1	2	1	2	1	2	1	2	1	2

Activity	Provider 1		Provider 2		Provider 3		Provider 4		Provider 5	
	1=Yes, 2=No (encircle)									
□ Examined extremities, skeletal system<br>• Club hands<br>• Club feet	1	2	1	2	1	2	1	2	1	2
□ Other congenital anomalies like<br>• Spina bifida	1	2	1	2	1	2	1	2	1	2
Whether the above signal functions in bold were done in sequence?	**1**	**2**	**1**	**2**	**1**	**2**	**1**	**2**	**1**	**2**

Scenario 3. What is the care you will provide after completion of initial care till the discharge of the baby?

Activity	Provider 1		Provider 2		Provider 3		Provider 4		Provider 5	
	1=Yes, 2=No (encircle)									
**i. Vaccination done (S)**	**1**	**2**	**1**	**2**	**1**	**2**	**1**	**2**	**1**	**2**
□ Administered BCG intradermal left arm	1	2	1	2	1	2	1	2	1	2
□ Administered birth dose of Hepatitis B (IM)- within 24 hrs	1	2	1	2	1	2	1	2	1	2
□ Administered zero dose OPV (Oral)	1	2	1	2	1	2	1	2	1	2
**ii. Counselling at discharge done (S)**	**1**	**2**	**1**	**2**	**1**	**2**	**1**	**2**	**1**	**2**
□ Instructed the mothers about the benefits of breast-feeding	1	2	1	2	1	2	1	2	1	2
□ Exclusive breast feeding	1	2	1	2	1	2	1	2	1	2
□ Maintenance of hygiene	1	2	1	2	1	2	1	2	1	2
□ Do not apply anything on the cord and keep the cord and umbilicus dry	1	2	1	2	1	2	1	2	1	2
□ Counsel about immunization of baby at age of 6, 10 & 14 weeks	1	2	1	2	1	2	1	2	1	2
**iii. When to return (S)**	**1**	**2**	**1**	**2**	**1**	**2**	**1**	**2**	**1**	**2**
• Breastfeeding or drinking poorly	1	2	1	2	1	2	1	2	1	2
• Convulsions	1	2	1	2	1	2	1	2	1	2
• Fast breathing	1	2	1	2	1	2	1	2	1	2
• Difficult breathing	1	2	1	2	1	2	1	2	1	2
• Develops a fever or feels cold to touch	1	2	1	2	1	2	1	2	1	2
• Diarrhea	1	2	1	2	1	2	1	2	1	2
• Blood in stools	1	2	1	2	1	2	1	2	1	2
• Loss of consciousness	1	2	1	2	1	2	1	2	1	2

Supplement to: Taneja G, Sarin E, Bajpayee D, et al. Care around birth approach: a training, mentoring, and quality improvement model to optimize intrapartum and immediate postpartum quality of care. Glob Health Sci Pract. 2021;9(3). https://doi.org/10.9745/GHSP-D-20-00638

Part B- RESUSCITATION

Notes for the assessor:

i. Describe to the person being assessed in a polite manner that this exercise is to check her competency on resuscitation.
ii. Demonstrate, how chest rise and cord pulsations can be checked on a mannequin.
iii. If the assessor uses bag and mask to ventilate, then observe till the first one minute and then ask to stop and let her complete rest of the steps.

Scenario: A baby has just been delivered in your labor room. The baby is not crying and is meconium stained. What steps would you take to resuscitate the baby? Demonstrate the steps on this mannequin, using the available aids.

Activity	Provider [illegible]		Provider [illegible]		Provider [illegible]		Provider [illegible]		Provider [illegible]	
	1=Yes, 2=No (encircle)									
**i. Called out time of birth (S)**	**1**	**2**	**1**	**2**	**1**	**2**	**1**	**2**	**1**	**2**
**ii. Checked for crying**	**1**	**2**	**1**	**2**	**1**	**2**	**1**	**2**	**1**	**2**
**iii. (Baby not crying) Suction immediately: mouth first and then nostrils**	**1**	**2**	**1**	**2**	**1**	**2**	**1**	**2**	**1**	**2**
**iv. Dried baby thoroughly (D)**	**1**	**2**	**1**	**2**	**1**	**2**	**1**	**2**	**1**	**2**
**v. Assessed for breathing (SD)**										
□ Checked whether the baby was crying or breathing normally by observing chest movements or listening to breathing sounds	1	2	1	2	1	2	1	2	1	2
Baby not crying/ breathing										
**vi. Quickly placed two forceps/ ties (anywhere on the cord) and cut (SD)**	**1**	**2**	**1**	**2**	**1**	**2**	**1**	**2**	**1**	**2**
**vii. Moved baby to the newborn care corner (SD)**	**1**	**2**	**1**	**2**	**1**	**2**	**1**	**2**	**1**	**2**
**viii. Positioning (D)**	**1**	**2**	**1**	**2**	**1**	**2**	**1**	**2**	**1**	**2**
□ Placed shoulder roll	1	2	1	2	1	2	1	2	1	2
□ Positioned the head in sniffing position	1	2	1	2	1	2	1	2	1	2
**ix. Suction (D)**	**1**	**2**	**1**	**2**	**1**	**2**	**1**	**2**	**1**	**2**
□ Suctioned first mouth then both nostrils	1	2	1	2	1	2	1	2	1	2
**x. Stimulation (rubbing at back and flickering at soles) (D)**	**1**	**2**	**1**	**2**	**1**	**2**	**1**	**2**	**1**	**2**
**xi. Repositioning (D)**	**1**	**2**	**1**	**2**	**1**	**2**	**1**	**2**	**1**	**2**
□ Placed shoulder roll	1	2	1	2	1	2	1	2	1	2
□ Positioned the head in sniffing position	1	2	1	2	1	2	1	2	1	2
**xii. Checked if the selected mask in the bag and mask assembly covered chin, mouth & nose (D)**	**1**	**2**	**1**	**2**	**1**	**2**	**1**	**2**	**1**	**2**
**xiii. Applied face mask firmly covering chin, mouth & nose (D)**	**1**	**2**	**1**	**2**	**1**	**2**	**1**	**2**	**1**	**2**
**xiv. Ventilate through bag and mask (D)**	**1**	**2**	**1**	**2**	**1**	**2**	**1**	**2**	**1**	**2**
□ Ventilation started	1	2	1	2	1	2	1	2	1	2
□ Checked for the chest rise	1	2	1	2	1	2	1	2	1	2
□ For each ventilation cycle spoke "Breathe two-three"	1	2	1	2	1	2	1	2	1	2
□ Ventilated for thirty seconds	1	2	1	2	1	2	1	2	1	2
**xv. Assessed the baby (D)**	**1**	**2**	**1**	**2**	**1**	**2**	**1**	**2**	**1**	**2**
□ Assessed for breathing	1	2	1	2	1	2	1	2	1	2
Baby still not crying/ breathing (prompt to the provider)										
**xvi. Called for help (S)**	**1**	**2**	**1**	**2**	**1**	**2**	**1**	**2**	**1**	**2**
□ Asked to arrange for vehicle for transportation and inform the doctor	1	2	1	2	1	2	1	2	1	2
**xvii. Attached oxygen to the bag and mask assembly (D)**	**1**	**2**	**1**	**2**	**1**	**2**	**1**	**2**	**1**	**2**

Supplement to: Taneja G, Sarin E, Bajpayee D, et al. Care around birth approach: a training, mentoring, and quality improvement model to optimize intrapartum and immediate postpartum quality of care. Glob Health Sci Pract. 2021;9(3).
https://doi.org/10.9745/GHSP-D-20-00638

Activity	Provider		Provider		Provider		Provider		Provider	
	1=Yes, 2=No (encircle)									
**xviii. Continued bag and mask ventilation (D)**	**1**	**2**	**1**	**2**	**1**	**2**	**1**	**2**	**1**	**2**
**xix. Checked heart rate (D)**	**1**	**2**	**1**	**2**	**1**	**2**	**1**	**2**	**1**	**2**
Heart rate below 100 beats per minute (prompt to the provider)										
**xx. Continued bag and mask ventilation with oxygen (D)**	**1**	**2**	**1**	**2**	**1**	**2**	**1**	**2**	**1**	**2**
**xxi. Checked heart rate after next 30 seconds**	**1**	**2**	**1**	**2**	**1**	**2**	**1**	**2**	**1**	**2**
After one minute from the start of bag and mask ventilation the baby started breathing and maintained 100 beats per minute (prompt to the provider)										
**xxii. Gradually stopped ventilation (D)**	**1**	**2**	**1**	**2**	**1**	**2**	**1**	**2**	**1**	**2**
**xxiii. Gave baby to the mother (S)**	**1**	**2**	**1**	**2**	**1**	**2**	**1**	**2**	**1**	**2**
**xxiv. Monitored the baby with the mother (S)**	**1**	**2**	**1**	**2**	**1**	**2**	**1**	**2**	**1**	**2**
□ Breathing: rate and regularity (normal: 40-60 per minute & regular)	1	2	1	2	1	2	1	2	1	2
□ Heart Rate: rate (normal: >100 per minute)	1	2	1	2	1	2	1	2	1	2
□ Temperature: within the normal range (normal: 36.5-37.5^{0}C)	1	2	1	2	1	2	1	2	1	2
□ Color (normal: pink /// abnormal: blue or pale)	1	2	1	2	1	2	1	2	1	2
Whether all the above signal functions both bold and non-bold were done in sequence?	**1**	**2**	**1**	**2**	**1**	**2**	**1**	**2**	**1**	**2**

Assessor: ……………………………………. Signature: ………………………………… Date: ……………………………………

Supplement to: Taneja G, Sarin E, Bajpayee D, et al. Care around birth approach: a training, mentoring, and quality improvement model to optimize intrapartum and immediate postpartum quality of care. Glob Health Sci Pract. 2021;9(3).
https://doi.org/10.9745/GHSP-D-20-00638

SECTION 3: BASELINE ASSESSMENT 3: RECORD REVIEW

Supplement to: Taneja G, Sarin E, Bajpayee D, et al. Care around birth approach: a training, mentoring, and quality improvement model to optimize intrapartum and immediate postpartum quality of care. Glob Health Sci Pract. 2021;9(3). https://doi.org/10.9745/GHSP-D-20-00638

CARE AROUND BIRTH

INDICATORS FOR BASELINE RECORD REVIEW

State: …………………………… District: ……………………….. Block: ……………………. Facility ……………………………….
Facility type : DH/SDH/ RH/ Area Hospital/FRU-CHC/non-FRU CHC/24 x7 PHC/PHC (L1)/HSC
Facility level: L3/L2/L1 Date: …………

The following document includes the list of indicators to be collected on a monthly basis from the project intervention facilities.

- A baseline for the indicators is being generated through the current exercise
- The reference period for the baseline is October – December 2015 (calendar month should be considered for the reporting cycle)
- The reference for the data should be the primary data source (for instance Labour room register / case sheet)
- If any of the following indicators are not available then the same should be recorded as "NA" (not available)

Section 1: Demographic profile of pregnant women admitted to labour rooms (data to be collated from the Labour Room register)

Age profile:

Age group (in completed years)	Oct 2015	Nov 2015	Dec 2015
15-19			
20-24			
25-29			
30-34			
>35			

(NA to be entered if relevant data is not available)

Education status:

Education	Oct 2015	Nov 2015	Dec 2015
Illiterate			
Primary school (up to class 5) or < 5 years of schooling			
Secondary school (up to class 8) or 5-8 years of schooling			
Higher secondary (up to class 12) or 8-12 years of schooling			
Graduate			
Post graduate			

(NA to be entered if relevant data is not available)

Supplement to: Taneja G, Sarin E, Bajpayee D, et al. Care around birth approach: a training, mentoring, and quality improvement model to optimize intrapartum and immediate postpartum quality of care. Glob Health Sci Pract. 2021;9(3). https://doi.org/10.9745/GHSP-D-20-00638

Religion:

Religion	Oct 2015	Nov 2015	Dec 2015
Hindu			
Muslim			
Christian			
Sikh			
Others			

(NA to be entered if relevant data is not available)

Category:

Category	Oct 2015	Nov 2015	Dec 2015
General			
OBC / EBC			
Scheduled Caste			
Scheduled Tribe			

(NA to be entered if relevant data is not available)

Economic status:

Category	Oct 2015	Nov 2015	Dec 2015
Below Poverty Line (BPL)			
Above Poverty Line (APL)			

Section 2: Service delivery indicators: Maternal and Newborn Health (data to be collated from the Labour Room register, please refer to the relevant sections where additional data sources can be referred to)

S. no	Indicator	Definition	Oct 2015	Nov 2015	Dec 2015
1	**Deliveries**	Total deliveries conducted during the month in the facility with breakup of individual methods as cited below			
1.1	• Vaginal				
1.2	• Assisted vaginal	An assisted vaginal delivery (AVD) occurs when a pregnant female goes into labour (with or without the use of drugs or techniques to induce labour), and requires the use of special instruments such as forceps or a vacuum extractor to deliver her baby vaginally.			
1.3	• C-section				
2	**Live births**	Total number of live births registered in the facility			
3	**Still births**	Total number of still births registered in the facility			
3.1	• Fresh	Skin still intact, death occurred less than 12 hours before delivery			

3.2	• Macerated	Skin not intact, death occurred more than 12 hours before delivery			
4	**Intrapartum care practices**				
4.1	• Partograph filled to monitor progress of labour	Number of deliveries in which Partograph was filled out of the total deliveries conducted			
4.2	• Injection oxytocin administered during AMTSL[1]	Number of deliveries in which oxytocin was administered during AMTSL out of the total deliveries conducted			
4.3	• Injection oxytocin administered within a minute of delivery during AMTSL	Number of deliveries in which oxytocin was administered within one minute of delivery during AMTSL out of the total deliveries conducted			
4.4	• Number of complications recorded	Number of complications as listed below recorded during the month			
4.4.1	○ Obstructed / prolonged labour				
4.4.2	○ High BP / pre-eclampsia / eclampsia				
4.4.3	○ Post-Partum Haemorrhage				
5	**Essential Newborn Care**				
5.1	Newborn weighed at birth	Number of newborns weighed at birth out of total live births			
5.2	Low birth weight babies (<2.5 kg) registered	Number of low birth weight babies (<2.5 kg) registered out of total live births			
5.3	Low birth weight babies (<2.0 kg) registered	Number of low birth weight babies (<2.0 kg) registered out of total live births			
5.4	Newborns in whom temperature was recorded at birth	Number of newborns in whom body temperature was recorded at birth out of total live births			
5.5	Newborns who were dried as per ENC[2] guidelines after birth	Number of newborns who were dried after birth out of total live births			
5.6	Newborns in whom delayed cord clamping (in 1-3 minutes) was practiced following birth	Number of newborns in whom delayed cord clamping was practiced out of total live births			
5.7	Newborns who were administered Vit K1 following birth	Number of newborns who were administered Vit K1 after birth out of total live births			
5.8	Newborns where breast feeding was initiated within one hour of birth	Number of newborns in whom breast feeding was initiated within an hour of birth out of total live births			

[1] AMTSL : Active Management of Third Stage of Labour
[2] ENC : Essential Newborn Care

5.9	Newborns < 2kg initiated Kangaroo Mother Care (if recorded state the source)	Number of newborns weighing < 2 kg who were provided prolonged skin to skin care for at least one hour during first 24 hours of life			
6	**Newborn Vaccination (a separate newborn vaccination register may be referred to)**				
6.1	Newborns who were administered Hepatitis B birth dose within 24 hours of birth	Number of newborns who were administered Hepatitis B birth dose within 24 hours of birth			
6.2	Newborns who were administered OPV[3] zero dose before discharge	Number of newborns who were administered OPV zero dose before discharge out of total live births			
6.3	Newborns who were administered BCG[4] before discharge	Number of newborns who were administered BCG before discharge out of total live births			
6.4	Newborns who were administered all three vaccines before discharge	Number of newborns who were administered all three vaccines before discharge out of total live births			
7	**Newborn Resuscitation**				
7.1	Newborns identified with asphyxia at birth	Number of newborns who were identified with birth asphyxia out of total live births			
7.2	Newborns successfully resuscitated	Number of newborns who were successfully resuscitated out of asphyxiated newborns			
8	**Referrals (separate referral registers may be referred to)**				
8.1	Pregnant women referred in to the facility	Number of pregnant women who were referred to the facility from other facilities during the month			
8.2	Newborns referred in to the facility	Number of newborns who were referred to the facility from other facilities during the month			
8.3	Pregnant women referred out from the facility	Number of pregnant women who were referred from the facility to higher facilities during the month			
8.4	Newborns referred out from the facility	Number of newborns who were referred from the facility to higher facilities during the month			
**9**	**Deaths (separate death registers may be referred to)**				
9.1	Maternal deaths reported in the facility	Number of maternal deaths reported in the facility during the month			
9.2	Newborn deaths reported in the facility	Number of newborn deaths reported in the facility during the month			

[3] OPV: Oral Polio Vaccine
[4] BCG: Bacillus Calmatte Guerin

10	**Stock outs***				
10.1	Stock out of oxytocin	Stock out of oxytocin reported in the facility even for a single day during the month			
10.2	Stock out of Magnesium Sulphate	Stock out of Magnesium Sulphate reported in the facility even for a single day during the month			
10.3	Stock out of Vitamin K1	Stock out of Vitamin K1 reported in the facility even for a single day during the month			
10.4	Stock out of BCG	Stock out of BCG reported in the facility even for a single day during the month			
10.5	Stock out of OPV	Stock out of OPV reported in the facility even for a single day during the month			
10.6	Stock out of Hepatitis B	Stock out of Hepatitis B reported in the facility even for a single day during the month			

*(To be checked in the pharmacy / store, put in Yes / No depending upon the status)

Section 3: Verification of last 10 Partographs used in L3 facilities and last 5 Partographs used in L2 / L1 facilities during the month of December 2015:

Parameter	Case 1	Case 2	Case 3	Case 4	Case 5	Case 6	Case 7	Case 8	Case 9	Case 10
Partograph filled completely (check whether all components have been filled as per guidelines)[5]										

(Put in yes / no in each column as per filling up of relevant parameters in each Partograph)

Section 4: Verification of case sheets for post natal vital monitoring of last 10 discharged cases in L3 facilities and last 5 discharged cases in L2 facilities during the month of December 2015

Parameter	Case 1	Case 2	Case 3	Case 4	Case 5	Case 6	Case 7	Case 8	Case 9	Case 10
Number of times mother monitored for Blood Pressure and Pulse, 6 hours post delivery										
Number of times newborn monitored for temperature, breathing within one hour of birth										
Number of times newborn monitored for temperature, breathing, breast feeding and passage of urine and stool within 1-6 hours of birth										

(For each individual monitoring if all the components are monitored only then it is counted)

[5] Fetal Heart Rate, Amniotic Fluid, Cervical dilatation, Uterine contractions, Mother's vitals (Pulse and BP) filled as per desired frequency

Supplement to: Taneja G, Sarin E, Bajpayee D, et al. Care around birth approach: a training, mentoring, and quality improvement model to optimize intrapartum and immediate postpartum quality of care. Glob Health Sci Pract. 2021;9(3).
https://doi.org/10.9745/GHSP-D-20-00638

Section 5: Verification of case sheets for monitoring at discharge of last 10 discharged cases in L3 facilities and last 5 discharged cases in L2 facilities during the month of December 2015

Parameter	Case 1	Case 2	Case 3	Case 4	Case 5	Case 6	Case 7	Case 8	Case 9	Case 10
Maternal Blood Pressure, pulse and temperature monitored at time of discharge										
Newborns monitored for temperature, breathing and feeding at time of discharge										

(Put in yes / no in each column as per filling up of relevant parameters in each Partograph. A yes needs to be put only if all the parameters were monitored)
