# Supplementary material for: Care Around Birth Approach: A Training, Mentoring, and Quality Improvement Model to Optimize Intrapartum and Immediate Postpartum Quality of Care in India
Source: Glob Health Sci Pract. 2021 Sep 30;9(3):590–610. doi: 10.9745/GHSP-D-20-00368 (PMC8514027; doi:10.9745/GHSP-D-20-00368)
Supplement: 20-00368-Sarin-Supplement2.pdf [file 20-00368-Sarin-Supplement2.pdf]

Supplement to: Taneja G, Sarin E, Bajpayee D, et al. Care around birth approach: a training, mentoring, and quality improvement model to optimize intrapartum and immediate postpartum quality of care. Glob Health Sci Pract. 2021;9(3).
https://doi.org/10.9745/GHSP-D-20-00368

USAID - VRIDDHI (Scaling up RMNCH+A Interventions) Project

CARE AROUND BIRTH

MONTHLY MIS DATA SHEET

State: …………………….. District: ……………………….. Block: ……………………. Facility: ……………………………….
Facility type: DH/SDH/ RH/ Area Hospital/FRU-CHC/non-FRU CHC/24 x7 PHC/PHC (L1)/HSC
Facility level: L3/L2/L1 Date: ……………………..

The following document includes the list of indicators to be collected on a monthly basis from the project intervention facilities.

- The reference for the data should be the primary data source (for instance Labour room register / case sheet)
- Data needs to be collected on a monthly basis with the reference period being 1st – 30th /31st of the month
- If any of the following indicators are not available then the same should be recorded as "NA" (not available)

Section 1: Service delivery indicators: Maternal and Newborn Health (data to be collated from the Labour Room register, please refer to the relevant sections where additional data sources can be referred to)

S. no	Indicator	Definition	Month: _____2018
1	**Deliveries**	Total deliveries conducted during the month in the facility with breakup of individual methods as cited below	
1.1	• Vaginal		
1.2	• Assisted vaginal	An assisted vaginal delivery (AVD) occurs when a pregnant female goes into labour (with or without the use of drugs or techniques to induce labour), and requires the use of special instruments such as forceps or a vacuum extractor to deliver her baby vaginally.	
1.3	• C-section		
2	**Live births**	Total number of live births registered in the facility	
3	**Still births**	Total number of still births registered in the facility	
3.1	• Fresh	Skin still intact, death occurred less than 12 hours before delivery	
3.2	• Macerated	Skin not intact, death occurred more than 12 hours before delivery	

4	**Intrapartum care practices**		
4.1	• Partograph filled to monitor progress of labour	Number of deliveries in which Partograph was filled out of the total deliveries conducted	
4.2	• Injection oxytocin administered during AMTSL[1]	Number of deliveries in which oxytocin was administered during AMTSL out of the total deliveries conducted	
4.3	• Injection oxytocin administered within a minute of delivery during AMTSL	Number of deliveries in which oxytocin was administered within one minute of delivery during AMTSL out of the total deliveries conducted	
4.4	• Number of complications recorded	Number of complications as listed below recorded during the month	
4.4.1	○ Obstructed / prolonged labour		
4.4.2	○ High BP / pre-eclampsia / eclampsia		
4.4.3	○ Post-Partum Haemorrhage		
5	**Essential Newborn Care**		
5.1	Newborn weighed at birth	Number of newborns weighed at birth out of total live births	
5.2	Low birth weight babies (<2.5 kg) registered	Number of low birth weight babies (<2.5 kg) registered out of total live births	
5.3	Low birth weight babies (<2.0 kg) registered	Number of low birth weight babies (<2.0 kg) registered out of total live births	
5.4	Newborns in whom temperature was recorded at birth	Number of newborns in whom body temperature was recorded at birth out of total live births	
5.5	Newborns who were dried as per ENC[2] guidelines after birth	Number of newborns who were dried after birth out of total live births	
5.6	Newborns in whom delayed cord clamping (in 1-3 minutes) was practiced following birth	Number of newborns in whom delayed cord clamping was practiced out of total live births	
5.7	Newborns who were administered Vit K1 following birth	Number of newborns who were administered Vit K1 after birth out of total live births	

[1] AMTSL : Active Management of Third Stage of Labour
[2] ENC : Essential Newborn Care

5.8	Newborns where breast feeding was initiated within one hour of birth	Number of newborns in whom breast feeding was initiated within an hour of birth out of total live births	
5.9	Newborns < 2kg initiated Kangaroo Mother Care (if recorded state the source)	Number of newborns weighing < 2 kg who were provided prolonged skin to skin care for at least one hour during first 24 hours of life	
6	**Newborn Vaccination (separate newborn vaccination register may be referred to)**		
6.1	Newborns who were administered Hepatitis B birth dose within 24 hours of birth	Number of newborns who were administered Hepatitis B birth dose within 24 hours of birth	
6.2	Newborns who were administered OPV[3] zero dose before discharge	Number of newborns who were administered OPV zero dose before discharge out of total live births	
6.3	Newborns who were administered BCG[4] before discharge	Number of newborns who were administered BCG before discharge out of total live births	
6.4	Newborns who were administered all three vaccines before discharge	Number of newborns who were administered all three vaccines before discharge out of total live births	
7	**Newborn Resuscitation**		
7.1	Newborns identified with asphyxia at birth	Number of newborns who were identified with birth asphyxia out of total live births	
7.2	Newborns successfully resuscitated	Number of newborns who were successfully resuscitated out of asphyxiated newborns	
8	**Referrals (separate referral registers may be referred to)**		
8.1	Pregnant women referred in to the facility	Number of pregnant women who were referred to the facility from other facilities during the month	
8.2	Newborns referred in to the facility	Number of newborns who were referred to the facility from other facilities during the month	
8.3	Pregnant women referred out from the facility	Number of pregnant women who were referred from the facility to higher facilities during the month	
8.4	Newborns referred out from the facility	Number of newborns who were referred from the facility to higher facilities during the month	
9	**Maternal and newborn deaths**		
9.1	Maternal deaths reported in the facility	Number of maternal deaths reported in the facility during the month	

[3] OPV: Oral Polio Vaccine
[4] BCG: Bacillus Calmatte Guerin

9.2	Newborn deaths reported in the facility	Number of newborn deaths reported in the facility during the month	
10	**Stock outs**		
10.1	Stock out of oxytocin	Stock out of oxytocin reported in the facility even for a single day during the month	
10.2	Stock out of Magnesium Sulphate	Stock out of Magnesium Sulphate reported in the facility even for a single day during the month	
10.3	Stock out of Vitamin K1	Stock out of Vitamin K1 reported in the facility even for a single day during the month	
10.4	Stock out of BCG	Stock out of BCG reported in the facility even for a single day during the month	
10.5	Stock out of OPV	Stock out of OPV reported in the facility even for a single day during the month	
10.6	Stock out of Hepatitis B	Stock out of Hepatitis B reported in the facility even for a single day during the month	

*(To be checked in the pharmacy / store, put in Yes / No depending upon the status)

Section 2: Verification of last 10 Partographs used in L3 facilities and last 5 Partographs used in L2 / L1 facilities in the month:

Parameter	Case 1	Case 2	Case 3	Case 4	Case 5	Case 6	Case 7	Case 8	Case 9	Case 10
Partograph filled completely (check whether all components have been filled as per guidelines)[5]										

(Put in yes / no in each column as per filling up of relevant parameters in each Partograph)

[5] Fetal Heart Rate, Amniotic Fluid, Cervical dilatation, Uterine contractions, Mother's vitals (Pulse and BP) filled as per desired frequency

Supplement to: Taneja G, Sarin E, Bajpayee D, et al. Care around birth approach: a training, mentoring, and quality improvement model to optimize intrapartum and immediate postpartum quality of care. Glob Health Sci Pract. 2021;9(3). https://doi.org/10.9745/GHSP-D-20-00368

Section 3: Verification of case sheets for post natal vital monitoring of last 10 discharged cases in L3 facilities and last 5 discharged cases in L2 facilities during the month:

Parameter	Case 1	Case 2	Case 3	Case 4	Case 5	Case 6	Case 7	Case 8	Case 9	Case 10
Number of times mother monitored for Blood Pressure and Pulse, 6 hours post delivery										
Number of times newborn monitored for temperature, breathing within one hour of birth										
Number of times newborn monitored for temperature, breathing, breast feeding and passage of urine and stool within 1-6 hours of birth										

(For each individual monitoring if all the components are monitored only then it is counted)

Section 4: Verification of case sheets for monitoring at discharge of last 10 discharged cases in L3 facilities and last 5 discharged cases in L2 facilities during the month:

Parameter	Case 1	Case 2	Case 3	Case 4	Case 5	Case 6	Case 7	Case 8	Case 9	Case 10
Maternal Blood Pressure, pulse and temperature monitored at time of discharge										
Newborns monitored for temperature, breathing and feeding at time of discharge										

(Put in yes / no in each column as per filling up of relevant parameters in each Partograph. A yes needs to be put only if all the parameters were monitored)

Indicator	Month/ Year: …………………………….
No. of QI Meetings held during the month:	

DTO: ………………………………….   Signature: ………………………………   Date: ……………………………………
